# Cotargeting DNA topoisomerase II enhances efficacy of RAS-targeted therapy in KRAS-mutant cancer models

**DOI:** 10.1172/JCI197192

**Published:** 2026-02-16

**Authors:** Rongzhong Xu, Dongsheng Wang, Guangzhi Ma, Xun Yuan, Qian Chu, Songqing Fan, Rener Zhang, Du, Shidong Jia, Ticiana A. Leal, Suresh S. Ramalingam, Zhen Chen, Shi-Yong Sun

**Affiliations:** 1 Clinical Medical Center of Oncology, Shanghai Municipal Hospital of Traditional Chinese Medicine, Shanghai University of Traditional Chinese Medicine, Shanghai, China.; 2 Department of Hematology and Medical Oncology, Emory University School of Medicine, Atlanta, Georgia, USA.; 3 Department of Thoracic Surgery and Institute of Thoracic Oncology, West China Hospital, Sichuan University, Chengdu, China.; 4 Department of Oncology, Tongji Hospital, Tongji Medical College, Huazhong University of Science and Technology, Wuhan, China.; 5 Department of Pathology, The Second Xiangya Hospital, Central South University, Changsha, China.; 6 Predicine, Inc., Hayward, California, USA.; 7 Winship Cancer Institute of Emory University, Atlanta, Georgia, USA.

**Keywords:** Cell biology, Oncology, Apoptosis, Drug therapy, Lung cancer

## Abstract

The approval of sotorasib and adagrasib as the first KRAS G12C inhibitors has made the RAS oncogene a druggable target. However, they have modest objective response rates and short response durations. Therefore, strategies for improving RAS-targeted cancer therapy are urgently needed. Here, we found that both sotorasib and adagrasib promoted topoisomerase IIα (Topo IIα) proteasomal degradation in KRAS G12C–mutant cancer cells and induced DNA damage and apoptosis. In cell lines with acquired resistance to sotorasib, elevated Topo IIα levels were detected. *TOP2A* overexpression in sensitive KRAS G12C–mutant cells conferred resistance to sotorasib, whereas *TOP2A* knockdown in sotorasib-resistant cell lines sensitized the cells to sotorasib. Moreover, the combination of a KRAS G12C inhibitor such as sotorasib with a Topo II inhibitor such as VP-16 synergistically decreased the survival of sotorasib-resistant RAS G12C–mutant cells with augmented induction of DNA damage and apoptosis, effectively inhibited the growth of sotorasib-resistant tumors, and delayed or prevented the emergence of acquired resistance to sotorasib in vivo. Collectively, our results reveal an essential role of Topo IIα inhibition in mediating the therapeutic efficacy of RAS-targeted cancer therapy, providing a strong scientific rationale for targeting Topo II to improve RAS-targeted cancer therapies.

## Abstract 2

Topo IIa and RAS-targeted cancer therapy

## Graphical abstract

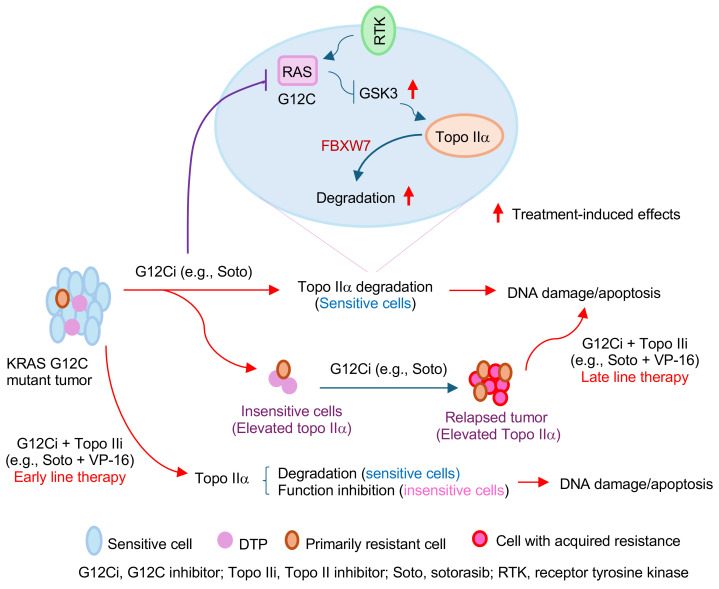

## Introduction

The RAS gene family, comprising HRAS, NRAS, and KRAS, has been identified as one of the most important oncogene groups in cancer, among which, KRAS is the most frequently mutated, accounting for approximately 80% of all RAS mutations (1). The most common and lethal cancer types, such as colorectal cancer, pancreatic ductal adenocarcinoma, and non–small cell lung cancer (NSCLC), are frequently associated with KRAS mutations (43%, 85%, and 31%, respectively) (1). The seminal discovery in 2013 of a druggable pocket within the switch region in the KRAS G12C mutant and demonstration of selective inhibition of KRAS G12C–mutant cancer cells with a small molecule compound that could covalently bind to the pocket by Ostrem et al. (2) suggested the possibility to target the previously “undruggable” KRAS for therapy of KRAS-mutant cancer types. Subsequent efforts resulted in FDA approval of the first covalently binding KRAS G12C inhibitors, sotorasib and adagrasib, in 2021 and 2022, respectively (1).

The KRAS G12C mutation accounts for about 30% of KRAS mutation in NSCLC, making NSCLC a cancer subtype with predominant G12C mutation (1, 3). Accordingly, both sotorasib and adagrasib have been approved by the FDA to treat patients with advanced NSCLC carrying KRAS G12C mutation who have received at least 1 prior therapy. However, these inhibitors as monotherapy in second-line treatment in general have relatively lower overall objective response rate (ORR) and shorter progression free survival (PFS) compared with other approved targeted therapies and failed to demonstrate improvement in overall survival compared with docetaxel in phase III studies (4, 5), largely due to primary resistance or early emergence of acquired resistance (1, 3). This is particularly the case in colorectal cancer with KRAS G12C mutation (1). Therefore, efforts have been made to improve the therapeutic outcome of RAS-targeted cancer therapy through various approaches, including the development of next-generation KRAS G12C inhibitors and combination regimens (1, 3). Consequently, many clinical trials that aim to test these approaches have been conducted or are ongoing, albeit with limited breakthrough outcomes (1). Hence, the development of efficacious mechanism-driven novel therapeutic strategies for overcoming resistance to RAS-targeted therapy, including primary and acquired resistance that limit the therapeutic outcomes of treatment, is urgently needed.

Topoisomerase II (Topo II), an important enzyme that regulates DNA topology and is critical for DNA replication and transcription, has long been recognized as a valid cancer target (6). Chemotherapeutic agents such as doxorubicin (DXR) and VP-16 (etoposide) act through inhibition of Topo II by trapping Topo II onto DNA to increase the levels of Topo II–DNA covalent complexes, leading to prevention of DNA replication and transcription, as well as subsequent DNA single- and double-strand breaks (or DNA damage) that in turn cause apoptosis when not adequately repaired (7, 8). Our recent work has revealed an important connection between Topo IIα inhibition and cellular response to EGFR-targeted therapy in NSCLC with mutant EGFR (9). We demonstrated that the third-generation EGFR–tyrosine kinase inhibitor osimertinib decreases Topo IIα levels through promoting GSK3-dependent and FBXW7-mediated proteasomal degradation, resulting in induction of DNA damage and subsequent apoptosis in EGFR-mutant NSCLC cells. Upon acquiring resistance, EGFR-mutant NSCLC cells possess elevated levels of Topo IIα, which are resistant to osimertinib modulation. The combination of osimertinib with a Topo II inhibitor (e.g., VP-16) not only enhances therapeutic efficacy when used as a first-line therapy by enhancing the elimination of drug-tolerant persister cells (DTPs) and primarily resistant cell populations and delaying the emergence of acquired resistance, but also restores the response of osimertinib-resistant cells/tumors to treatment when used as a second-line therapy following osimertinib monotherapy exposure. These findings provide a mechanistic insight into EGFR-targeted therapy-induced DNA damage and strong preclinical support for potential clinical trials to validate the strategy of cotargeting EGFR and Topo II in the treatment of patients with EGFR-mutant NSCLC.

RAS protein lies immediately downstream of receptor tyrosine kinases, including EGFR, to mediate the activation of distinct signaling pathways such as PI3K/Akt/mTOR and RAS/MEK/ERK that are critical for cell survival and proliferation (1). Hence, we explored whether Topo IIα inhibition is also an important mechanism that mediates the therapeutic efficacy of RAS-targeted cancer therapy. Using sotorasib and adagrasib as model reagents, this study demonstrated a critical role of Topo IIα inhibition in mediating the therapeutic efficacy of RAS-targeted therapy, particularly in NSCLC with KRAS G12C mutation. Our promising preclinical findings warrant further investigation in early-phase clinical trials to validate this therapeutic strategy to improve therapeutic outcomes of RAS-targeted cancer therapy via coinhibition of Topo II.

## Results

### KRAS G12C inhibitors that inhibit the growth of NSCLC cell lines harboring KRAS G12C mutation with induction of apoptosis decrease Topo IIα levels and induce DNA damage.

**Figure 1:**
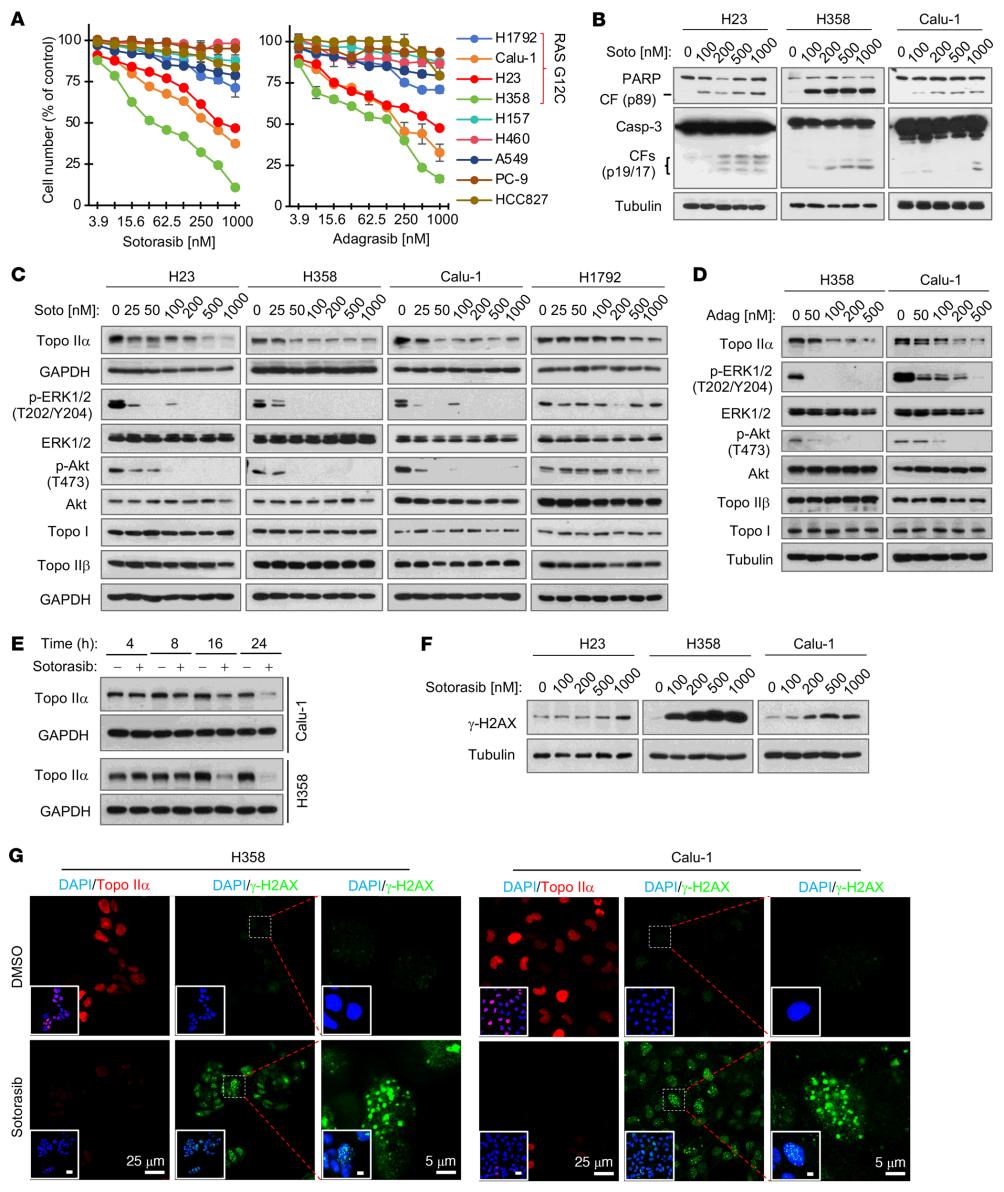
KRAS G12C inhibitors that effectively inhibit the growth and induce apoptosis of NSCLC cell lines harboring KRAS G12C mutation decrease Topo IIα levels with induction of DNA damage. (**A**) The given cell lines were exposed to different concentrations of sotorasib or adagrasib for 72 hours. Cell numbers were then measured by the SRB assay. Data are shown as the mean ± SD of 4 replicate determinations. (**B**–**F**) The cell lines indicated were treated with varied concentrations of sotorasib (Soto) (**B**, **C**, and **F**) or adagrasib (Adag) (**D**) for 24 hours or 500 nM sotorasib for the indicated times (**E**). The proteins of interest were detected with Western blotting. (**G**) The indicated cell lines were exposed to 200 nM (H358) or 500 nM (Calu-1) sotorasib for 24 hours and then stained using IF with anti–γ-H2AX antibody, anti–Topo IIα antibody, and DAPI. The insets are overlay images with DAPI. Scale bars: 25 μm (H358 and Calu-1, bottom left), 5 μm (H358 and Calu-1, bottom right).

We began by validating the effects of the 2 well-known and FDA-approved KRAS G12C inhibitors, sotorasib and adagrasib, on the growth of a panel of NSCLC cell lines, including those with KRAS G12C mutation. While the cell lines without KRAS G12C mutation, H157, H460, A549 and PC-9, were insensitive to both drugs, the cell lines harboring KRAS G12C mutation, including H358, H23, Calu-1, and H1792, were relatively sensitive to the treatments, although H1792 was the least sensitive among them (Figure 1A). Using sotorasib as a representative drug, we detected increased cleavage of caspase-3 and poly(ADP-ribose) polymerase (PARP) in H358, H23, and Calu-1 cells exposed to sotorasib at concentrations ranging from 100 to 1,000 nM for 48 hours (Figure 1B), indicating induction of apoptosis in these sensitive cell lines. As expected, both sotorasib and adagrasib effectively decreased the levels of p-ERK1/2 and p-AKT, particularly in H358, H23, and Calu-1 cell lines (Figure 1, C and D), indicating that they effectively inhibit both MEK/ERK and PI3K/AKT signaling pathways. Hence, we validated the on-target therapeutic effects of both sotorasib and adagrasib in NSCLC cell lines with KRAS G12C mutation.

We then determined whether sotorasib affects the levels of Topo IIα in these KRAS G12C–mutant NSCLC cell lines and found that it decreased them at a concentration as low as 25 or 50 nM in H358, H23, and Calu-1 cell lines, although a high concentration up to 500 nM was needed to decrease Topo IIα in H1792 cells (Figure 1C). Adagrasib at 50 or 100 nM also decreased Topo IIα levels in H358 and Calu-1 cells (Figure 1D). Neither agent affected Topo IIβ or Topo I levels in these cell lines (Figure 1, C and D). Reduction of Topo IIα in sotorasib-treated cell lines occurred early at 8 hours posttreatment and was sustained up to 24 hours (Figure 1E), suggesting an early and sustained event. In other types of cancer cell lines with KRAS G12C mutation, including SW1463 (colon cancer), SW837 (colon cancer), and MiaPaCa (pancreatic cancer), we found that sotorasib decreased Topo IIα levels as well (Supplemental Figure 1A; supplemental material available online with this article; https://doi.org/10.1172/JCI197192DS1). As expected, sotorasib did not decrease Topo IIα levels in NSCLC cell lines without KRAS G12C mutation (Supplemental Figure 1B), further indicating KRAS G12C mutation–dependent activity.

Given that Topo II inhibition causes DNA damage, we then determined whether sotorasib induces DNA damage using increase of γ-H2AX as a marker. As shown in Figure 1F, sotorasib clearly increased the levels of γ-H2AX as detected with Western blotting in these cell lines. Moreover, we detected an increased number of cells positive for γ-H2AX foci formation in these cell lines, whereas Topo IIα levels were clearly decreased, as detected with immunofluorescence (IF) staining (Figure 1G and Supplemental Figure 1C). Hence, it is apparent that sotorasib induces DNA damage in these NSCLC cell lines with KRAS G12 mutation.

### KRAS G12C inhibitors induce GSK3-dependent and FBXW7-mediated Topo IIα proteasomal degradation.

**Figure 2:**
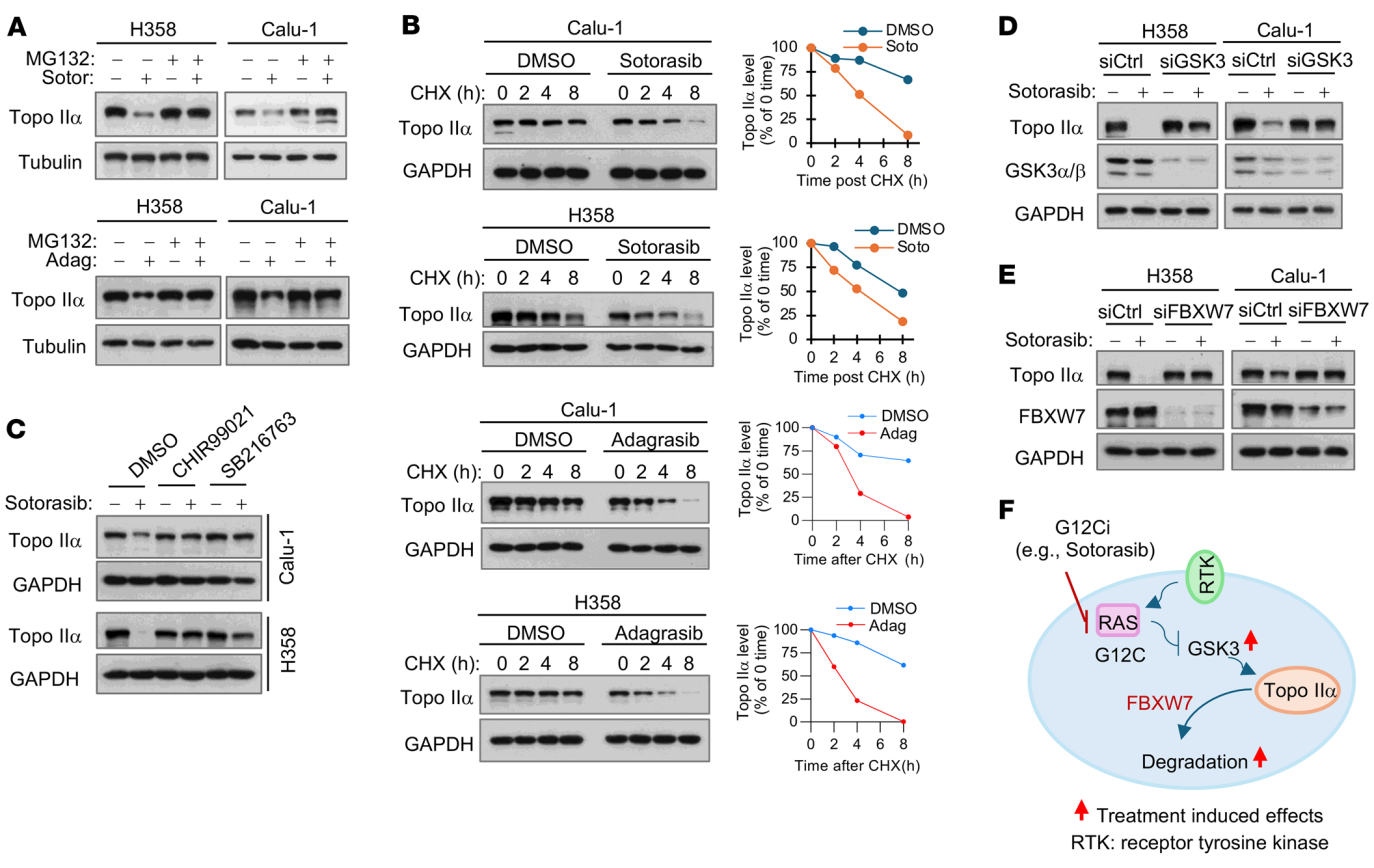
KRAS G12C inhibitors decrease Topo IIα levels through inducing GSK3-dependent and FBXW7-mediated proteasomal degradation. (**A**) The tested cell lines were pretreated with 10 μM MG132 for 30 minutes and then cotreated with DMSO, 500 nM sotorasib (Soto), or 250 nM adagrasib (Adag) for another 6 hours. (**B**) The indicated cell lines were treated with 500 nM sotorasib or adagrasib for 16 hours followed by the addition of 10 μg/mL CHX and then harvested at the indicated times. (**C**) The tested cell lines were pretreated with 10 μM CHIR99021 or SB216763 for 30 minutes and then cotreated with 500 nM sotorasib for an additional 16 hours. (**D** and **E**) Both H358 and Calu-1 cells were transfected with scrambled control, GSK3 (**D**), or FBXW7 (**E**) siRNA for 48 hours followed by treatment with 500 nM sotorasib for another 24 hours. Proteins in the treated cells were detected by Western blotting. Band intensities were quantified using ImageJ software (NIH) and plotted as a percentage of 0 time point (**B**). (**F**) Schematic illustration of KRAS G12C inhibition-induced GSK3-dependent and FBXW7-mediated degradation of Topo IIα in KRAS G12C–mutant cancer cells. G12Ci, G12C inhibitor.

As determined by RT-qPCR, neither sotorasib nor adagrasib significantly decreased *TOP2A* mRNA expression in H358 or Calu-1 cells (Supplemental Figure 2), suggesting that sotorasib decreases Topo IIα levels via a transcription-independent mechanism. Hence, we determined whether KRAS G12C inhibitors decrease Topo IIα levels through facilitating its degradation, considering that Topo IIα is a protein known to be degraded by a GSK3-dependent and FBXW7-mediated mechanism (9, 10). Both sotorasib and adagrasib decreased the levels of Topo IIα, but not in the presence of MG132, a commonly used proteasome inhibitor, in H358 and Calu-1 cell lines (Figure 2A). In cycloheximide (CHX) chase assays, Topo IIα was degraded much faster in both sotorasib- or adagrasib-treated Calu-1 and H358 cells than in their corresponding DMSO-treated control cells (Figure 2B). These data demonstrate that these KRAS G12C inhibitors induce Topo IIα proteasomal degradation. Following these experiments, we then determined whether KRAS G12C inhibitors promote Topo IIα degradation through a GSK3-dependent and FBXW7-mediated mechanism. Sotorasib decreased Topo IIα levels in both Calu-1 and H358 cells; however, this effect was rescued by the presence of GSK3 inhibitors, including CHIR99021 and SB216763 (Figure 2C), and by GSK3 (Figure 2D) or FBXW7 (Figure 2E) knockdown. Taking these findings together, we conclude that KRAS G12C inhibitors decrease Topo IIα levels through facilitating GSK3-dependent and FBXW7-mediated proteasomal degradation (Figure 2F).

### Modulation of Topo IIα levels in KRAS G12C–mutant cell lines alters cell sensitivity to KRAS G12C inhibitors.

**Figure 3:**
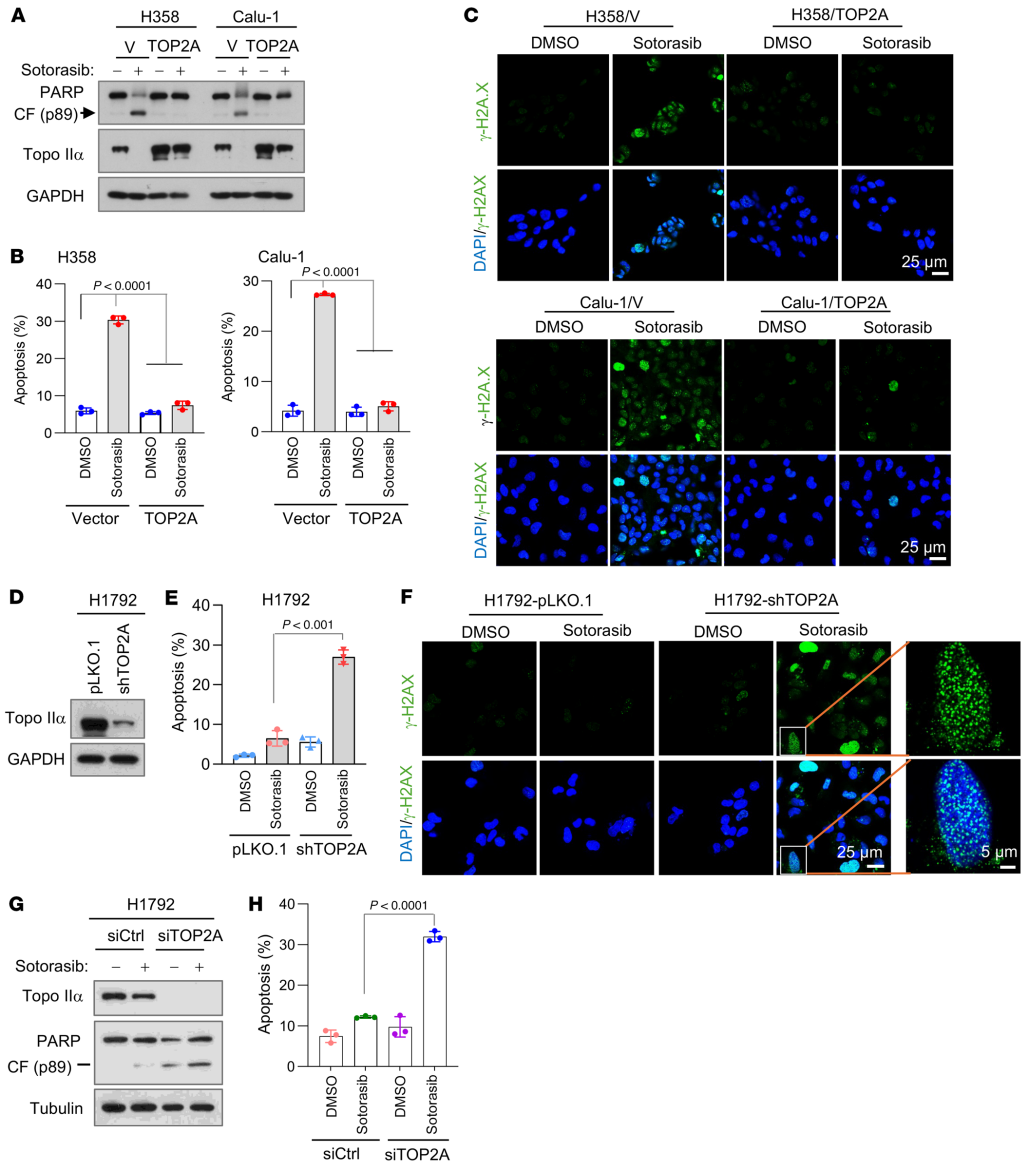
Modulation of Topo IIα levels in KRAS G12C–mutant cell lines alters cell sensitivity to KRAS G12C inhibitors. (**A**–**C**) Both H358 and Calu-1 cell lines expressing vector (V) or *TOP2A* were exposed to DMSO, 200 nM (H358), or 500 nM sotorasib (Calu-1) for 24 hours (**A** and **C**) or 48 hours (**B**). Scale bars: 25 μm. (**D**–**F**) H1792 cells expressing pLKO.1 or shTOP2A (**D**) were treated with 500 nM sotorasib for 24 hours (**F**) or 48 hours (**E**). Scale bars: 25 μm, 5 μm (zoom). (**G** and **H**) H1792 cells were transiently transfected with the given siRNAs for 24 hours followed by the treatment with DMSO or 1 μM sotorasib for 48 hours. The proteins of interest were detected with Western blotting (**A**, **D**, and **G**). Apoptotic cells were detected with annexin V staining/flow cytometry (**B**, **E**, and **H**). Data are shown as the mean ± SD of triplicate treatments (**B**, **E**, and **H**). γ-H2AX foci were detected with IF staining using anti-γ-H2AX antibody (**C** and **F**). Statistical differences between the 2 treatments were determined by 2-sided unpaired Student’s *t* test.

To determine whether Topo IIα reduction is a critical event that mediates therapeutic efficacy of KRAS G12C inhibitors, we enforced expression of the ectopic *TOP2A* gene in sensitive Calu-1 and H359 cells and then examined its impact on sotorasib-induced apoptosis including DNA damage. As presented in Figure 3, we detected Topo IIα reduction accompanied by increased PARP cleavage (Figure 3A) and annexin V–positive apoptotic cells (Figure 3B) in vector control cell lines exposed to sotorasib, but little or no effect in the same cell lines expressing the ectopic *TOP2A* gene. Measurement of cell number alterations also showed that sotorasib had reduced effects on decreasing the survival of the tested cell lines expressing the ectopic *TOP2A* gene compared with its effects in vector control cell lines (Supplemental Figure 3A). In agreement, increased numbers of cells positive for γ-H2AX foci were detected in vector control H358 and Calu-1 cells exposed to sotorasib, but we observed minimal or no increase in the corresponding *TOP2A*-expressing cell lines (Figure 3C). Hence, it is apparent that enforced overexpression of the ectopic *TOP2A* gene in sensitive KRAS G12C–mutant cancer cells abrogates the activity of KRAS G12C inhibitors in inducing DNA damage and apoptosis. Furthermore, we used genetic knockdown to enforce the reduction of *TOP2A* expression in H1792, a KRAS G12C–mutant cell line with limited responses to KRAS G12C inhibitors, to see whether it can sensitize the cells to KRAS G12C inhibitors. shRNA-mediated *TOP2A* knockdown (Figure 3D) significantly enhanced sotorasib’s ability to increase annexin V–positive apoptotic cells (Figure 3E), decrease cell survival (Supplemental Figure 3B), and increase γ-H2AX foci-positive cells in comparison with the control cells infected with vector control (Figure 3F). Similar results were also generated in the same cell line with *TOP2A* siRNA (Figure 3, G and H). Hence, enforced reduction of Topo IIα using genetic knockdown sensitized the insensitive KRAS G12C–mutant cancer cells to sotorasib. These results collectively demonstrate the critical role of Topo IIα reduction or suppression in mediating the therapeutic efficacy of KRAS G12C inhibitors in cancer cell lines with KRAS G12C mutation.

### The presence of Topo II inhibitors enhances therapeutic efficacy of KRAS G12C inhibitors against KRAS G12C–mutant cancer cells/tumors.

**Figure 4:**
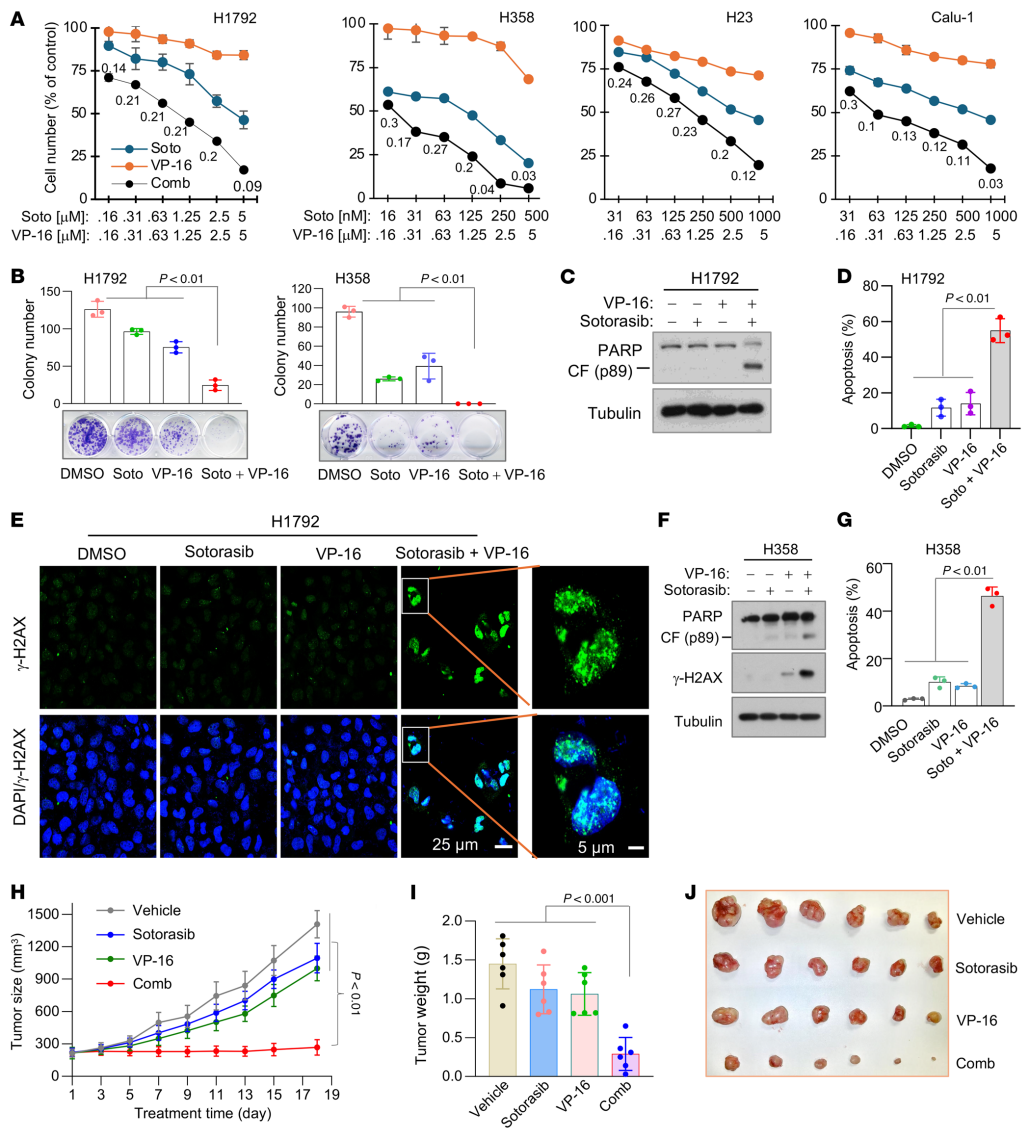
Sotorasib combined with VP-16 synergistically decreases cell survival, augments induction of apoptosis and DNA damage, and enhances suppression of tumor growth in vivo. (**A**) The tested cell lines were treated with varied concentrations of agents either alone or in combinations for 3 days. Cell numbers were measured by SRB assay, and CIs were calculated and presented inside the graph. Data are shown as the mean ± SD of 4 replicate determinations. (**B**) The tested cell lines were treated with 200 nM (H1792) or 20 nM (H358) sotorasib, 500 nM VP-16, or their combination. These treatments were repeated with fresh medium every 3 days. After 10 days, the cells were fixed, stained, and photographed. Data are shown as the mean ± SD of triplicate treatments. (**C**–**E**) H1792 cells were exposed to 1 μM sotorasib, 2.5 μM VP-16, or their combination for 48 hours. γ-H2AX foci were detected with IF staining (**E**). Scale bars: 25 μm, 5 μm (zoom). (**F** and **G**) H358 cells were exposed to 0.1 μM sotorasib, 2.5 μM VP-16, or their combination for 24 hours. The proteins of interest were detected with Western blotting (**C** and **F**). Apoptotic cells were detected with annexin V staining/flow cytometry (**D** and **G**). Data are shown as the mean ± SD of triplicate determinations. (**H**–**J**) H1792 CDXs in *nu*/*nu* nude mice (*n* = 6/group) were treated with vehicle, sotorasib alone (50 mg/kg, daily, og), VP-16 alone (1 mg/kg, daily, i.p.), or their combination. Tumor sizes (**H**) were measured at the indicated time points. At the end of treatment, tumors in each group were weighed (**I**) and photographed (**J**). Data are shown as the mean ± SEM of 6 tumors from 6 mice. Statistical analysis was conducted using 1-way ANOVA.

Given the limited activity of current KRAS G12C inhibitors in cell culture, as demonstrated above and in patients (1, 3), we then wondered whether inclusion of a Topo II inhibitor such as VP-16 or DXR in RAS-targeted therapy could boost the therapeutic efficacy of RAS-targeted cancer therapy. To this end, we tested the effects of sotorasib in combination with VP-16 on the survival of several NSCLC cell lines with KRAS G12C mutation, particularly H1792 with low sensitivity to KRAS G12C inhibitors. The combination of sotorasib and VP-16 was much more potent than either agent alone in decreasing the survival of the tested cell lines with combination indexes (CIs) of <1 (Figure 4A), indicating synergistic effects on decreasing the survival of KRAS G12C–mutant NSCLC cells. Using H1792 as a representative cell line with primary resistance to KRAS G12C inhibitors, we also tested the combination of sotorasib and DXR or the combination of adagrasib with VP-16 or DXR and generated similar results (Supplemental Figure 4, A and B). Importantly, these combinations did not show enhanced effects on decreasing the survival of other NSCLC cell lines without KRAS G12C mutation (Supplemental Figure 4C). Topo I inhibitors represent another group of widely used chemotherapeutic drugs in the clinic. We were curious to see whether the combination of a KRAS G12C inhibitor and a Topo I inhibitor exerted enhanced effects on decreasing the survival of KRAS G12C–mutant cancer cells. The results showed that sotorasib combined with either irinotecan or topotecan did not have augmented effects on decreasing the survival of either Calu-1 or H358 cells (Supplemental Figure 4D). Similarly, sotorasib combined with carboplatin, cisplatin, or paclitaxel, which are commonly used chemotherapeutic agents in the clinic without Topo II inhibitory activity, did not show enhanced effects on decreasing the survival of both Calu-1 and H358 cell lines (Supplemental Figure 4E). These results together demonstrate the critical role of Topo II inhibition in augmenting therapeutic efficacy of KRAS G12C inhibitors against KRAS G12C–mutant cancer cells.

Consistently, the combination of sotorasib and VP-16 was significantly more effective than either single agent in suppressing colony formation/growth of the tested H1792 and H358 cells (Figure 4B). Moreover, the combination was also more active than either single agent in inducing apoptosis, as evidenced by increased PARP cleavage (Figure 4C) and annexin V–positive cells (Figure 4D) and increased γ-H2AX foci-positive cells (Figure 4E) in H1792 cells. Consistent results were also generated in H358 cells (Figure 4, F and G). Clearly, the combination of a KRAS G12 inhibitor and a Topo II inhibitor enhances therapeutic efficacy against KRAS G12C–mutant cancer cells.

Since Bim induction and Mcl-1 reduction are critical events that mediate the enhanced induction of apoptosis by osimertinib combined with VP-16 in EGFR-mutant NSCLC cell lines, as demonstrated previously (9), we were next interested in knowing whether sotorasib and VP-16 enhance apoptosis in KRAS G12C–mutant cell lines through similar mechanisms. We found that the combination of sotorasib and VP-16 elevated Bim levels, while either agent alone did not do so in H1792 cells. This combination did not further decrease Mcl-1 levels (Supplemental Figure 5A). In H1792 cells transfected with Bim siRNA, which silences Bim expression or elevation, the combination of sotorasib and VP-16 lost its ability to enhance PARP cleavage (Supplemental Figure 5B) and annexin V–positive cells (Supplemental Figure 5C), indicating the attenuated induction of apoptosis. Hence, it appears that Bim induction plays a critical role in mediating enhanced induction of apoptosis by the combination of sotorasib and VP-16 in KRAS G12C–mutant cancer cells.

We then tested the efficacy of the combination on the growth of H1792 cell–derived xenografts (CDXs) in nude mice. In agreement with the in vitro data presented above, the combination of sotorasib and VP-16 was significantly more effective than each drug alone in suppressing the growth of H1792 CDXs, which were insensitive to sotorasib in vivo as well, as evaluated by measuring tumor sizes and weights (Figure 4, H–J). IHC analysis of the tumor tissues showed that the combination had the most potent effects on decreasing Ki67 staining and on increasing the staining of cleaved PARP (cPARP; an apoptotic marker) and Bim (Supplemental Figure 6A). The combination did not alter mouse body weights in comparison with other groups (Supplemental Figure 6B), indicating favorable tolerability in vivo. Thus, these results confirm the efficacy of the combination against KRAS G12C–mutant tumors in vivo with enhanced induction of apoptosis and suppression of cell proliferation.

Another interesting observation is that the combination of sotorasib and VP-16 significantly decreased the levels of Topo IIα in H1792 CDX tumors, while either single agent had little or no effect (Supplemental Figure 6, C and D), albeit with unclear mechanisms.

### The combination of sotorasib and VP-16 effectively eliminates DTPs with elevated Topo IIα and delays the emergence of acquired resistance to sotorasib in vivo.

**Figure 5:**
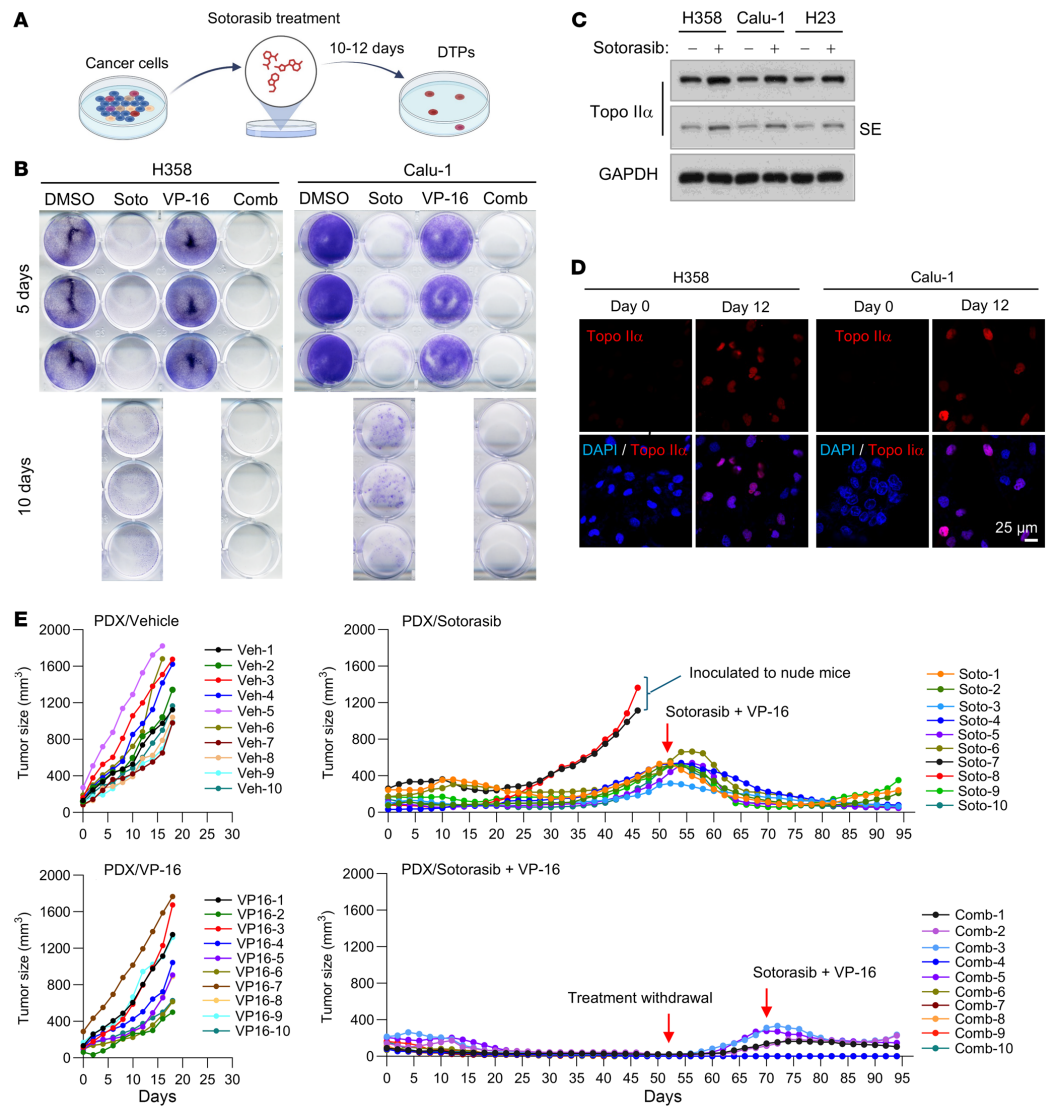
The combination of sotorasib and VP-16 effectively eliminates DTPs that possess elevated levels of Topo IIα and delays the emergence of acquired resistance to sotorasib in vivo. (**A**) Schematic procedure for the DTP assay. (**B**) H358 and Calu-1 cells seeded in 12-well plates were treated with 80 nM (H358) or 1,000 nM (Calu-1) sotorasib, 500 nM VP-16, or their combination. The same treatments were repeated with fresh medium every 3 days. After 5 or 10 days, the cells were stained with crystal violet dye, photographed, and counted. (**C** and **D**) The indicated cell lines were treated with 100 nM (H358) or 1,000 nM (Calu-1 and H23) sotorasib. The cells were refreshed every 3 days with fresh medium containing the same treatment. After 12 days, cells were either harvested (**C**) for Western blotting or reseeded onto slides for IF (**D**). SE, short exposure. Scale bar: 25 μm. (**E**) PDX tumors harboring KRAS G12C mutation in nude mice (10 tumors/group from 5 mice) were treated with vehicle, sotorasib alone (50 mg/kg, daily, og), VP-16 alone (1 mg/kg, daily, i.p.), or their combination. Tumor sizes were measured at the indicated time points. Data are shown as the mean ± SEM of 10 tumors.

It is recognized that DTPs surviving from the initial treatment with a given targeted therapy is an important mechanism underlying the development of acquired resistance to targeted therapies (11, 12). We therefore analyzed the effects of the combination on eliminating DTPs of RAS G12C–mutant NSCLC cell lines, as depicted in Figure 5A. In a 10-day DTP assay, we detected surviving DTPs in both Calu-1 and H358 cells exposed to sotorasib alone, but not in the same cells treated with the combination of sotorasib and VP-16 (Figure 5B), demonstrating the potent efficacy of the combination in eliminating DTPs in vitro. We further determined whether the effectiveness of the combination against DTPs is due to the high levels of Topo IIα in the DTPs. Using Western blotting, we detected higher levels of Topo IIα in DTPs of H358, Calu-1, and H23 cells surviving from sotorasib treatment for 12 days than those in their corresponding control cells (Figure 5C). We also stained Topo IIα in DTPs surviving from sotorasib treatment with IF and found that the intensity of Topo IIα staining in the DTPs was much higher than in the control cells (Figure 5D). These results together indicate increased Topo IIα levels in DTPs.

Following this study, we further evaluated the effect of the combination on inhibiting tumor growth and on delaying emergence of acquired resistance to sotorasib using a NSCLC patient–derived xenograft (PDX) model harboring KRAS G12C mutation given the augmented effects of the combination on overcoming primary sotorasib resistance and eliminating DTPs, as demonstrated above. While VP-16 at the tested low dosage had limited effect on suppressing the growth of PDXs, sotorasib and particularly the combination of sotorasib and VP-16 effectively repressed the growth of the PDXs in the initial treatment period (<25 days). As the treatment continued, PDXs in mice receiving sotorasib remained the same sizes or started to grow larger; however, PDXs in mice treated with the combination regressed completely after day 25. Except for 2 PDXs in the sotorasib group that quickly grew to sizes of over 500 mm^3^ after 32 days, all other PDXs receiving sotorasib treatment began to grow after 40 days and reached over 500 mm^3^ after 52 days. The PDXs treated with the combination remained in complete regression on day 52. On day 52, the combination treatment was stopped to see whether these undetectable tumors would resume growth. We observed that 4 of 10 tumors started to resume growth after day 60, whereas the remaining tumors (6/10 tumors) remained in complete regression until the end of the study on day 94 (Figure 5E and Supplemental Figure 7A). When the 4 tumors with regrowth reached sizes of around 300 mm^3^, they were exposed again to the combination of sotorasib and VP-16; the tumors regressed partially, although not to the point of complete regression by the time of study end (Figure 5E and Supplemental Figure 7A). On day 52, the tumors receiving sotorasib monotherapy were switched to treatment with sotorasib plus VP-16. The tumors then responded to the combination and began to shrink after around 10 days of treatment. On day 82, all tumors had shrunk to sizes of around 100 mm^3^. Unfortunately, some tumors started to enlarge as the treatment continued (Figure 5E and Supplemental Figure 7A), indicating the emergence of acquired resistance to the combination of sotorasib and VP-16. Encouragingly, the body weights of mice receiving daily administration of the combination of sotorasib and VP-16 for a long period of 94 days were not reduced compared with those of mice treated with sotorasib alone (Supplemental Figure 7B), indicating the favorable tolerability of the combination in mice.

### KRAS G12C–mutant cell lines with acquired resistance to KRAS G12C inhibitors have elevated levels of Topo IIα due to increased stability and are sensitive to cotargeting of RAS and Topo II.

**Figure 6:**
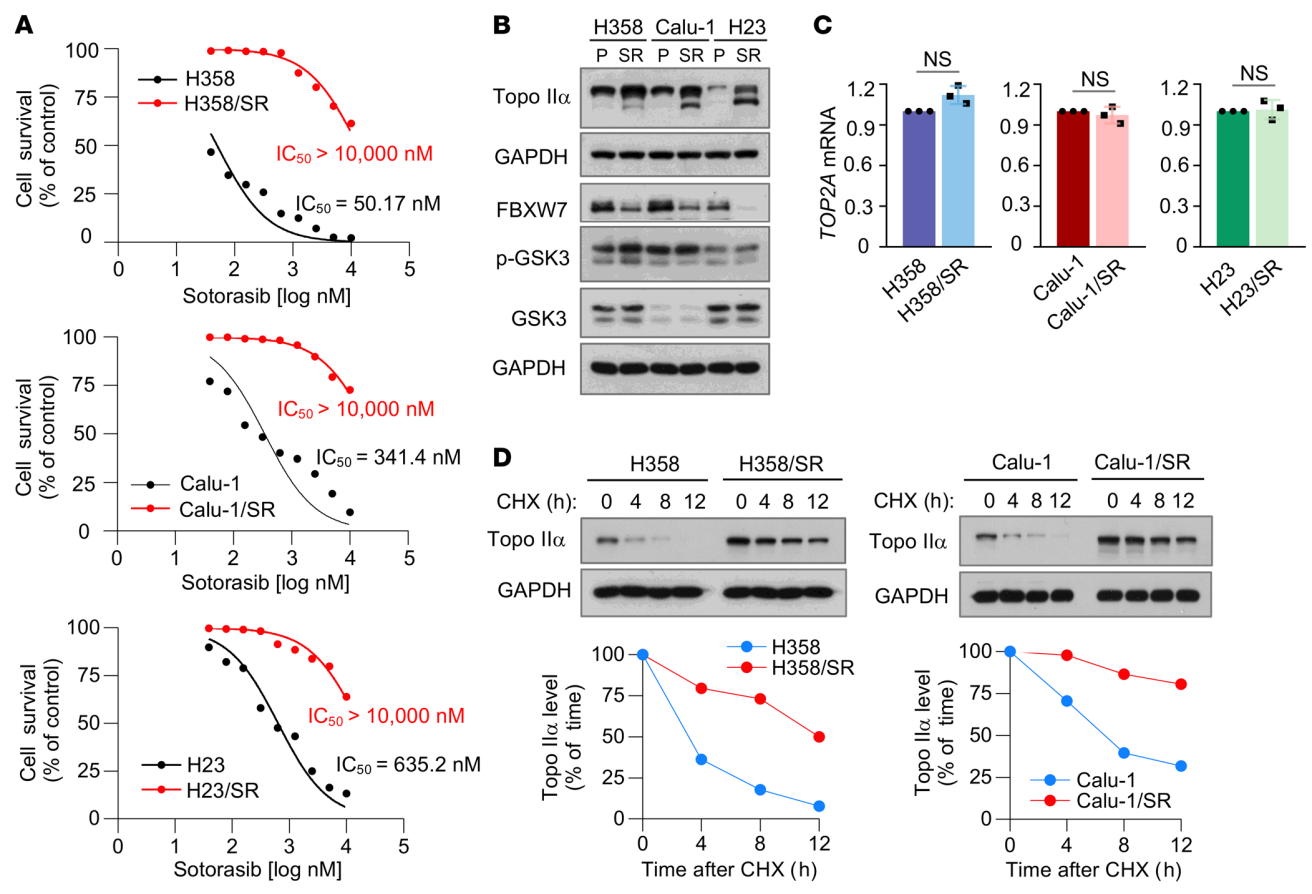
KRAS G12C mutant cell lines with acquired resistance to KRAS G12C inhibitors have elevated levels of Topo IIα due to increased stability. (**A**) The given cell lines were exposed to different concentrations of sotorasib for 72 hours. Cell numbers were then measured by the SRB assay. Data are shown as the mean ± SD of 4 replicate determinations. (**B**) Western blotting was used to detect the indicated proteins in H358, Calu-1, H23, and their corresponding sotorasib-resistant cell lines. P, parental; SR, sotorasib-resistant. (**C**) Baseline *TOP2A* mRNA expression in the indicated cell lines was detected with RT-qPCR. Significance was determined by 2-sided unpaired Student’s *t* test. (**D**) The tested cell lines were treated with 10 μg/mL CHX and then harvested at different times as indicated. The proteins of interest were detected with Western blotting. Band intensities were quantified using ImageJ software and plotted as a percentage of the 0 time point.

Acquired resistance to targeted cancer therapy, including RAS-targeted cancer therapy, is a challenging issue in the clinic that leads to eventual treatment failure, limiting its long-term therapeutic benefit to patients. To determine whether the cotargeting of RAS and Topo II is a valid strategy for overcoming acquired resistance to RAS-targeted cancer therapy, we first established 3 KRAS G12C–mutant NSCLC cell lines with acquired resistance to sotorasib (named SR) through gradually increasing the concentration of sotorasib, as presented in Figure 6A. Analysis of these SR cell lines in comparison with their corresponding parental cell lines using whole-genome sequencing (WGS) did not identify any new *KRAS* mutations except for the known G12C mutation. Other gene alterations detected did not show any difference between SR and their parental cell lines (Supplemental Figure 8). However, these SR cell lines possessed elevated basal levels of Topo IIα compared with their corresponding parental cell lines (Figure 6B). Moreover, these resistant cell lines had lower levels of FBXW7 and slightly higher levels of p-GSK3 (inactive) in comparison with their corresponding parental cell lines (Figure 6B), suggesting the possibility that the resistant cell lines possess stabilized Topo IIα. This is supported by our finding that *TOP2A* mRNA expression was not significantly different between parental and corresponding SR cell lines (Figure 6C). Thus, we further checked the stability of Topo IIα in these resistant cell lines and found that Topo IIα was indeed degraded much more slowly in the resistant cell lines than in their corresponding parental cell lines (Figure 6D), confirming the increased stability of Topo IIα in sotorasib-resistant cancer cell lines.

**Figure 7:**
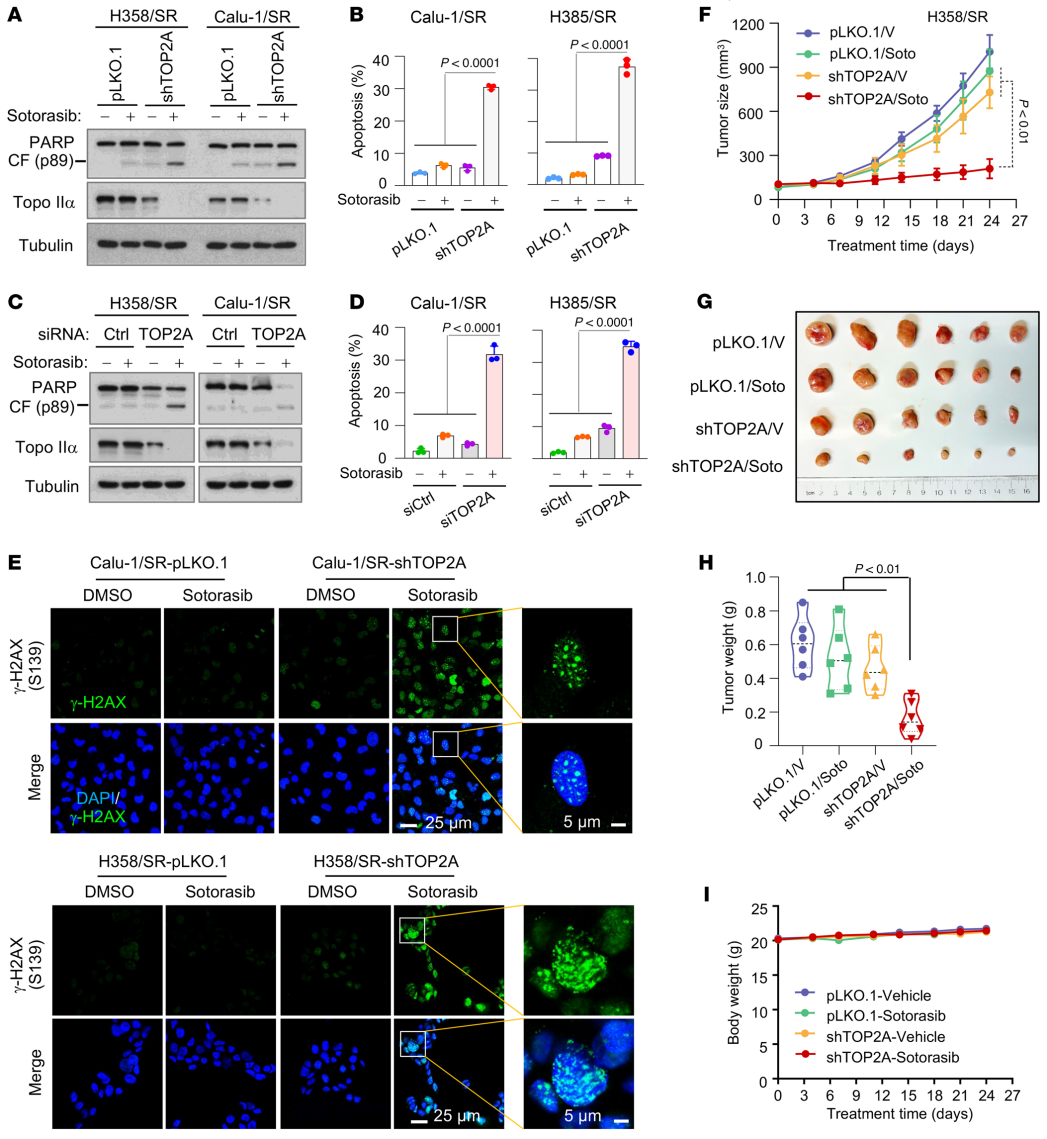
Genetic knockdown of *TOP2A* expression in sotorasib-resistant NSCLC cell lines sensitizes the cells to sotorasib. (**A**–**D**) Both H358/SR and Calu-1/SR cells expressing pLKO.1 or shTOP2A (**A** and **B**) or transfected with scrambled control or *TOP2A* siRNA for 48 hours (**C** and **D**) were exposed to DMSO or 10 μM sotorasib for 48 hours. The levels of Topo IIα and cleavage of PARP were detected with Western blotting (**A** and **C**). Annexin V–positive cells were determined with flow cytometry (**B** and **D**). Data are shown as the mean ± SD of triplicate determinations. (**E**) The indicated cell lines described above were exposed to DMSO or 10 μM sotorasib for 48 hours and then IF conducted to stain γ-H2AX foci. Scale bars: 25 μm, 5 μm (zoom). (**F**–**I**) Mice with the indicated tumors derived from H358/SR cells were treated with vehicle (V) or sotorasib (50 mg/kg, daily, og) for 24 days. Tumor sizes (**F**) and mouse body weights (**I**) were measured at the indicated time points, and at the end of the treatment, collected tumors were photographed (**G**) and weighed (**H**). Data are shown as the mean ± SEM of 6 tumors from 6 mice. Statistical differences among the treatments were determined by 1-way ANOVA.

We next determined the impact of genetic inhibition of Topo IIα on the responses of the sotorasib-resistant cell lines to sotorasib. We detected significantly increased PARP cleavage (Figure 7A) and annexin V–positive cells (Figure 7B) in both H358/SR and Calu-1/SR cell lines expressing *TOP2A* shRNA (shTOP2A), but minimal or no increase in their corresponding control cell lines, indicating the enhanced induction of apoptosis in the *TOP2A*-knockdown sotorasib-resistant cell lines. A cell survival assay also showed that the *TOP2A*-knockdown sotorasib-resistant cell lines were more sensitive than the control cell lines to sotorasib (Supplemental Figure 9A). We noted that sotorasib treatment further decreased the levels of Topo IIα in the tested resistant cell lines in which Topo IIα levels were already reduced (Figure 7A). Similar results were also generated in the same cell lines transfected with *TOP2A* siRNA (Figure 7, C and D). Consistently, increased numbers of cells positive for γ-H2AX foci were detected in both sotorasib-treated H358/SR and Calu-1/SR cell lines expressing shTOP2A or TOP2A siRNA, but not in their corresponding control cells exposed to sotorasib (Figure 7E and Supplemental Figure 9B). These results clearly indicate that *TOP2A* knockdown in sotorasib-resistant cell lines sensitizes the cells to undergo sotorasib-induced DNA damage.

We further inoculated H358/SR cells expressing shTOP2A into nude mice and treated them with sotorasib. As observed in vitro, sotorasib was ineffective in inhibiting the growth of H358/SR-pLKO.1 tumors but significantly inhibited the growth of H358/SR-shTOP2A tumors, as evaluated by tumor sizes (Figure 7, F and G) and weights (Figure 7H). Mouse body weights did not show apparent differences among the groups (Figure 7I), indicating that inhibition of *TOP2A* expression with shTOP2A does not increase the toxicity of sotorasib.

### KRAS G12C–mutant cell lines and tumors with acquired resistance to KRAS G12C inhibitors are sensitive to the combination of a KRAS G12C inhibitor and a Topo II inhibitor, which is well tolerated in mice.

**Figure 8:**
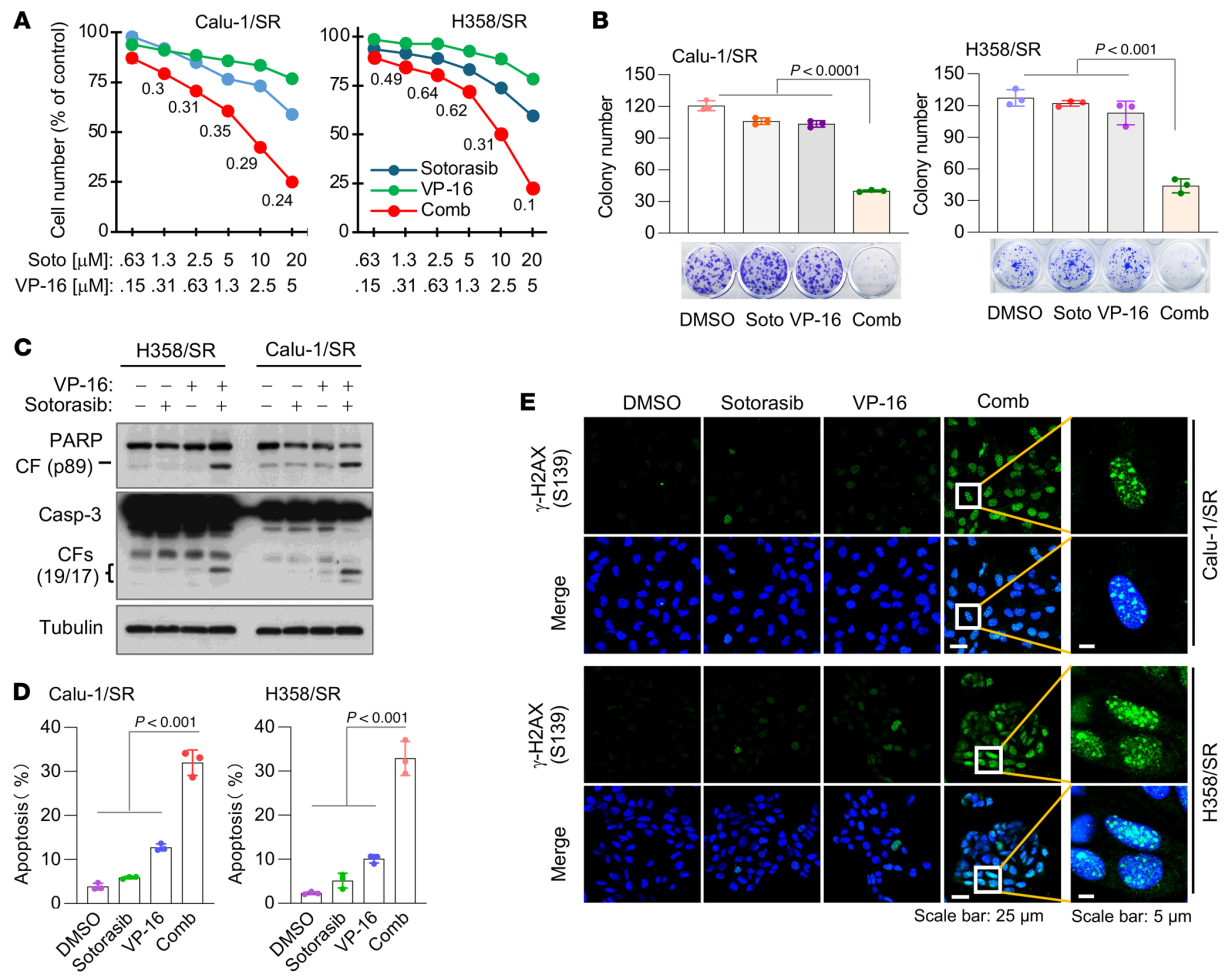
KRAS G12C–mutant cell lines with acquired resistance to KRAS G12C inhibitors are sensitive to the combination of a KRAS G12C inhibitor and VP-16 in decreasing cell survival and inducing apoptosis and DNA damage. (**A**) H358/SR and Calu-1/SR cells in 96-well plates were exposed to varying concentrations of the tested agents either alone or in combination for 3 days. Cell viability was then assessed using the SRB assay, and CIs were calculated. Data are shown as the mean ± SD of 4 independent replicates. (**B**) Calu-1/SR and H358/SR cells were treated with 500 nM (Calu-1) or 100 nM (H358) sotorasib, 500 nM VP-16, or their combination. The cells were refed with fresh medium containing the same treatment every 3 days. After 10 days, cells were stained with crystal violet dye, photographed, and counted. Data are shown as the mean ± SD of triplicate determinations. (**C**–**E**) Both H358/SR and Calu-1/SR cells were treated with DMSO, 10 μM sotorasib, 2.5 μM VP-16, or their combination for 48 hours. The proteins of interest were detected with Western blotting (**C**). Apoptotic cells were detected with annexin V staining/flow cytometry (**D**). γ-H2AX foci were detected with IF staining (**E**). Scale bars: 25 μm, 5 μm (zoom). Data are shown as the mean ± SD of triplicate determinations. Statistical analysis was conducted using 1-way ANOVA.

The above findings clearly demonstrate that genetic inhibition of Topo IIα in sotorasib-resistant cell lines restores cell sensitivity to sotorasib. We thus determined the effect of sotorasib combined with VP-16 or DXR on the growth of sotorasib-resistant cell lines. In a similar fashion, the combination of sotorasib and VP-16 was more active than either agent alone in decreasing cell survival, with CIs of <1 (Figure 8A), suppressing colony formation and growth (Figure 8B), enhancing PARP and caspase-3 cleavage (Figure 8C), and increasing annexin V–positive cells (Figure 8D) in both H358/SR and Calu-1/SR cell lines. In agreement, the combination increased the number of cells positive for γ-H2AX foci in both Calu-1/SR and H358/SR cell lines, whereas each drug alone had minimal or no effect on increasing γ-H2AX foci-positive cells (Figure 8E). Similar results were also generated in the resistant cell lines exposed to the combination of sotorasib with DXR (Supplemental Figure 10). Hence, these combinations synergistically inhibit the growth of sotorasib-resistant KRAS G12C–mutant cells with enhanced induction of DNA damage and apoptosis.

**Figure 9:**
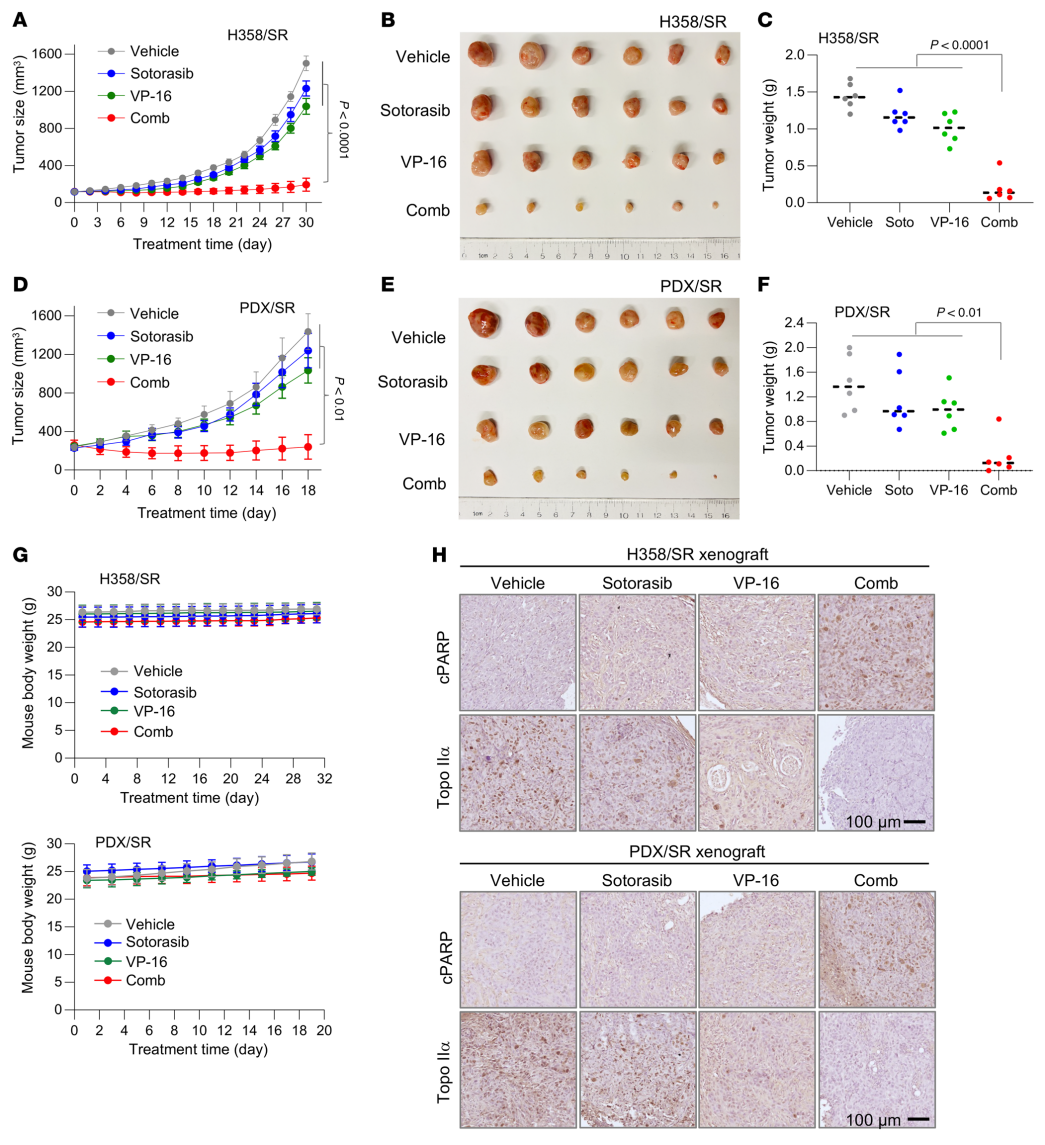
KRAS G12C mutant tumors with acquired resistance to KRAS G12C inhibitors are sensitive to the combination of a KRAS G12C inhibitor and VP-16 in vivo. (**A**–**G**) H358/SR cells and PDX tumors developing acquired sotorasib resistance, as presented in Figure 5E, were injected into nude mice to establish H358/SR CDXs (**A**–**C**) and PDX/SR (**D**–**F**) tumor models, respectively. Both tumor models were treated continuously with vehicle, sotorasib alone (50 mg/kg, daily, og), VP-16 alone (1 mg/kg, daily, i.p.), or their combination. Tumor sizes (**A** and **D**) and mouse body weights (**G**) were measured at the indicated time points. At the end of the treatment, collected tumors were photographed (**B** and **E**) and weighed (**C** and **F**). (**H**) Tumor tissues slides were stained for cPARP and Topo IIα using IHC. Scale bars: 100 μm. Data are shown as the mean ± SEM of 6 tumors from 6 mice. Statistical analysis was conducted using 1-way ANOVA.

Following these in vitro studies, we determined the effects of the combination on the growth of sotorasib-resistant tumors in mice. While both sotorasib and VP16 alone under the tested conditions had limited effects on suppressing the growth of H358/SR tumors, the combination of sotorasib and VP-16 effectively inhibited tumor growth and was significantly more potent than each single agent alone, as evaluated by both tumor sizes (Figure 9, A and B) and weights (Figure 9C). In the subsequent study with PDX/SR tumors, we waited until tumor sizes reached around 300 mm^3^ and then started treatments. Consistently, we observed that the combination of sotorasib and VP-16 was much more potent than each drug alone in inhibiting the growth of PDX/SR tumors (Figure 9, D–F). In both studies, the body weights of mice receiving the combination treatment were not different from those of mice in other groups (Figure 9G), indicating favorable tolerability in mice. We performed IHC and detected the highest positive staining for cPARP in tumor tissues of mice receiving the treatment of sotorasib combined with VP-16, in which Topo IIα staining was reduced in comparison with that in tumor tissues from other treatment groups (Figure 9H). These results indicate that the combination of sotorasib and VP-16 enhances the reduction of Topo IIα levels and induction of apoptosis in vivo.

We conducted the same treatments in immunocompetent mice to further examine the potential toxicities of the sotorasib and VP-16 combination. After about 4 weeks of treatment, mouse body weights in the combination group were comparable with those in the single-agent treatment groups (Supplemental Figure 11A). Histological examination of tissues from the major organs, including lung, heart, kidney, spleen, and liver, among the different groups did not show differences (Supplemental Figure 11B). The detection of various serum protein markers or enzymatic activities that reflect the functions of organs, the immune/inflammatory system, the erythrocyte/oxygen transport system, and the platelet/coagulation system among the tested groups did not show significant differences either (Supplemental Figure 12). Hence, the combination of sotorasib and VP-16 is well tolerated without apparent toxicities in the immunocompetent mice as well.

### Baseline and pretreatment levels of Topo IIα predict therapeutic outcomes of KRAS G12C inhibitors in the treatment of patients with RAS G12C–mutant NSCLC.

**Figure 10:**
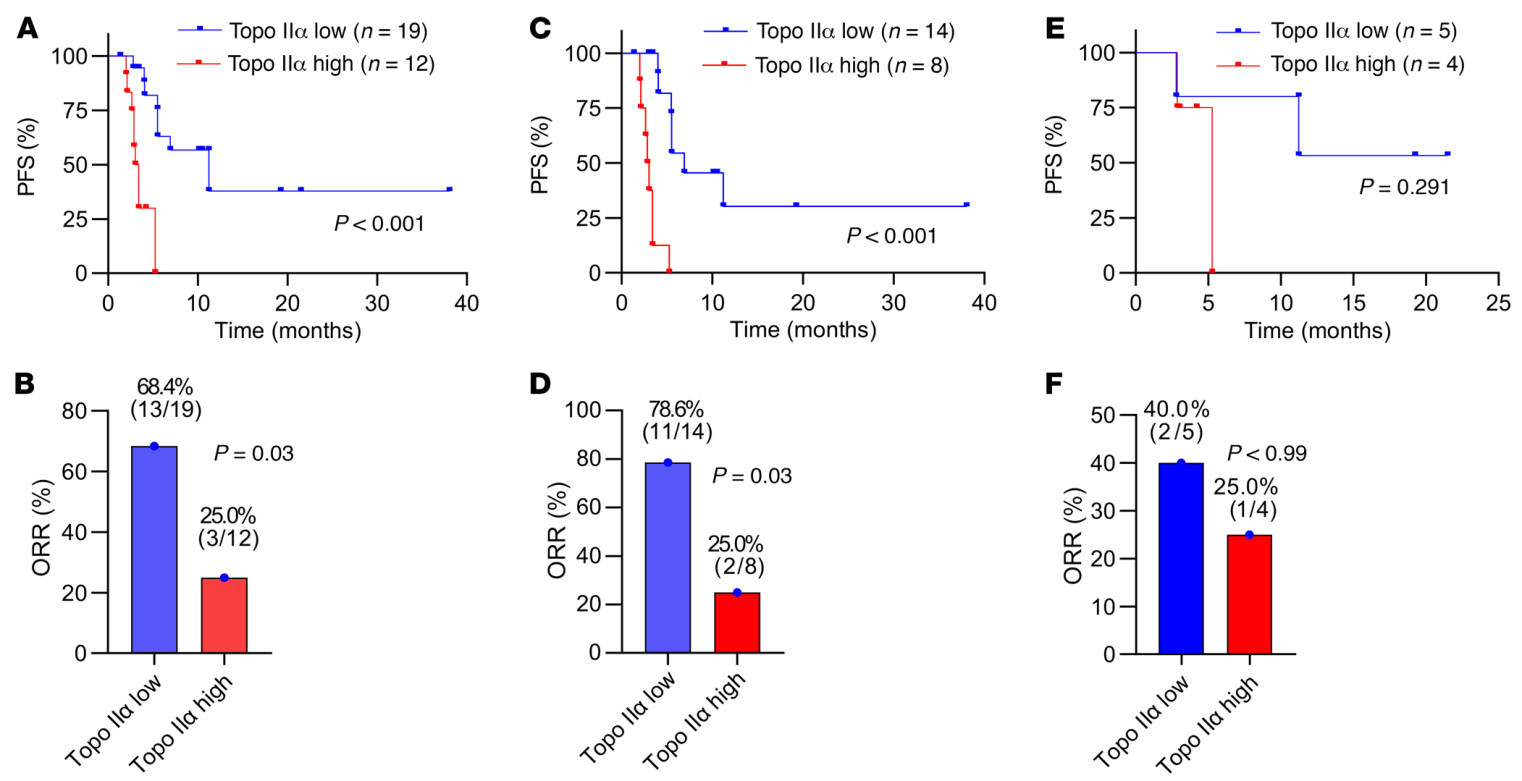
Topo IIα protein expression correlates with PFS and ORR in the overall cohort samples and pretreatment samples. (**A**, **C**, and **E**) Kaplan-Meier curves for PFS by Topo IIα expression status in the overall cohort samples including baseline and pretreatment ones (**A**), in the pretreatment samples (**C**), and in baseline samples (**E**). (**B**, **D**, and **F**) ORRs between Topo IIα low and high groups in the overall cohort (**A**), pretreatment (**D**), and baseline (**F**) samples. Statistical differences were determined by 2-sided unpaired Student’s *t* test (PFS) and Fisher’s exact test (ORR).

Thirty-one male patients with lung adenocarcinoma harboring KRAS G12C mutation were included in the study, and their clinical characteristics are summarized in Supplemental Table 1. Among these tissues, there were 9 baseline tissues (at the time of diagnosis) and 22 pretreatment tissues (before the initiation of treatment with the KRAS G12C inhibitor fulzerasib). IHC staining results for Topo IIα protein expression in these tissues were scored as weighted index (WI; intensity 0–3 × % positive staining). WI of 10 was then chosen to define the expression levels of Topo IIα as low (WI < 10) or high (WI ≥ 10). The median follow-up duration was 13.1 (4.3–22.1) months. By the end of follow-up, 61.3% (19/31) of patients had disease progression. Interestingly, we found that patients with low Topo IIα expression in tumors had significantly longer median PFS (mPFS) than those with high Topo IIα expression (11.2 vs. 3.2 months; *P* < 0.001; Figure 10A) using all the samples in the cohort with PFS follow-up information. ORR was also significantly higher in the low expression group than in the high expression group (68.4% [13/19] vs. 25.0% [3/12], *P* = 0.029; Figure 10B). Given that RAS G12C inhibitors were approved for a late line of therapy, we further analyzed Topo IIα levels in the subset of 22 cases with recent pretreatment tissues, in which 68.2% (15/22) had disease progression. Consistent with the results generated from the overall cohort samples, patients with KRAS G12C–mutant NSCLC expressing low levels of Topo IIα had significantly longer mPFS (6.9 vs. 3.0 months, *P* < 0.001; Figure 10C) and higher ORR (78.6% [11/14] vs. 25.0% [2/8], *P* = 0.026; Figure 10D) in comparison with those with tumors expressing high levels of Topo IIα. Among the 9 patients with baseline tumor samples, 44.4% (3/9) had disease progression. Although the difference did not reach statistical significance due to the small sample size, low Topo IIα expression trended toward longer mPFS (not reached vs. 5.3 months, *P* = 0.291; Figure 10E) and higher ORR (40.0% [2/5] vs. 25.0% [1/4], *P* > 0.999; Figure 10F). Hence, it appears that both baseline and pretreatment levels of Topo IIα determine therapeutic outcomes of KRAS G12C inhibitors in the treatment of patients with KRAS G12C mutation.

## Discussion

The intriguing findings in this study are the connection between suppression of Topo IIα and therapeutic efficacies of KRAS G12C inhibitors and the enhanced therapeutic efficacy when combining a KRAS G12 inhibitor with a Topo II inhibitor such as VP-16. In this study, all results from cell lines were generated with clinically achievable concentration ranges (≤1 μM) of sotorasib and adagrasib, which are actually below around 13 μM (960 mg daily dose) and 1.63 μM (600 mg twice a day) of maximum plasma concentrations, respectively, in humans (13–15), demonstrating clinical relevance.

Beyond KRAS G12C inhibitors, various approaches are currently being explored to target other KRAS mutations, such as G12D, with either mutation-selective inhibitors or pan inhibitors, as potential RAS-targeted therapies. The current study primarily used KRAS G12C inhibitors and NSCLC cell lines with KRAS G12C mutation as models to demonstrate our appealing concept of enhancing RAS-targeted cancer therapy. Given the shared signaling pathways downstream of RAS, this mechanism, i.e., Topo IIα suppression and DNA damage induction as a critical event that mediates therapeutic efficacy of KRAS G12C–targeted therapy, and potential strategy for enhancing KRAS G12C–targeted therapy through targeting Topo II could be logically applied to other RAS-targeted cancer therapies irrespective of cancer type.

Mechanistic studies have demonstrated that KRAS G12 inhibitors induce GSK3-dependent, FBXW7-mediated proteasomal degradation of Topo IIα, leading to DNA damage and subsequent apoptosis in KRAS G12–mutant NSCLC cells. This is in line with the mechanism by which the third-generation EGFR inhibitor, osimertinib, induces Topo IIα degradation, DNA damage, and apoptosis in EGFR-mutant NSCLC cells, as we demonstrated previously (9). This is no surprise because RAS functions immediately downstream of EGFR to mediate activation of PI3K/Akt and Raf/MEK/ERK oncogenic signaling pathways. Compared with EGFR mutations that primarily occur in NSCLC, RAS, and particularly KRAS, mutations occur frequently across different cancer types, such as lung, pancreatic, and colorectal cancer. Therefore, the finding of cotargeting Topo II to enhance RAS-targeted therapy should have a broader impact on clinical treatment of cancer compared with similar strategies for enhancing EGFR-targeted therapy in lung cancer.

Compared with EGFR-targeted therapy, current KRAS G12C inhibitors have much shorter ORR and mPFS (1, 4, 5). The finding of Topo IIα expression as a potential biomarker for predicting responses of patients with KRAS G12C–mutant NSCLC to KRAS G12C inhibitors is of high translational significance in the clinic. Combining the detection of KRAS G12C mutation and Topo IIα expression in either baseline or pretreatment samples allows us in a more precise way to identify patients who are likely to respond better to KRAS G12C inhibitor monotherapy. Thus, further validation of RAS mutation plus Topo IIα as a predictive strategy for guiding RAS-targeted cancer therapy is warranted. Due to the limited numbers of baseline samples, detection of Topo IIα in these samples did not reach statistical significance, albeit with similar trends in both ORR and mPFS in this study. However, the findings warrant further validation in this direction.

Platinum-based chemotherapy in combination with immunotherapy is a standard of care for patients with NSCLC, particularly in the scenario without the option for a targeted therapy or after failure of immunotherapy, if not used in combination in first-line therapy. The recently approved regimen of the combination of osimertinib with platinum-based chemotherapy prolongs PFS 9 months longer than osimertinib monotherapy and has become standard first-line therapy for patients with NSCLC harboring activating EGFR mutations (16, 17), indicating the impact of chemotherapy on targeted cancer therapy. The Topo II inhibitors VP-16 and DXR are well-known chemotherapeutic drugs that have long been used for the treatment of cancers, including lung cancer, albeit less frequently (6, 18). The current study has demonstrated the scientific rationale for enhancing RAS-targeted cancer therapy via targeting Topo II. Platin drugs such as carboplatin and cisplatin, Topo I inhibitors such as irinotecan and topotecan, and antimicrotubule drugs such as paclitaxel are widely used chemotherapeutic agents in the treatment of cancer, including NSCLC. However, these agents failed to show any enhanced effects when combined with sotorasib against the growth of KRAS G12C–mutant NSCLC cells, as demonstrated in our study, further highlighting the importance of Topo II inhibition in this strategy. In our animal studies, the combination of VP-16 with sotorasib exhibited promising activity either used as a first-line therapy for the treatment of KRAS G12C–mutant tumors with primary resistance to KRAS G12C inhibitors (e.g., H1792 CDXs) and for delaying or even preventing the emergence of acquired resistance to sotorasib in KRAS G12C–mutant PDXs or used as a second line option for the treatment of tumors (both CDXs and PDXs) with acquired resistance to sotorasib. Moreover, the combination was well tolerated in mice even after a long period of daily administration over 3 months without apparent body weight reduction. Hence, our findings reported in this study warrant the clinical validation of this intriguing therapeutic strategy.

In the resistance delay study, most mice (60%; 6/10) were tumor-free after treatment withdrawal for over 40 days, reaching a point of cure in this preclinical model. Given that the current KRAS G12C inhibitors as a monotherapy in general have relatively lower overall response rates and shorter response durations or limited impact on patient survival compared with those of other targeted therapies, primarily due to primary resistance or early emergence of acquired resistance (1, 3), our findings are thus of high clinical significance and provide a solid foundation or rationale in support of early application of RAS-targeted therapy in combination with a Topo II inhibitor such as VP-16 as a first line therapy. One advantage is that VP-16 can be administered orally in comparison with many other chemotherapeutic agents that have to be administered via injection. This feature is important considering the need for a long period of combined treatment in some patients if used as a first-line therapy. The oral dosage of VP-16 at 50 mg is typically used in combination with other chemotherapeutic agents in humans and is equivalent to a dosage of around 10 mg/kg in mice. The dosage of VP-16 used in our animal studies was 1 mg/kg (i.p.). It is known that the oral bioavailability of VP-16 in humans is about 50% of the administered dose by injection (19). Hence, we can use an even lower dose of VP-16, such as 25 mg, in humans, which is equivalent to about 5 mg/kg in mice, in the combination to boost the efficacy of RAS-targeted therapy without increasing potential toxicity.

In our therapeutic studies using models with acquired sotorasib resistance, the combination of sotorasib and VP-16 could not reach the point of eliminating the tumors, albeit with high potency in shrinking and retarding the growth of tumors. The resistant PDX tumors in the delay experiment even regrew after switching to the combinatorial treatment and responding for a while, indicating a possible development of acquired resistance to the combinatorial treatment. Thus, these findings suggest that the combination should be used as an early line of therapy in the KRAS G12C treatment-naive setting to substantially prolong the duration of response and even prevent the emergence of acquired resistance. The molecular mechanisms underlying the emergence of resistance to the combinatorial treatment are currently unknown and should be considered for further investigation in the future. The possible involvement of enhanced DNA repair function in the resistant cells may be considered. These mechanistic studies will eventually pave the way for developing effective therapeutic strategies to overcome the acquired resistance to the combinations such as sotorasib plus VP-16 and even for identifying potential biomarkers predicting the resistance.

It has been suggested that the inhibition of Topo IIβ is associated with cardiotoxicity caused by DXR (20). In our study, the tested KRAS G12C inhibitors sotorasib and adagrasib selectively decreased Topo IIα, but not Topo IIβ, levels. Topo I is also a well-known cancer target. KRAS G12C inhibitors did not alter Topo I levels in the tested KRAS G12C–mutant cancer cell lines. Accordingly, the combination of sotorasib with a Topo I inhibitor such as irinotecan or topotecan failed to show synergy in decreasing survival of KRAS G12C–mutant cancer cells. These results all indicate the selectivity of KRAS G12C inhibitors in suppressing Topo IIα and exhibiting synergy when combined with a Topo II inhibitor. We noted that the sotorasib and VP-16 combination synergistically decreased the survival of NSCLC cells with KRAS G12C mutation, but not those without this specific mutation, suggesting that the inclusion of VP-16 in this combination primarily augments the therapeutic efficacy of KRAS G12C inhibitors against cancer with KRAS G12 mutation. This also suggests that the combination may not show enhanced toxicity to normal tissues that usually do not have KRAS G12C mutation, which is supported by our data that the combination of sotorasib and VP-16 for about 4 weeks of treatment did not have any enhanced effects on damaging the tissues of major organs.

VP-16 is a Topo II inhibitor that causes DNA single- and double-strand breaks through interaction with DNA Topo II that prevents the religation of transiently breaking DNA (18). An interesting observation is that the combination of sotorasib and VP-16 effectively reduced the levels of Topo IIα in H1792 CDX tumors with primary resistance to sotorasib, whereas each drug alone had limited or no effect on decreasing Topo IIα levels under the tested conditions. This finding was also observed in H358/SR and PDX/SR tumors with acquired resistance to sotorasib, suggesting that VP-16 may enhance the ability of sotorasib to reduce Topo IIα levels. Similarly, treatment of sotorasib-resistant cell lines (H358/SR and Calu-1/SR) in which *TOP2A* expression was knocked down using both shRNA and siRNA further decreased Topo IIα levels, suggesting that targeting Topo IIα may further enhance the ability to decrease Topo IIα levels. The molecular mechanisms underlying these interesting observations are unclear, but the findings warrant further study in this direction.

In this study, we showed elevation of Topo IIα levels in DTPs but did not study the underlying mechanism largely due to very low numbers of DTPs surviving from sotorasib treatment. Despite this limitation, we have demonstrated that elevated Topo IIα levels in the tested sotorasib-resistant cell lines are due to increased Topo IIα protein stability because of downregulated FBXW7 and inactivated GSK3 in these resistant cell lines. Given the fact that DTPs are the key contributors to the emergence of acquired resistance to a given targeted therapy (12, 21, 22), it is plausible to assume that the mechanism accounting for Topo IIα elevation in sotorasib-resistant cells may be true in DTPs, warranting further validation in this direction. Another related limitation is that we did not conduct genetic manipulation of *TOP2A* expression in DTPs to study the role of Topo IIα in mediating the transient drug-tolerant state, largely due to technical challenges in manipulating the very low numbers of DTPs surviving from sotorasib treatment. This is another future research direction.

The mechanisms underlying acquired resistance to KRAS G12C inhibition in cancer are heterogeneous and involve target-dependent secondary mutations in the KRAS gene itself and target-independent mechanisms, such as MET amplification, activating mutations in other oncogenes (e.g., NRAS, BRAF) or fusions in other genes (e.g., ALK, RET), leading to activation of bypass pathways (23, 24). In our SR cell lines, we did not identify any new KRAS mutations or alterations in other key oncogenes, suggesting possible target-independent mechanisms involving alterations at the epigenetic, transcriptional, or posttranslational level. Findings from this study on Topo IIα elevation as a critical resistance mechanism to KRAS G12C inhibitors do not exclude or conflict with other possible target-independent mechanisms accounting for acquired resistance to KRAS G12C inhibitors, which are not the focus of this study.

In summary, the current study reveals an important connection between Topo IIα inhibition and therapeutic efficacy of RAS-targeted cancer therapy and suggests the value of Topo IIα expression as a biomarker to predict patient response and prognosis. Elevation of Topo IIα levels in DTPs and cells with acquired resistance to sotorasib is likely to be a key mechanism for these cells to survive or escape from a RAS-targeted therapy. Accordingly, targeting Topo IIα offers a potentially appealing strategy to enhance RAS-targeted cancer therapy through overcoming primary and acquired resistance and, particularly, through delaying or even preventing the development of acquired resistance (Supplemental Figure 13). The future clinical validation of this therapeutic strategy and of Topo IIα as a predictive biomarker is warranted.

## Methods

### Sex as a biological variable.

Lung cancer is not a sex-specific cancer. Both female and male mice were used in the study. Human lung cancer tissues were collected from male patients.

### Reagents.

Sotorasib (AMG510), adagrasib, VP-16, and DXR were purchased from MedChemExpress. Topo IIα (catalog 12286) and Bim (catalog 2933) antibodies were purchased from Cell Signaling Technology. Mcl-1 antibody (sc-12756) was purchased from Santa Cruz Biotechnology. γ-H2AX or phospho-H2AX (S139) antibody was purchased from MilliporeSigma (catalog 05-636). DAPI (catalog 62248), Alexa Fluor 488–donkey anti-mouse (A32766), and Alexa Fluor 568–donkey anti-rabbit (A10042) secondary antibodies were purchased from Thermo Fisher Scientific. Other reagents and antibodies were the same as described previously (9).

### Cell lines and cell culture.

H1792, H358, Calu-1, H23, H157, HCC827, PC-9, H460, A549, and Miacapa-2 were described previously (25–28). SW1463 and SW837 were provided by Lin Zhang (University of Southern California, Los Angeles, California). H1792, H358, Calu-1, H23, H157, HCC827, and PC-9 were genetically authenticated. The cell lines were cultured in RPMI 1640 medium (Coring) with 5% FBS in 5% CO_2_ humidified air. The sotorasib-resistant cell lines Calu-1/SR, H358/SR, and H23/SR were established by exposing cells to gradually increasing concentrations of sotorasib (starting at 100 nM and ending at 40 μM) for approximately 6 months. These cell lines had maintained resistance to sotorasib after withdrawal of sotorasib from the culture medium for up to 3 months, indicating an irreversible phenotype. The cell lines that stably express the ectopic *TOP2A* gene were established by cell infection using lentiviruses carrying a human *TOP2A* gene followed by hygromycin selection. pLenti-GIII-CMV-*TOP2A* (catalog 47306061) and matched control vector pLenti-GIII-CMV (catalog LV587) were purchased from abm.

### Western blot analysis.

Whole-cell protein lysates and subsequent immunoblotting were described previously (27). Protein band intensities were quantified using ImageJ software (NIH).

### Colony formation assay.

The indicated cells were seeded in 12-well plates at a density of 200 cells per well. After 24 hours, the tested drugs were added. The medium was replaced with fresh drugs every 3 days. Following a 10-day incubation period, the medium was removed. The cells were then fixed with 2% formalin and stained with 2% crystal violet in ethanol for colony counting and imaging.

### Cell survival assay.

Cells seeded in 96-well plates at appropriate densities for overnight incubation were exposed to the tested drugs either alone or in combination for 3 days. Cell numbers were estimated using sulforhodamine B (SRB) assay as previously described (29). CI for drug interaction was calculated using CompuSyn software (ComboSyn).

### Apoptosis assays.

Apoptosis was assessed using the Annexin V/7-AAD apoptosis detection kit (BD Biosciences) following the manufacturer’s instructions. Apoptosis was further confirmed by detecting protein cleavage with Western blotting.

### RT-qPCR.

The procedures for total cellular RNA extraction and *TOP2A* and *GAPDH* mRNA detection including primers were the same as described previously (9).

### Gene knockdown using siRNA and shRNA.

*TOP2A* siRNA (sc-36695), *TOP2A* shRNA plasmid (sc-36695-SH), and *Bim* siRNA (sc-29803) were purchased from Santa Cruz Biotechnology. Scrambled control and the procedures used for transfection or infection were described previously (30).

### IF staining.

Cell fixation and staining on chamber slides were the same as described previously (9). The primary antibody against Topo IIα or γ-H2AX was diluted at 1:100. The secondary Alexa Fluor 488-donkey anti-mouse or Alexa Fluor 568–donkey anti-rabbit antibody was diluted at 1:200. Images were collected using a confocal microscope (Leica TCS SP8).

### Detection of DTPs.

Cells were seeded in 12-well plates at approximately 90% confluence and treated with the tested drugs. The culture medium was replaced with fresh medium containing the same drugs every 3 days. After 5 or 10 days of incubation, the medium was removed, and the cells were fixed and stained with 2% crystal violet in ethanol to visualize DTPs

### IHC.

Detection of proteins of interest with IHC was basically the same as described previously (30). The dilutions of primary antibodies against Topo IIα, Ki-67, and cPARP were 1:100, 1:100, and 1:50, respectively. The IHC staining of Topo IIα in human KRAS G12C–mutant NSCLC tissues was the same as described previously (9).

### WGS for detection of gene mutations.

WGS was performed by Predicine. Genomic DNA (as low as 30 ng) was used for library preparation for each cell line. Genomic DNA processing, library processing, sequencing, and data analysis were performed similarly as previously described (31). For WGS, libraries were directly sequenced without enrichment. Each sample was sequenced to approximately 30× coverage. Sequencing data were analyzed using the Predicine DeepSea analysis pipeline for variant calling.

### Animal xenografts and treatments.

In conventional CDX studies, 4-week-old *nu*/*nu* nude mice (The Jackson Laboratory) were subcutaneously injected in the flank with 3 × 10^6^ cells suspended in sterile PBS. When the average tumor volume reached approximately 80 mm^3^, mice were randomly assigned into treatment groups with equal average tumor volumes and body weights. The following treatments were administered daily: vehicle control, sotorasib (50 mg/kg, oral gavage [og]), VP-16 (1 mg/kg, i.p.), and sotorasib plus VP-16. Tumor volumes were measured every 2–3 days using calipers and calculated with the formula V = π × (length × width^2^)/6. Body weights were recorded at the same intervals. At the end of the experiment, mice were euthanized using CO_2_ asphyxiation. Tumors were excised, weighed, and fixed in formalin for subsequent analysis. The same treatments were also applied to tumor-free immunocompetent C57BL/6J mice purchased from The Jackson Laboratory. Evaluation of major mouse organ tissues with H&E staining and biochemical tests of serum protein markers and blood cell counts were the same as described previously (9).

For the PDX study testing the efficacy of combination treatment on delaying the emergence of acquired resistance to sotorasib, lung cancer PDX (J000096652) harboring KRAS G12C mutation was purchased from The Jackson Laboratory and inoculated subcutaneously in nude mice. When the average tumor was around 100 mm^3^, the mice were grouped and treated as described above for varied periods of time depending on tumor size to observe resistance development.

PDX tumors that initially responded to sotorasib but gradually resumed growth after 2 months of continuous dosing were considered to have acquired resistance to sotorasib (PDX/SR). The tumors were then reimplanted into 4-week-old *nu*/*nu* nude mice for treatment experiments. When the average tumor size reached approximately 300 mm^3^, mice were randomized into 4 groups and treated daily with vehicle, sotorasib (50 mg/kg, og), VP-16 (1 mg/kg, i.p.), or the combination of sotorasib and VP-16. Tumor volume was measured using calipers every 2 or 3 days. At the end of the experiment, mice were sacrificed using CO_2_. The tumors were then removed, weighed, and stored in formalin for further analysis.

### Detection of Topo IIα in human KRAS G12C–mutant NSCLC tissues.

Tissue samples (*n* = 31) were collected either at baseline (i.e., at the time of diagnosis, *n* = 9) or before initiation of KRAS G12C inhibitor treatment (*n* = 22) from 31 patients with lung adenocarcinoma harboring KRAS G12C mutation and receiving treatment with KRAS G12C inhibitors between August 2022 and May 2025 in Tongji Hospital, Tongji Medical College, Huazhong University of Science and Technology. Topo IIα protein expression was evaluated by IHC as described previously and reviewed by 2 experienced pathologists independently. Topo IIα protein expression was semiquantitatively scored as WI based on calculation of % positive tumor cells × staining intensity. WI of 10 was used as a cutoff to define low (WI < 10) and high (WI ≥ 10) Topo IIα expression.

Demographic and clinicopathologic data were extracted from electronic medical records. Tumors were staged according to the eighth edition of the AJCC Cancer Staging Manual. Next-generation sequencing was performed on formalin-fixed paraffin-embedded tumor samples to confirm KRAS G12C mutation. Follow-up was conducted via medical record review and telephone interviews until September 20, 2025. PFS was defined as the time from KRAS G12C inhibitor initiation to disease progression, death, or last follow-up. ORR was defined as the proportion of patients with complete or partial response per RECIST v1.1 criteria.

### Statistics.

Survival curves were generated using the Kaplan-Meier method, with log-rank tests for subgroup comparisons. Statistical differences were determined by 2-sided unpaired Student’s *t* test, 1-way ANOVA, or Fisher’s exact test. Data are shown as the mean ± SD or SEM. All statistical analyses were conducted using GraphPad Prism 10 software or IBM SPSS Statistics 26.0. *P* values less than 0.05 were considered statistically significant.

### Study approval.

Animal experiments were approved by the IACUC of Emory University (PROTO201700718). The human subject study was approved by the Ethics Committee of Tongji Hospital, Tongji Medical College, Huazhong University of Science and Technology (TJ-IRB202406092).

### Data availability.

All data acquired specifically for this study, including raw Western blotting data, are available within the article itself and in the supplemental materials. Values for all data points in graphs are reported in the Supporting Data Values file.

## Author contributions

RX: methodology, investigation, formal analysis, data curation, visualization, and writing (original draft). DW: methodology and investigation. GM: methodology, investigation, and data curation. XY, formal analysis, data curation, review, and editing. QC: formal analysis, supervision, review, editing. SF: formal analysis, resources, review, and editing. RZ: investigation and formal analysis. PD: supervision and methodology. SJ: supervision, review, and editing. TAL and SSR: funding acquisition, review, and editing. ZC: conceptualization, supervision, methodology, investigation, formal analysis, data curation, visualization, and writing (original draft). SYS: conceptualization, supervision, funding acquisition, project administration, and writing (original draft).

## Funding support

- Winship Cancer Institute of Emory University internal funds.
- David A. Cole Professorship Fund.

## Supplementary Material

Supplemental data

Unedited blot and gel images

Supporting data values

## Version 1 02/16/2026

Electronic publication

## References

[1] Isermann T (2025). KRAS inhibitors: resistance drivers and combinatorial strategies. Trends Cancer.

[2] Ostrem JM (2013). K-Ras(G12C) inhibitors allosterically control GTP affinity and effector interactions. Nature.

[3] Oya Y (2024). The next-generation KRAS inhibitors...What comes after sotorasib and adagrasib?. Lung Cancer.

[4] de Langen AJ (2023). Sotorasib versus docetaxel for previously treated non-small-cell lung cancer with KRAS^G12C^ mutation: a randomised, open-label, phase 3 trial. Lancet.

[5] Lee ATM, Nagasaka M (2024). Adagrasib in KRYSTAL-12 has not broken the KRAS G12C enigma code of the unspoken 6-month PFS barrier in NSCLC. Lung Cancer (Auckl).

[6] Yakkala PA (2023). Prospects of topoisomerase inhibitors as promising anti-cancer agents. Pharmaceuticals (Basel).

[7] Nitiss JL (2009). Targeting DNA topoisomerase II in cancer chemotherapy. Nat Rev Cancer.

[8] Olaussen KA, Postel-Vinay S (2016). Predictors of chemotherapy efficacy in non-small-cell lung cancer: a challenging landscape. Ann Oncol.

[9] Chen Z (2024). DNA topoisomerase II inhibition potentiates osimertinib’s therapeutic efficacy in EGFR-mutant non-small cell lung cancer models. J Clin Invest.

[10] Chen MC (2011). Novel mechanism by which histone deacetylase inhibitors facilitate topoisomerase IIα degradation in hepatocellular carcinoma cells. Hepatology.

[11] Cabanos HF, Hata AN (2021). Emerging insights into targeted therapy-tolerant persister cells in cancer. Cancers (Basel).

[12] Sharma SV (2010). A chromatin-mediated reversible drug-tolerant state in cancer cell subpopulations. Cell.

[13] Hochmair MJ (2024). Sotorasib (960 mg or 240 mg) once daily in patients with previously treated KRAS G12C-mutated advanced NSCLC. Eur J Cancer.

[14] Hong DS (2020). KRAS^G12C^ Inhibition with sotorasib in advanced solid tumors. N Engl J Med.

[15] Ou SI (2022). First-in-human phase I/IB dose-finding study of adagrasib (MRTX849) in patients with advanced *KRAS*^G12C^ solid tumors (KRYSTAL-1). J Clin Oncol.

[16] Planchard D (2023). Osimertinib with or without chemotherapy in *EGFR*-mutated advanced NSCLC. N Engl J Med.

[17] Maione P (2025). New treatment strategies in advanced epidermal growth factor receptor-driven non-small cell lung cancer: beyond single agent osimertinib. Cancers (Basel).

[18] Jang JY (2025). Etoposide as a key therapeutic agent in lung cancer: mechanisms, efficacy, and emerging strategies. Int J Mol Sci.

[19] Carney DN (1991). The pharmacology of intravenous and oral etoposide. Cancer.

[20] Zhang S (2012). Identification of the molecular basis of doxorubicin-induced cardiotoxicity. Nat Med.

[21] Dhanyamraju PK (2022). Drug-tolerant persister cells in cancer therapy resistance. Cancer Res.

[22] Russo M (2024). Cancer drug-tolerant persister cells: from biological questions to clinical opportunities. Nat Rev Cancer.

[23] Awad MM (2021). Acquired resistance to KRAS^G12C^ inhibition in cancer. N Engl J Med.

[24] Chour A (2024). Mechanisms of resistance to KRASG12C inhibitors in KRASG12C-mutated non-small cell lung cancer. Front Oncol.

[25] Yao W (2015). Enhancing therapeutic efficacy of the MEK inhibitor, MEK162, by blocking autophagy or inhibiting PI3K/Akt signaling in human lung cancer cells. Cancer Lett.

[26] Ren H (2012). The combination of RAD001 and NVP-BKM120 synergistically inhibits the growth of lung cancer in vitro and in vivo. Cancer Lett.

[27] Shi P (2017). Overcoming acquired resistance to AZD9291, a third-generation EGFR inhibitor, through modulation of MEK/ERK-dependent Bim and Mcl-1 degradation. Clin Cancer Res.

[28] Kauh J (2010). c-FLIP degradation mediates sensitization of pancreatic cancer cells to TRAIL-induced apoptosis by the histone deacetylase inhibitor LBH589. PLoS One.

[29] Sun SY (1997). Differential effects of synthetic nuclear retinoid receptor-selective retinoids on the growth of human non-small cell lung carcinoma cells. Cancer Res.

[30] Chen Z (2021). Induction of SREBP1 degradation coupled with suppression of SREBP1-mediated lipogenesis impacts the response of EGFR mutant NSCLC cells to osimertinib. Oncogene.

[31] Renouf DJ (2022). The CCTG PA.7 phase II trial of gemcitabine and nab-paclitaxel with or without durvalumab and tremelimumab as initial therapy in metastatic pancreatic ductal adenocarcinoma. Nat Commun.

