# Supplementary material for: Cotargeting DNA topoisomerase II enhances efficacy of RAS-targeted therapy in KRAS-mutant cancer models
Source: J Clin Invest. 2026 Feb 16;136(4):e197192. doi: 10.1172/JCI197192 (PMC12904700; doi:10.1172/JCI197192)
Supplement: Supplemental data [file jci-136-197192-s263.pdf]

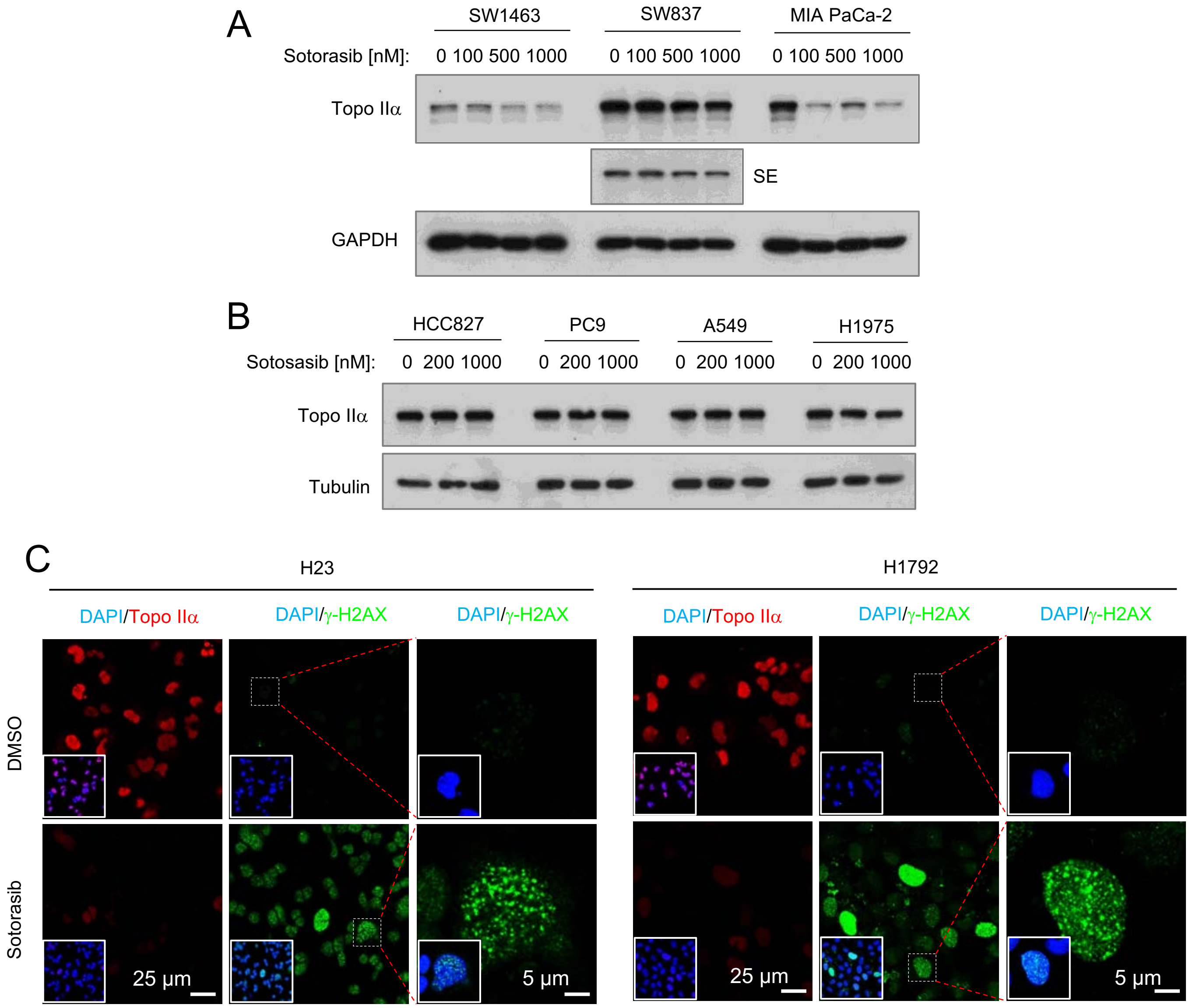

Fig. S1. Sotorasib decreases Topo IIα levels and induces DNA damage in KRAS G12C mutant cancer cell lines, but not in those without KRAS G12C mutation. *A* and *B*, The indicated cell lines were exposed to varied concentrations of sotorasib for 24 h. The proteins of interest were detected with Western blotting. *C*, H23 and H1792 were treated with DMSO, 500 nM (H23) or 1000 nM (H1792) sotorasib for 24 h followed by IF staining for detection of Topo IIα and γ-H2AX, respectively. SE, short exposure.

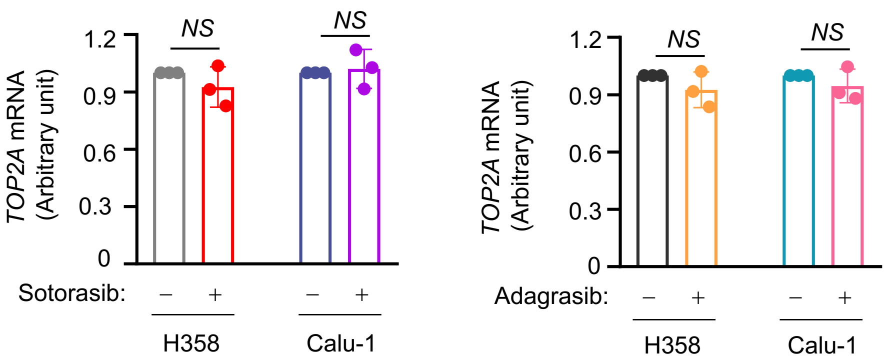

Figure S2. KRAS G12C inhibitors do not inhibit the expression of *TOP2A* gene in KRAS G12C mutant NSCLC cells. *A*, The given cell lines were exposed to 500 nM sotorasib or adagrasib for 16 h. *TOP2A* mRNA was detected with RT-qPCR. NS, not significant with two-sided unpaired Student's t-test.

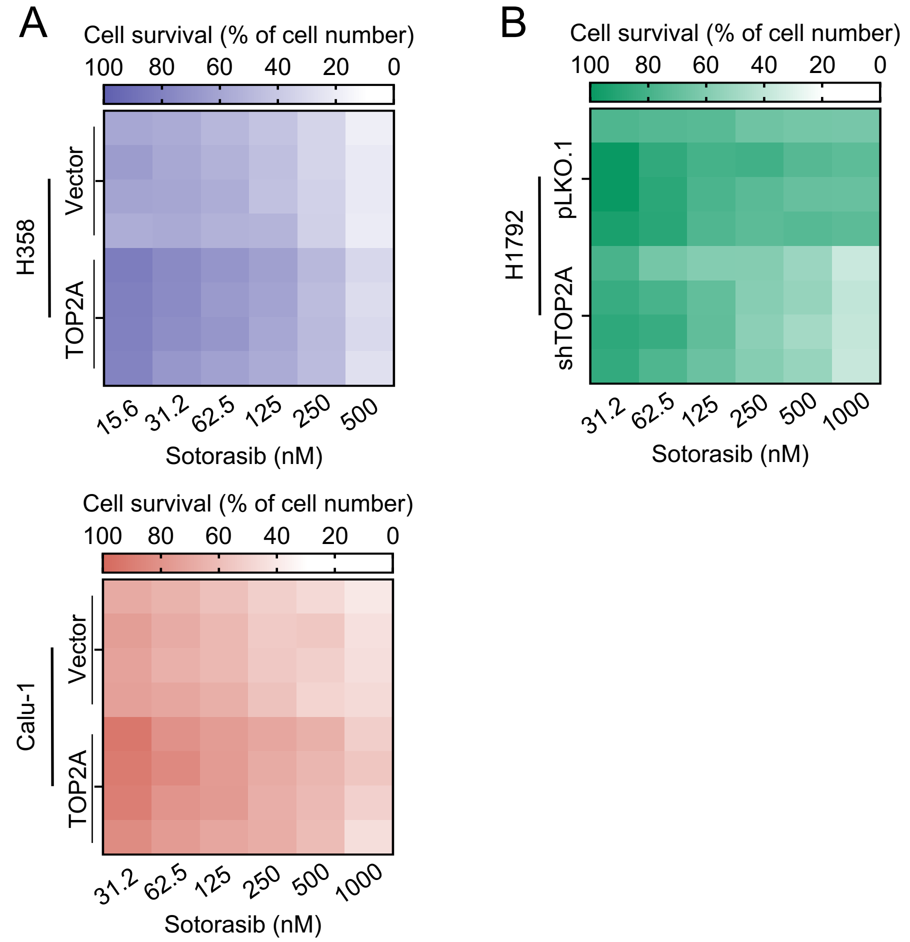

Figure S3. Modulation of Topo IIα levels in KRAS G12C mutant cell lines alters cell sensitivity to KRAS G12C inhibitors. *A*, Both H358 and Calu-1 cell lines expressing vector (V) or *TOP2A* were exposed to varied concentrations of sotorasib as indicated for 3 days. *B*, H1792 cells expressing pLKO.1 or shTOP2A were treated with different concentrations of sotorasib as indicated for 3 days. Cell numbers were estimated with the SRB assay. The data represent means ± SDs of four replicate determinations.

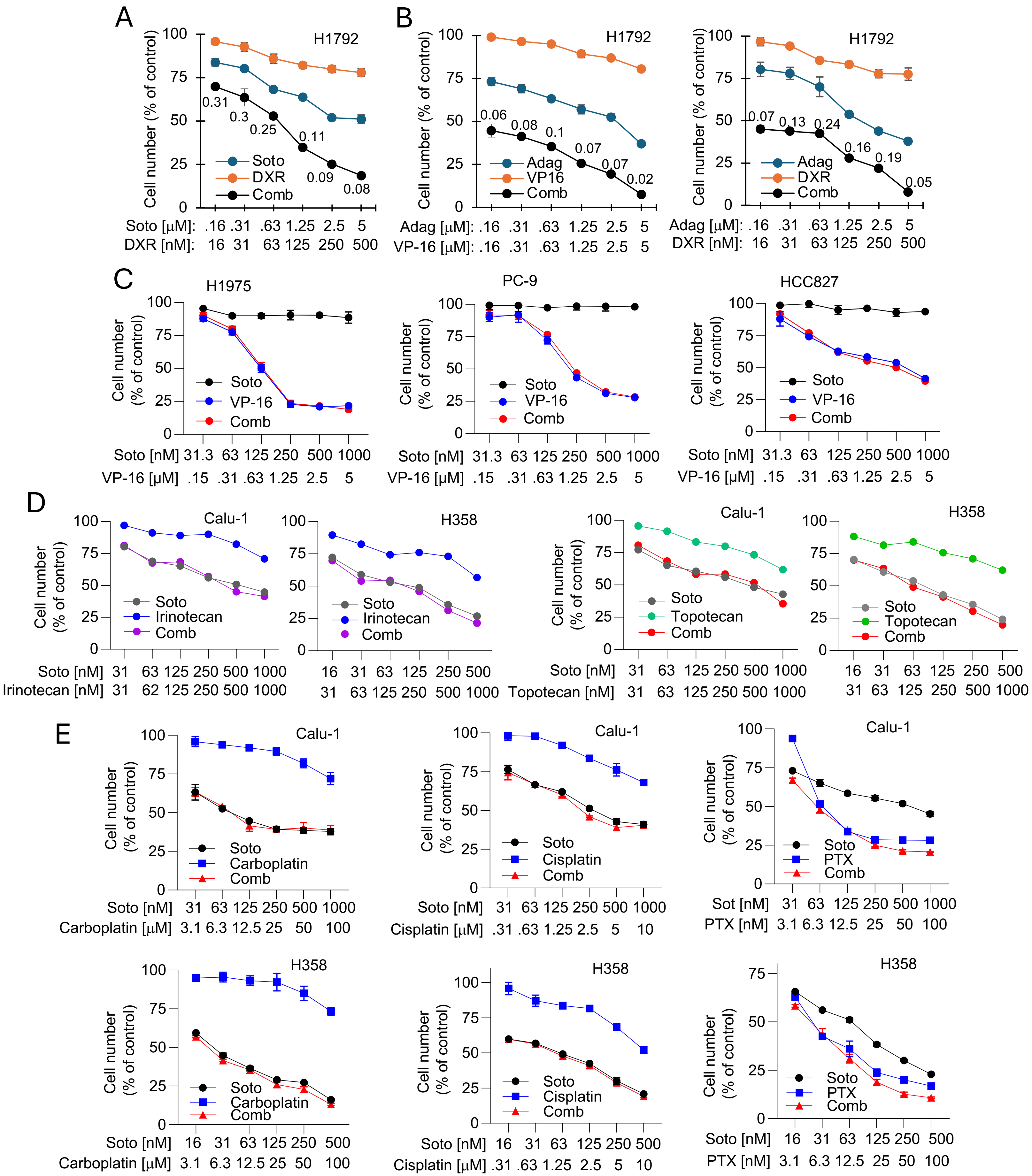

Fig. S4. Sotorasib or adagrasib combined with a Topo II inhibitor (*A* and *B*), but not a Topo I inhibitor (*D*) or a chemotherapeutic agent without Topo II-inhibitory activity (*E*), synergistically decreases the survival of KRAS G12C mutant NSCLC cells (*A*, *B*, *D* and *E*), but not other NSCLC cell lines without this mutation (*C*). The given cell lines in 96-well plates were treated with the varied concentrations of different tested agents alone or combinations as indicated for 3 days. Cell numbers were measured with the SRB assay. The data are the means ± SDs of four replicate determinations. PTX, paclitaxel.

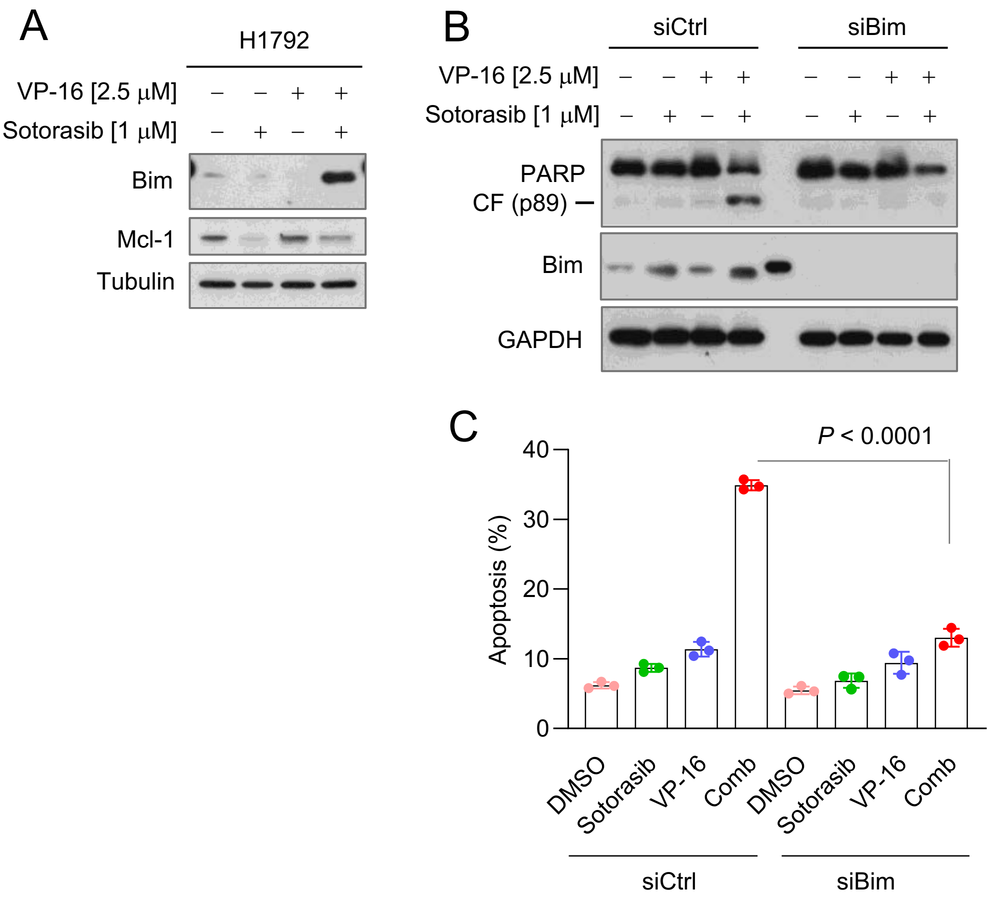

Fig. S5. Sotorasib and VP16 combination elevates Bim levels and induces Bim-dependent apoptosis in KRAS G12C mutant cancer cells. *A*, H1792 cells were treated with 1 μM sotorasib, 2.5 μM VP-16 or their combination for 24 h. *B* and *C*, H1792 cells transfected with control (Ctrl) or Bim siRNA for 24 h were exposed to 1 μM sotorasib, 2.5 μM VP-16 or their combination for 48 h. Proteins of interest were detected with Western blotting (*A* and *B*). Apoptotic cells were measured using annexin V staining/flow cytometry (*C*). The data in *C* are means ± SD of triplicate determinations. Statistical analysis was conducted with two-sided unpaired Student's t test.

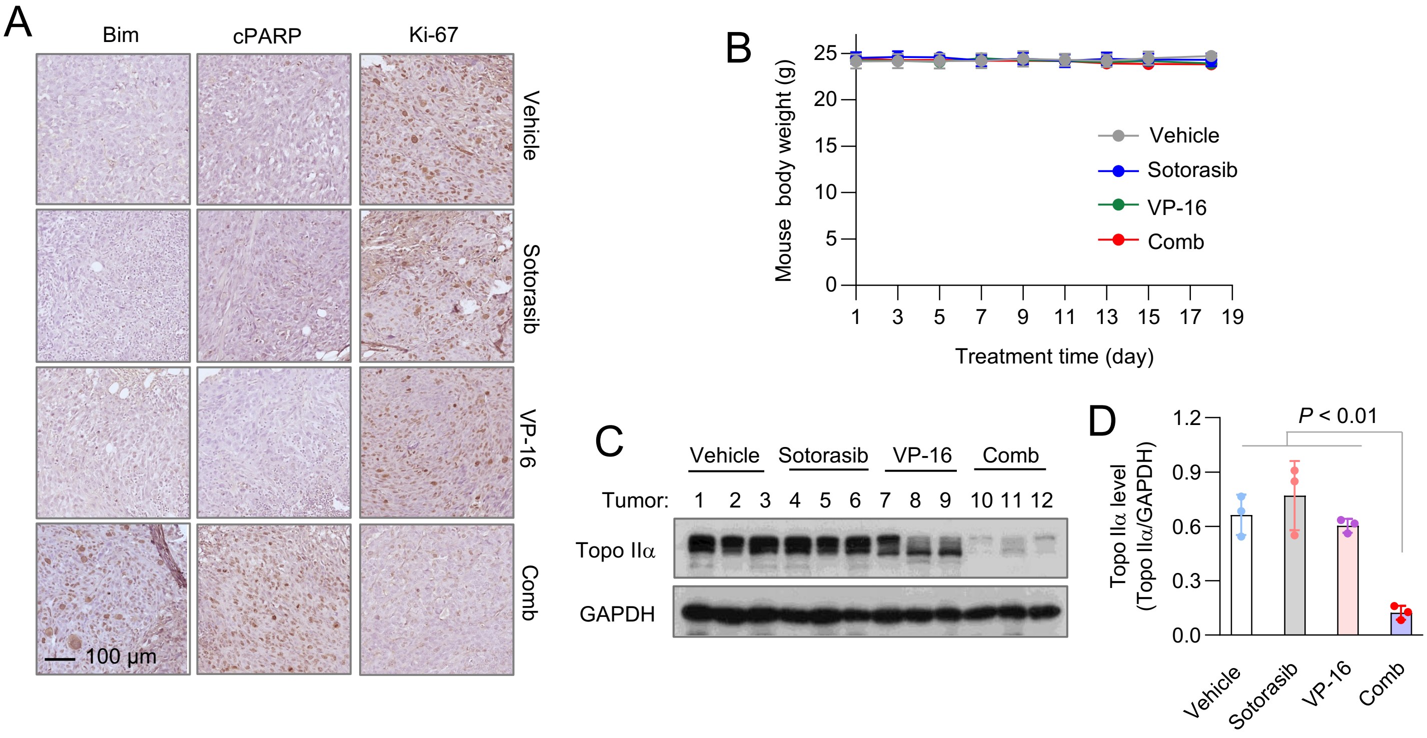

Fig. S6. The combination of sotorasib and VP16 does not reduce mouse body weights and enhances alteration of protein markers including Ki-67, Bim, cPARP and Topo IIα in H1792 CDX tumors. The mouse treatments were the same as described in Fig. 4. Proteins of interest were detected with IHC (*A*) or Western blotting (*C*) and band intensity was quantified using NIH ImageJ software (*D*). Mouse body weights were recorded at the indicated time (*B*). Statistical analysis was conducted with one-way ANOVA test..

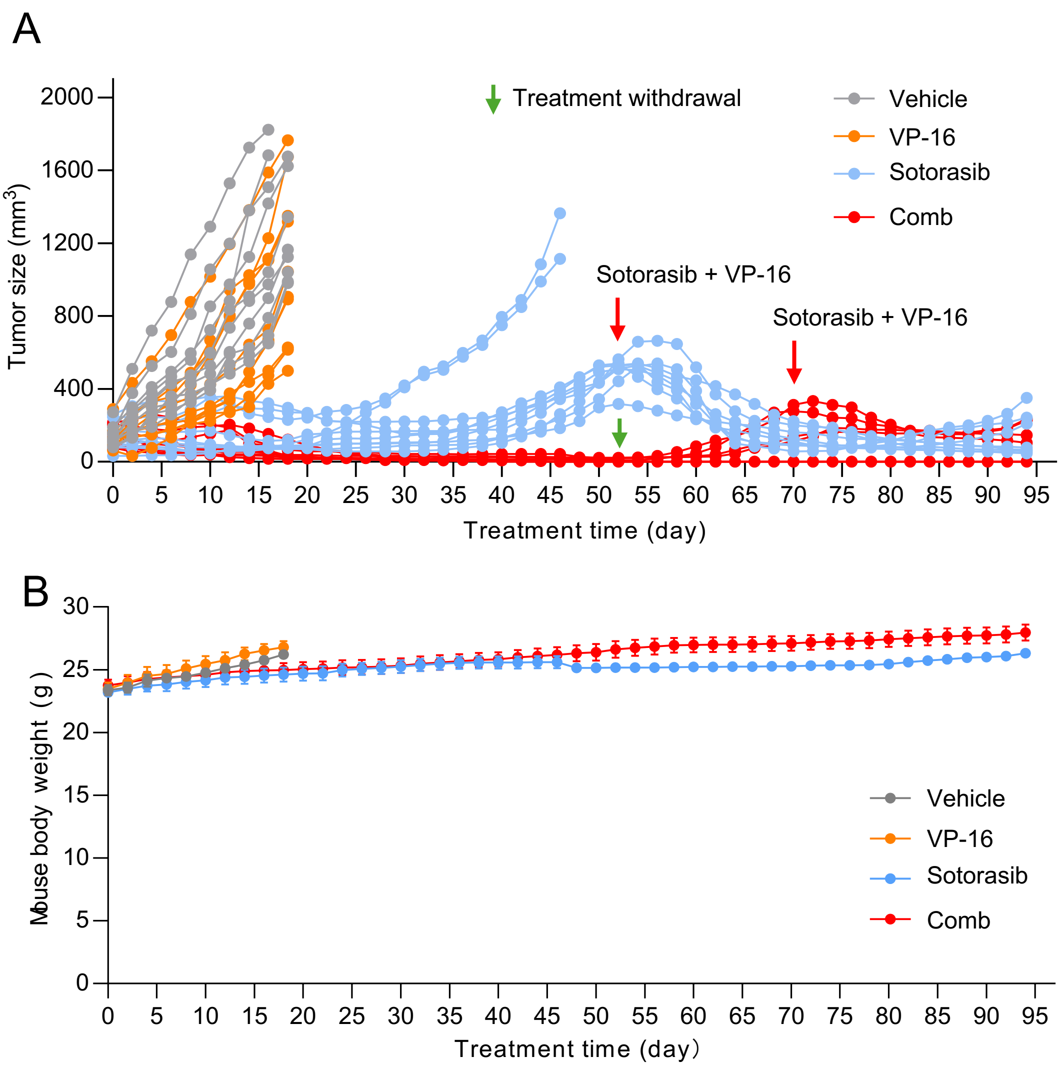

Fig. S7. The combination of sotorasib and VP16 effectively inhibits the growth of KRAS G12C mutant NSCLC PDXs and delays or prevents emergence of acquired resistance to sotorasib without reducing mouse body weights. The experimental procedure and treatments are the same as described in Fig. 5. There are 10 tumors in each treatment group (*A*) from 5 mice (*B*). The data in *B* are means ± SEs (n = 5/group).

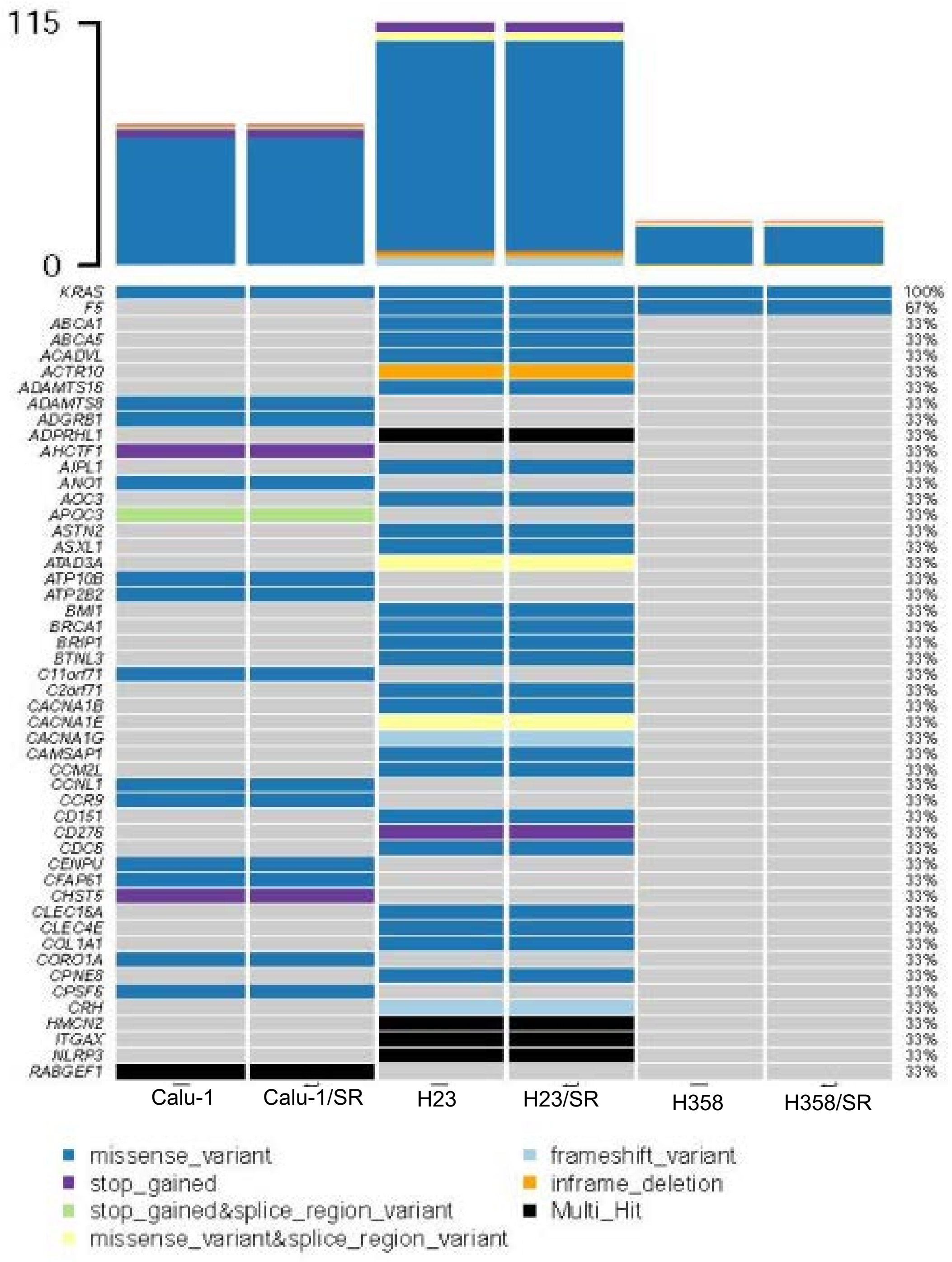

Fig. S8. Detection of KRAS G12C mutation and other gene alterations in SR cell lines in comparison with their corresponding parental cell lines. Top 50 genes showing mutations were listed. KRAS mutation refers to KRAS G12C mutation.

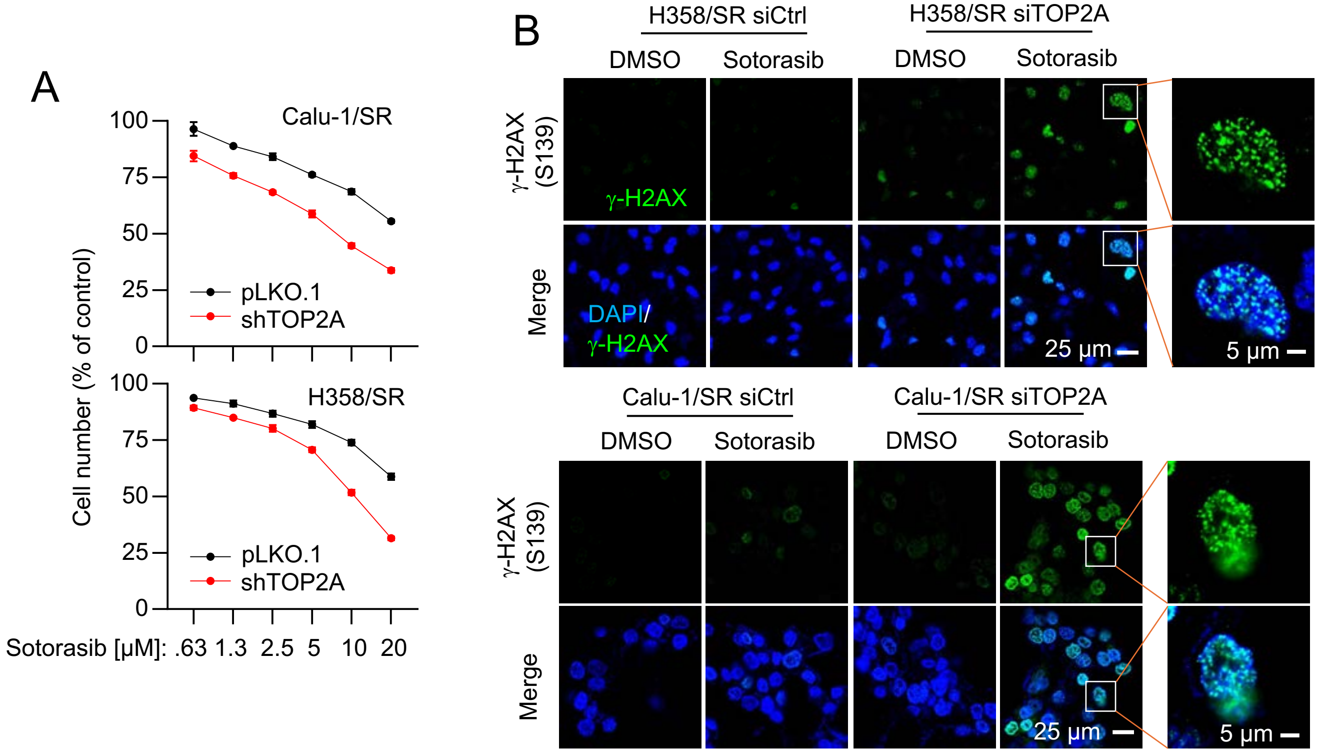

Fig. S9. Knockdown of *TOP2A* gene expression sensitizes sotorasib-resistant cells to sotorasib. *A*, The indicated cell lines were exposed to varied concentrations of sotorasib for 3 days. Cell numbers were estimated with the SRB assay. The results are means ± SDs of four replicate determinations. *B*, The indicated cell lines as described above transfected with control (Ctrl) or TOP2A siRNA were exposed to DMSO or 10 μM sotorasib for 48 h and then was stained using IF for γ-H2AX foci.

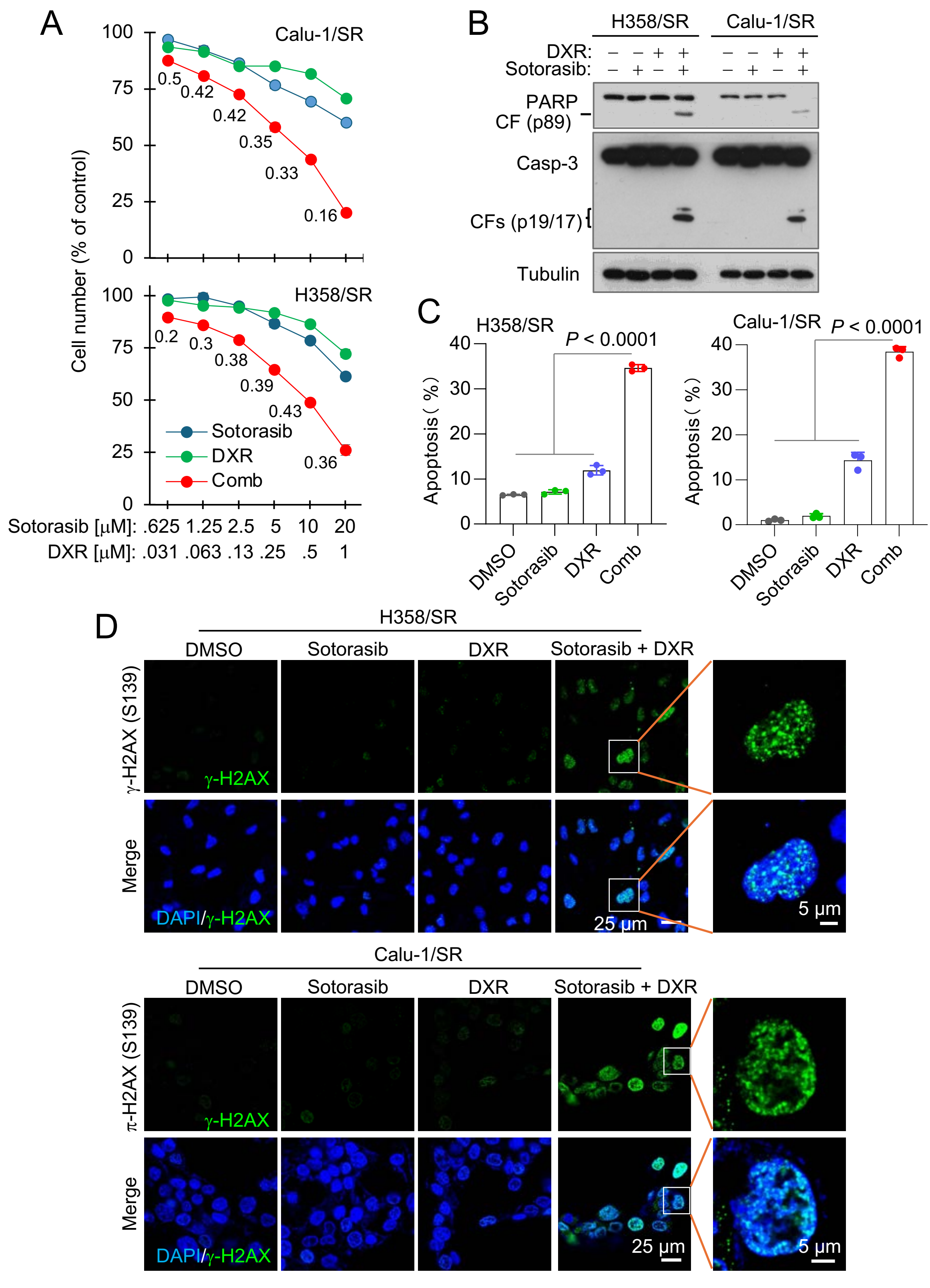

Fig. S10. Sotorasib and DXR combination synergistically decreases the survival of sotorasib-resistant cells with enhanced induction of apoptosis and DNA damage. *A*, The indicated cell lines were treated with varied concentrations of sotorasib, DXR or their combination for 3 days. Cell numbers were estimated with the SRB assay. The data are means ± SDs of four replicate determinations. *B-D*. Both indicated cell lines were exposed to 10 µM sotorasib, 0.5 µM DXR or their combination for 48 h (*B* and *C*) or 24 h (*D*). Proteins of interest were detected with Western blotting (*B*) or IF (*D*). Apoptosis was assayed with annexin V staining/flow cytometry (C). The data in *C* are means ± SDs of triplicate determinations. Statistical analysis was conducted with one-way ANOVA test.

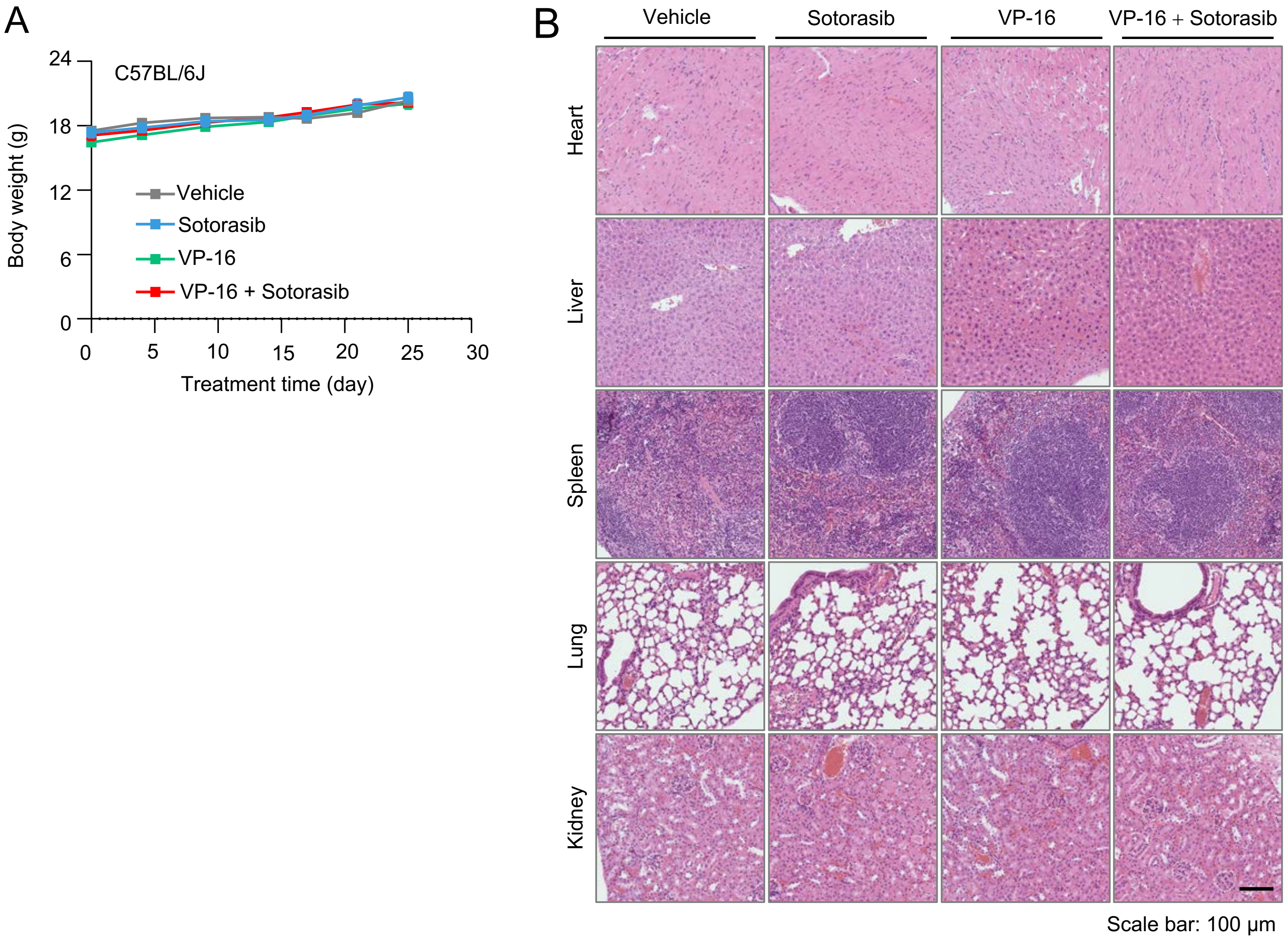

Fig. S11. The combination of sotorasib and VP-16 is well tolerated in immunocompetent mice in terms of body weight alterations (*A*) and histological examination of key organ tissues (*B*). Treatments were the same as described in Fig. 9.

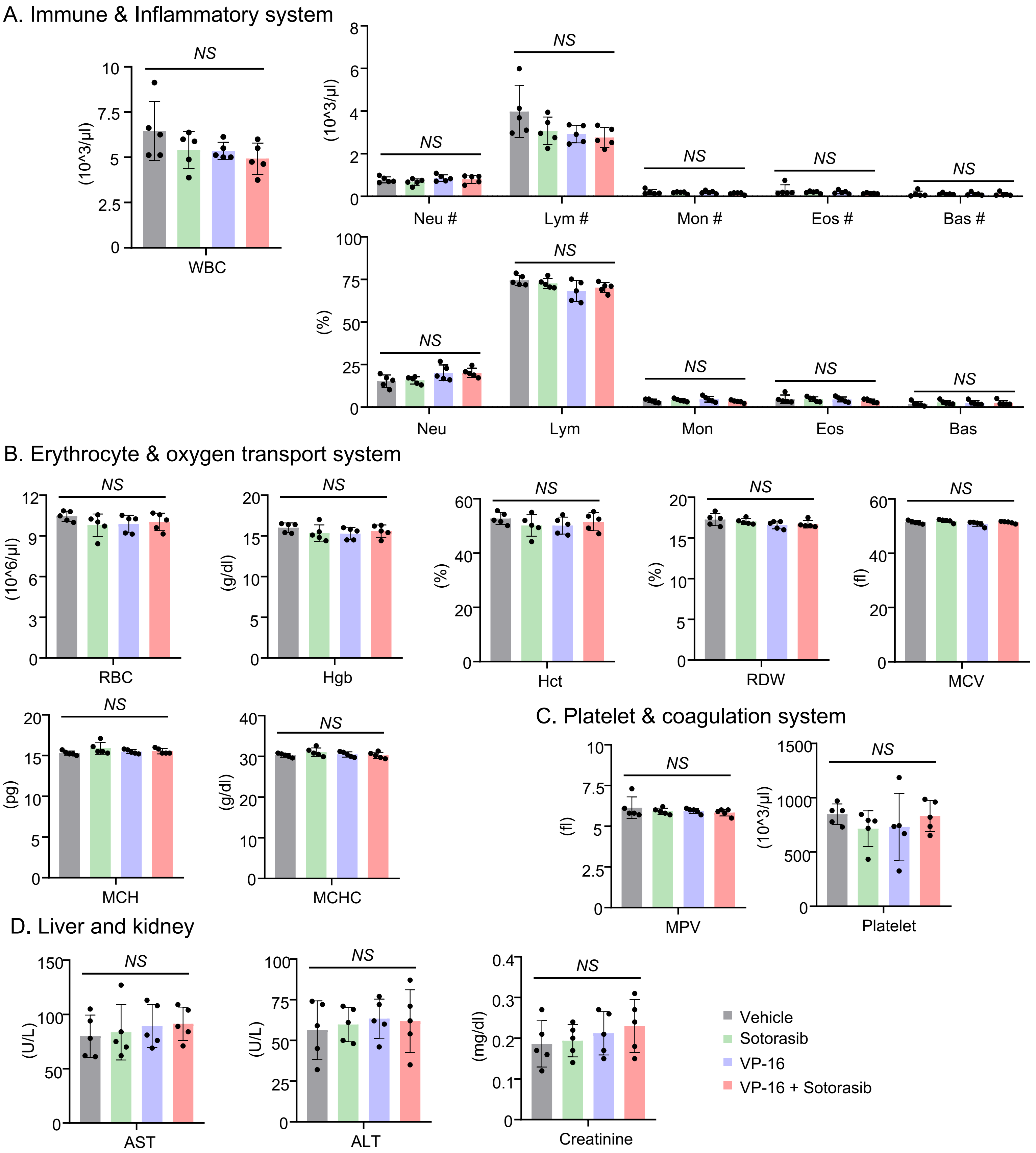

Fig. S12. The combination of sotorasib and VP-16 is well tolerated in immunocompetent mice in terms of evaluating blood cells and serum markers. Treatments were the same as described in Fig. 9. NS, not significant evaluated with one-way ANOVA test; RBC, red blood cell; WBC, white blood cells; AST, Aspartate aminotransferase; ALT, Alanine transaminase; MCV, mean corpuscular volume; MPV, mean platelet volume; Hgb, hemoglobin; MCHC, mean corpuscular hemoglobin concentration; MCH, mean corpuscular hemoglobin; Neu, neutrophils; Lym, lymphocytes; Mon, monocytes; Eos, eosinophil; Bas, basophil; Hct, hematocrit; RDW, red blood cell distribution width.

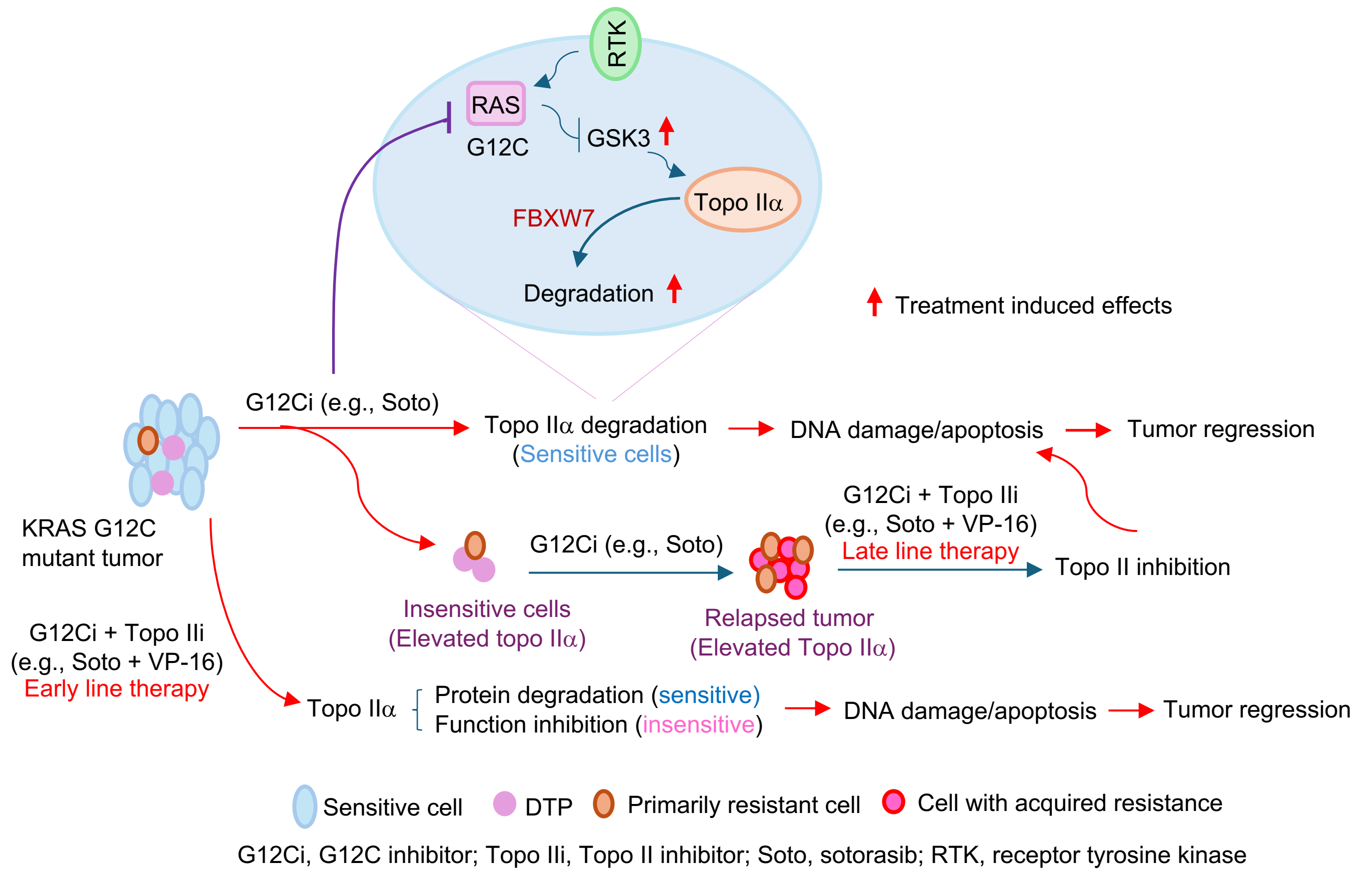

Fig. S13. A schematic illustration for mechanism by which KRAS G12C inhibition induced Topo IIα degradation, for the critical role of Topo IIα inhibition in mediating therapeutic efficacy of RAS-targeted cancer therapy, and for therapeutic strategies for enhancing RAS-targeted cancer therapy via co-targeting Topo II.

Table S1. Clinical characteristics of 31 patients with lung adenocarcinoma.

Characteristic	Number (%)
Age	
< 60	13 (41.9%)
≥ 60	18 (58.1%)
Gender	
Female	0
Male	31 (100%)
Smoking Status	
Never	8 (25.8%)
Current/ever	23 (74.2%)
Lymph nodes metastasis	
No	9 (29.0%)
Yes	22 (71.0%)
Brain metastasis	
No	22 (71.0%)
Yes	9 (29.0%)
Liver metastasis	
No	25 (80.6%)
Yes	6 (19.4%)
Adrenal gland metastasis	
No	22 (71.0%)
Yes	9 (29.0%)
Bone metastasis	
No	14 (45.2%)
Yes	17 (54.8%)
WI (% × intensity)	
< 10	19 (61.3%)
≥ 10	12 (38.7%)
