# Supplementary material for: Cotargeting DNA topoisomerase II enhances efficacy of RAS-targeted therapy in KRAS-mutant cancer models
Source: J Clin Invest. 2026 Feb 16;136(4):e197192. doi: 10.1172/JCI197192 (PMC12904700; doi:10.1172/JCI197192)
Supplement: Unedited blot and gel images [file jci-136-197192-s264.pdf]

Fig 1B

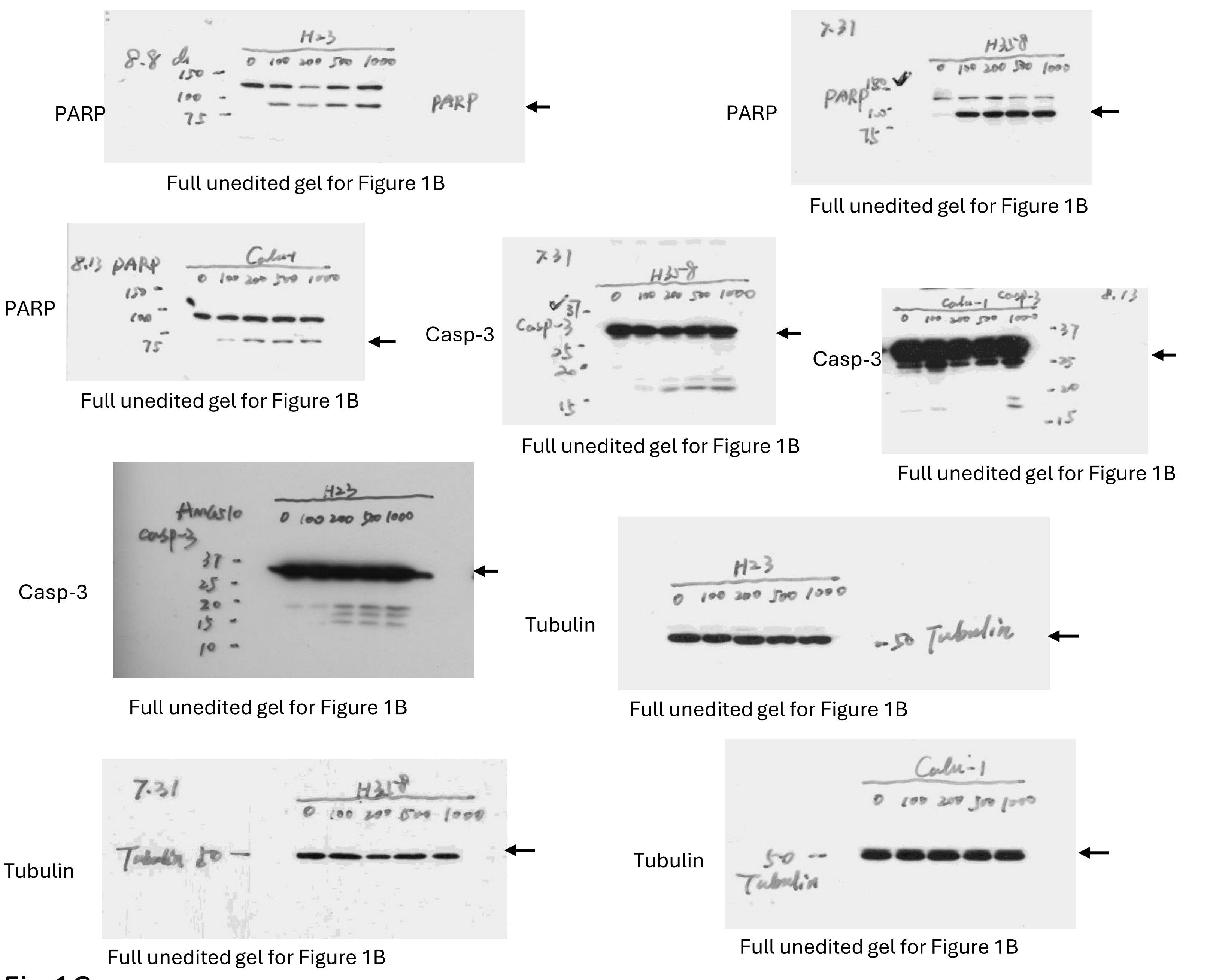

PARP
Full unedited gel for Figure 1B

PARP
Full unedited gel for Figure 1B

PARP
Full unedited gel for Figure 1B

Casp-3
Full unedited gel for Figure 1B

Casp-3
Full unedited gel for Figure 1B

Casp-3
Full unedited gel for Figure 1B

Tubulin
Full unedited gel for Figure 1B

Tubulin
Full unedited gel for Figure 1B

Tubulin
Full unedited gel for Figure 1B

Fig 1C

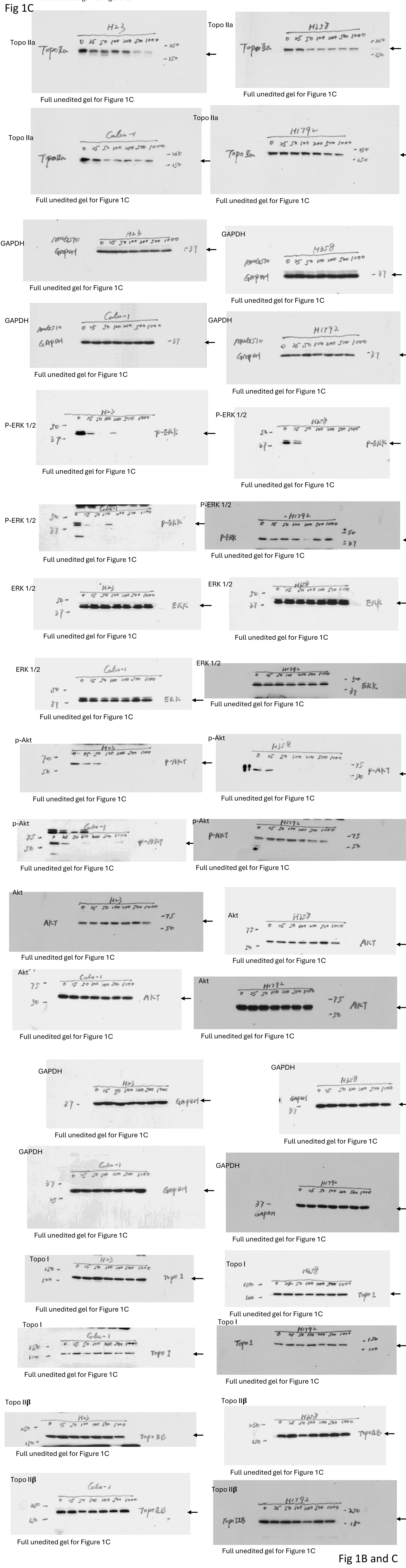

Topo IIa
Full unedited gel for Figure 1C

Topo IIa
Full unedited gel for Figure 1C

Topo IIa
Full unedited gel for Figure 1C

Topo IIa
Full unedited gel for Figure 1C

GAPDH
Full unedited gel for Figure 1C

GAPDH
Full unedited gel for Figure 1C

GAPDH
Full unedited gel for Figure 1C

GAPDH
Full unedited gel for Figure 1C

P-ERK 1/2
Full unedited gel for Figure 1C

P-ERK 1/2
Full unedited gel for Figure 1C

P-ERK 1/2
Full unedited gel for Figure 1C

P-ERK 1/2
Full unedited gel for Figure 1C

ERK 1/2
Full unedited gel for Figure 1C

ERK 1/2
Full unedited gel for Figure 1C

ERK 1/2
Full unedited gel for Figure 1C

ERK 1/2
Full unedited gel for Figure 1C

p-Akt
Full unedited gel for Figure 1C

p-Akt
Full unedited gel for Figure 1C

p-Akt
Full unedited gel for Figure 1C

p-Akt
Full unedited gel for Figure 1C

Akt
Full unedited gel for Figure 1C

Akt
Full unedited gel for Figure 1C

Akt
Full unedited gel for Figure 1C

Akt
Full unedited gel for Figure 1C

GAPDH
Full unedited gel for Figure 1C

GAPDH
Full unedited gel for Figure 1C

GAPDH
Full unedited gel for Figure 1C

GAPDH
Full unedited gel for Figure 1C

Topo I
Full unedited gel for Figure 1C

Topo I
Full unedited gel for Figure 1C

Topo I
Full unedited gel for Figure 1C

Topo I
Full unedited gel for Figure 1C

Topo IIβ
Full unedited gel for Figure 1C

Topo IIβ
Full unedited gel for Figure 1C

Topo IIβ
Full unedited gel for Figure 1C

Topo IIβ
Full unedited gel for Figure 1C

Fig 1B and C

Fig 1D

Topo IIa

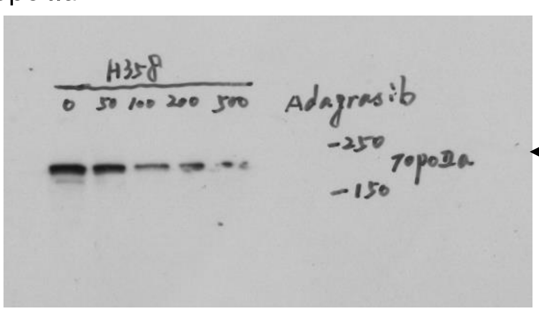

Full unedited gel for Figure 1D

Topo IIa

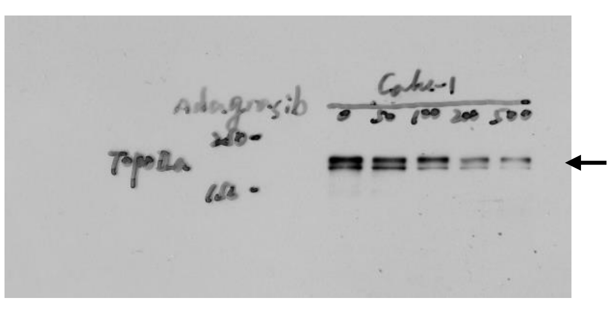

Full unedited gel for Figure 1D

P-ERK 1/2

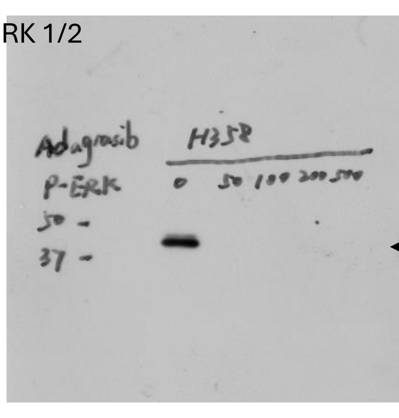

Full unedited gel for Figure 1D

P-ERK 1/2

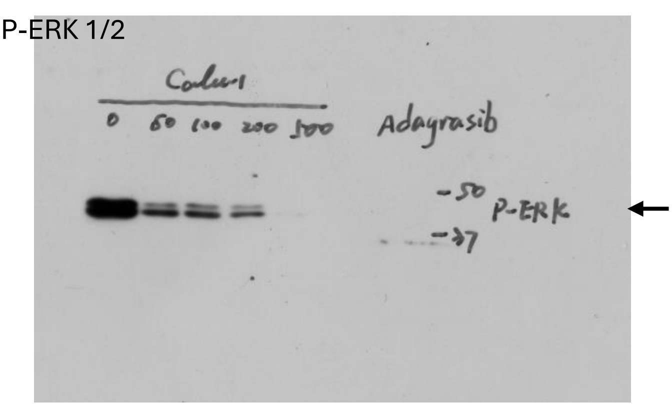

Full unedited gel for Figure 1D

ERK 1/2

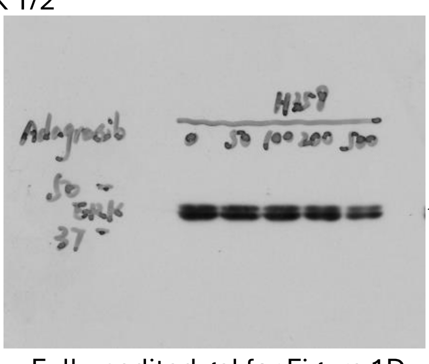

Full unedited gel for Figure 1D

ERK 1/2

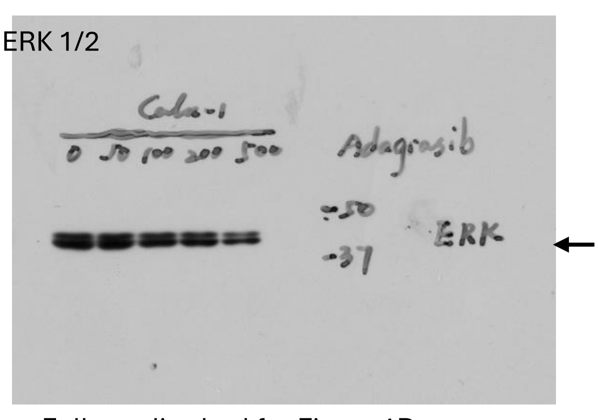

Full unedited gel for Figure 1D

p-Akt

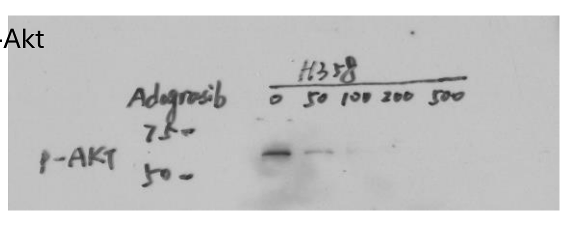

Full unedited gel for Figure 1D

p-Akt

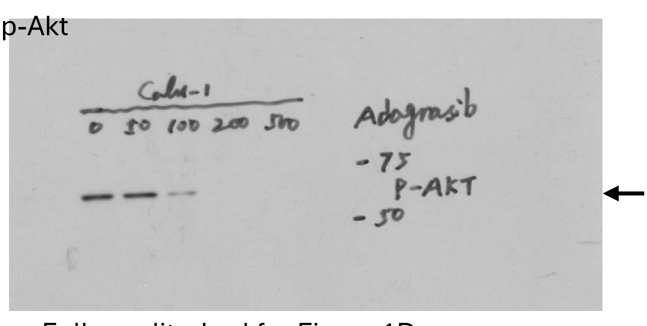

Full unedited gel for Figure 1D

Akt

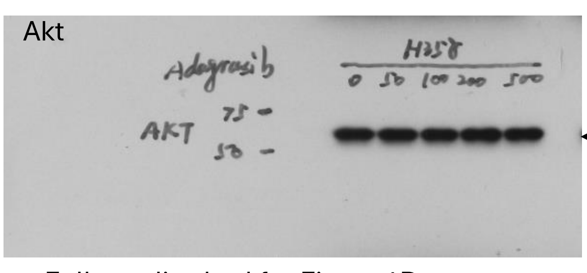

Full unedited gel for Figure 1D

Akt

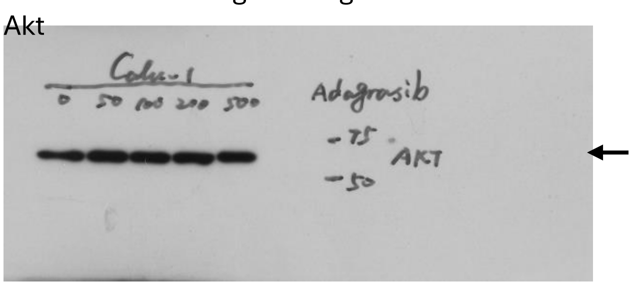

Full unedited gel for Figure 1D

Topo IIβ

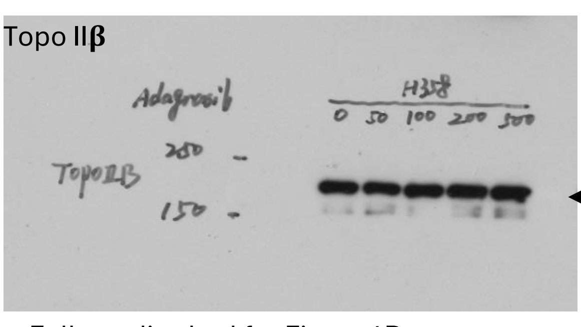

Full unedited gel for Figure 1D

Topo IIβ

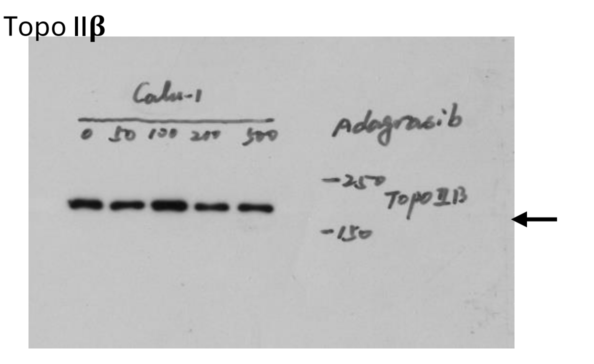

Full unedited gel for Figure 1D

Topo I

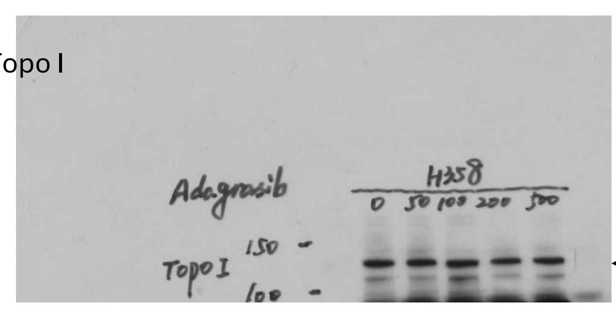

Full unedited gel for Figure 1D

Topo I

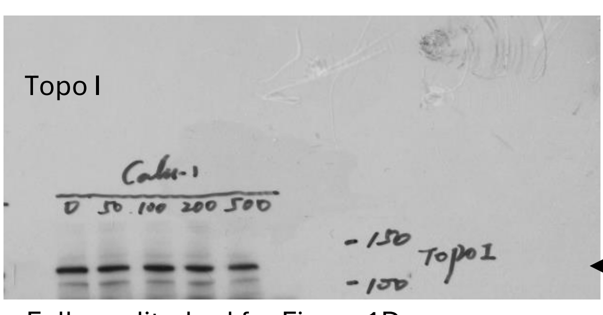

Full unedited gel for Figure 1D

Tubulin

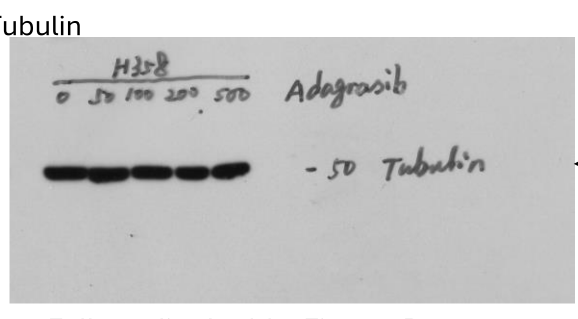

Full unedited gel for Figure 1D

Tubulin

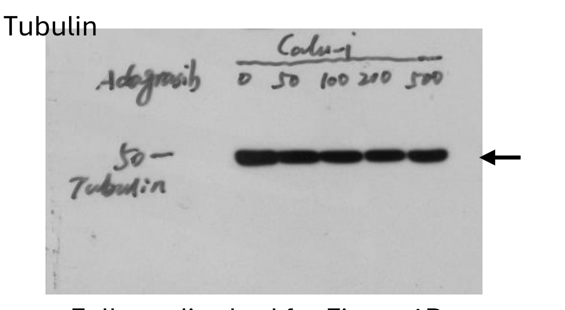

Full unedited gel for Figure 1D

Fig 1E

Topo IIa

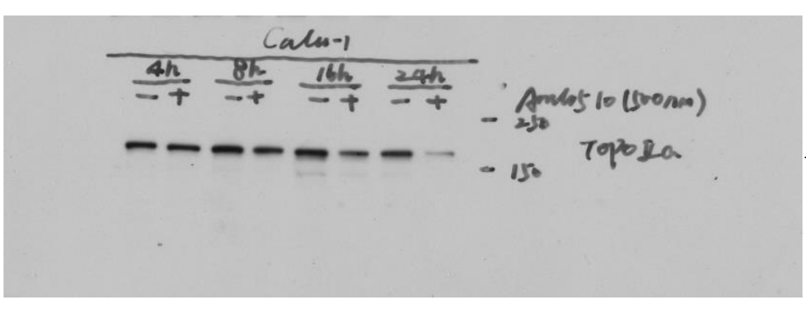

Full unedited gel for Figure 1E

GAPDH

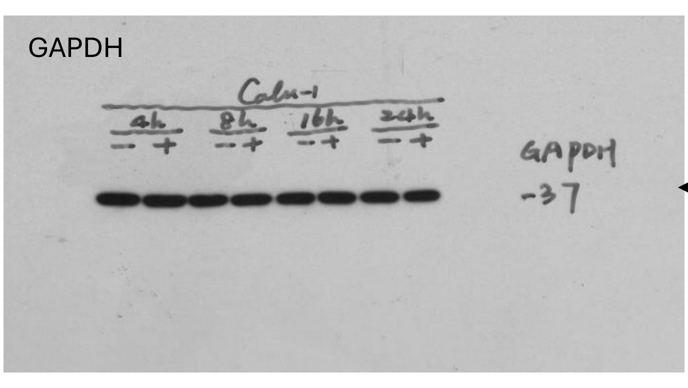

Full unedited gel for Figure 1E

Topo IIa

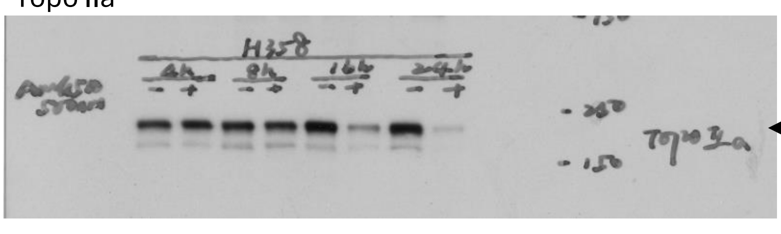

Full unedited gel for Figure 1E

GAPDH

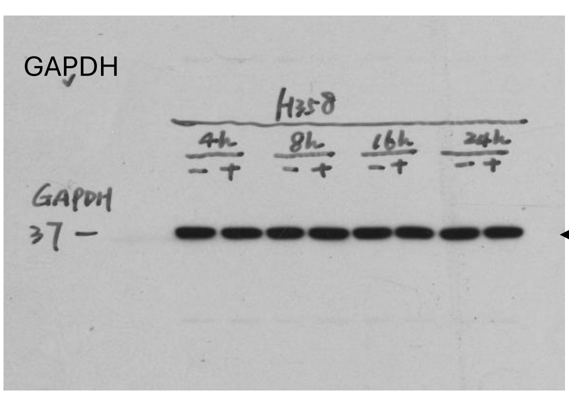

Full unedited gel for Figure 1E

Fig 1F

Tubulin

γ-H2AX

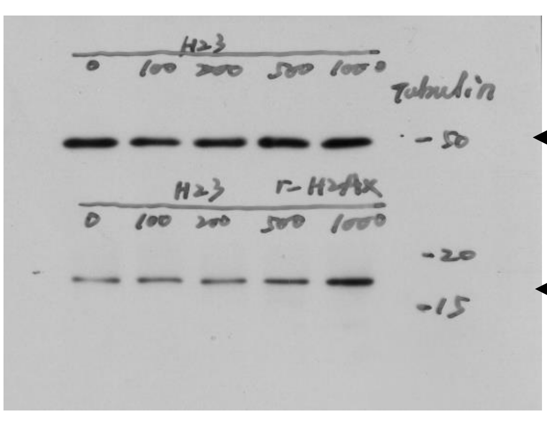

Full unedited gel for Figure 1F

γ-H2AX

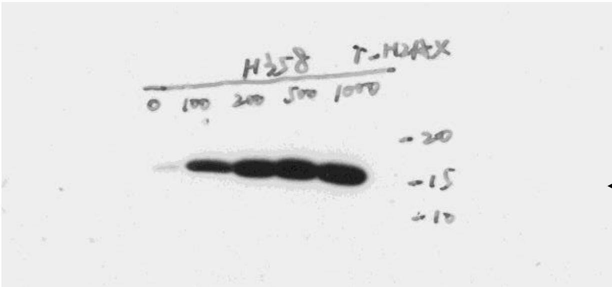

Full unedited gel for Figure 1F

Tubulin

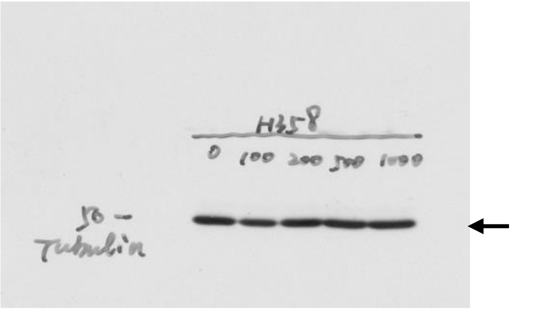

Full unedited gel for Figure 1F

γ-H2AX

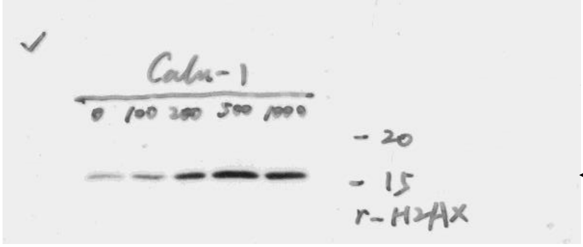

Full unedited gel for Figure 1F

Tubulin

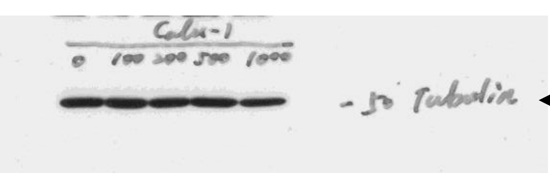

Full unedited gel for Figure 1F

Fig 1D-F

Fig 2A

Topo IIa

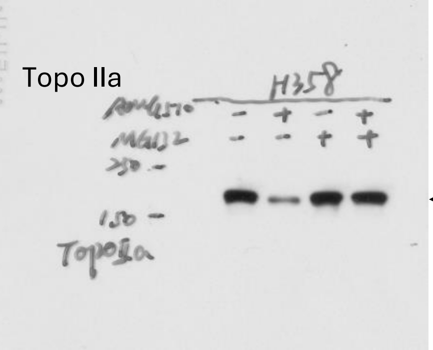

Full unedited gel for Figure 2A

Tubulin

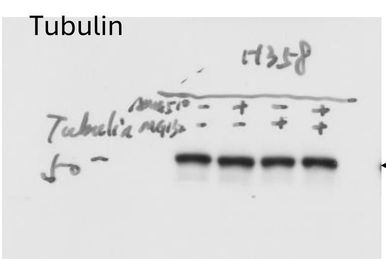

Full unedited gel for Figure 2A

Topo IIa

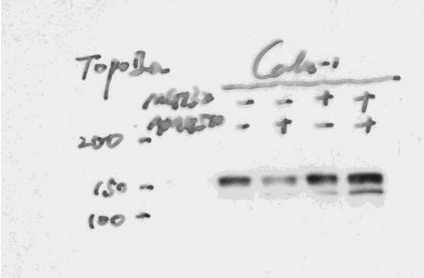

Full unedited gel for Figure 2A

Tubulin

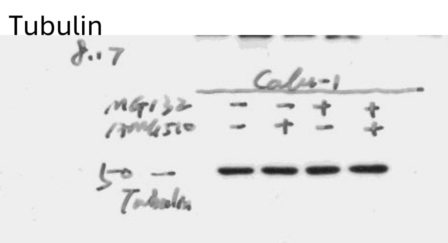

Full unedited gel for Figure 2A

Topo IIa

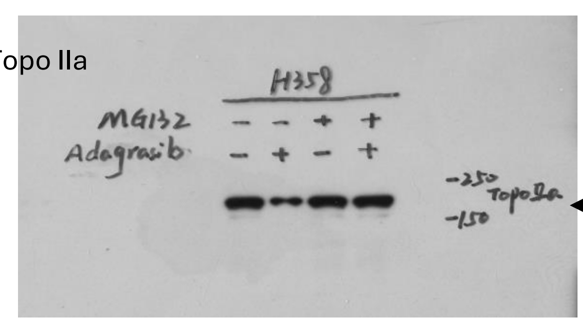

Full unedited gel for Figure 2A

Tubulin

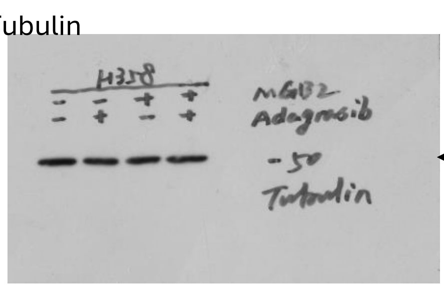

Full unedited gel for Figure 2A

Topo IIa

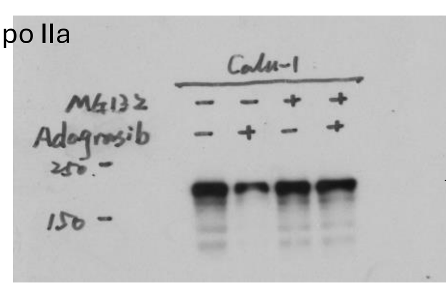

Full unedited gel for Figure 2A

Tubulin

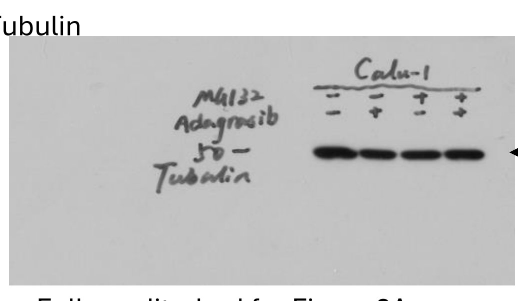

Full unedited gel for Figure 2A

Fig 2B

Topo IIa

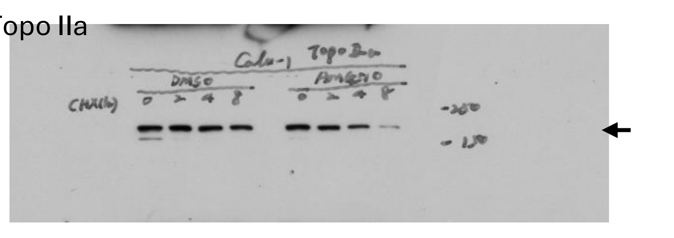

Full unedited gel for Figure 2B

Topo IIa

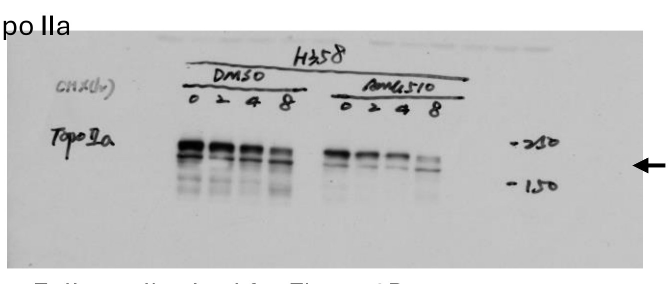

Full unedited gel for Figure 2B

GAPDH

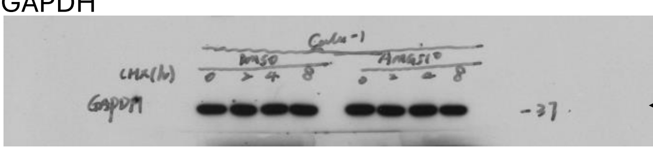

Full unedited gel for Figure 2B

GAPDH

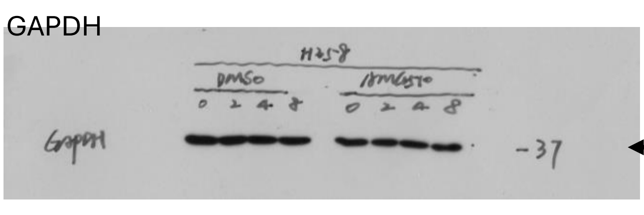

Full unedited gel for Figure 2B

Topo IIa

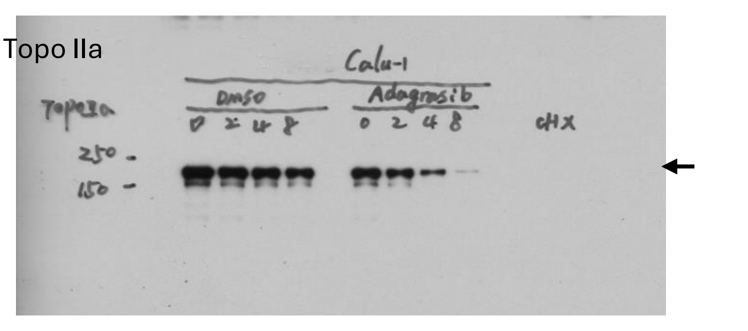

Full unedited gel for Figure 2B

GAPDH

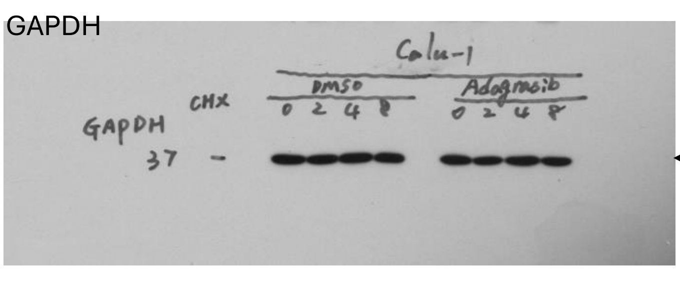

Full unedited gel for Figure 2B

Topo IIa

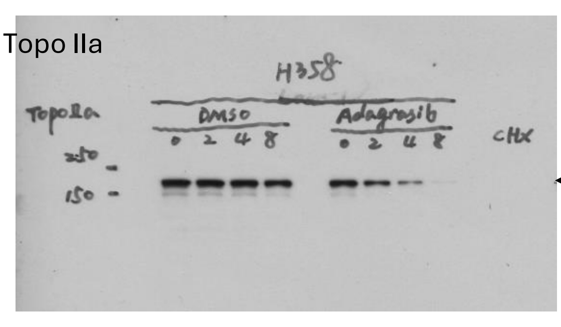

Full unedited gel for Figure 2B

GAPDH

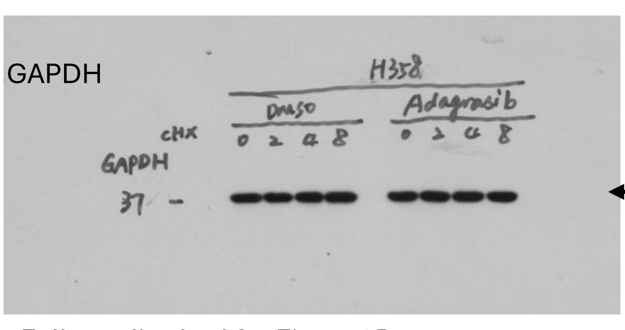

Full unedited gel for Figure 2B

Fig 2C

Topo IIa

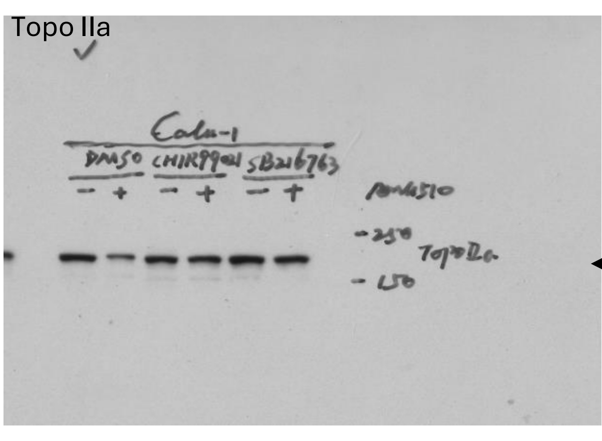

Full unedited gel for Figure 2C

GAPDH

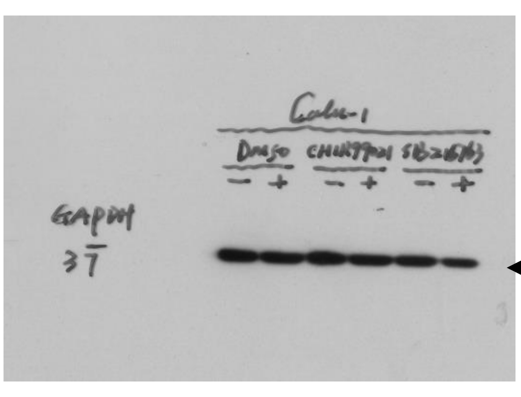

Full unedited gel for Figure 2C

Topo IIa

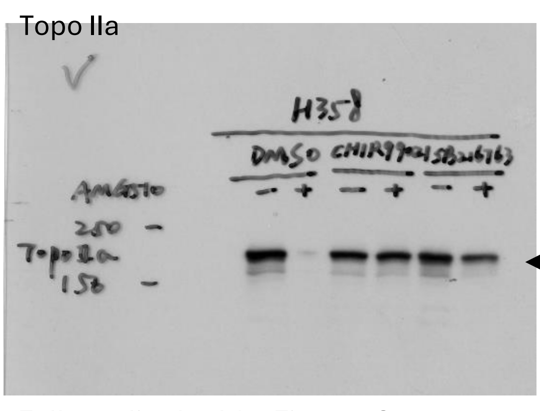

Full unedited gel for Figure 2C

GAPDH

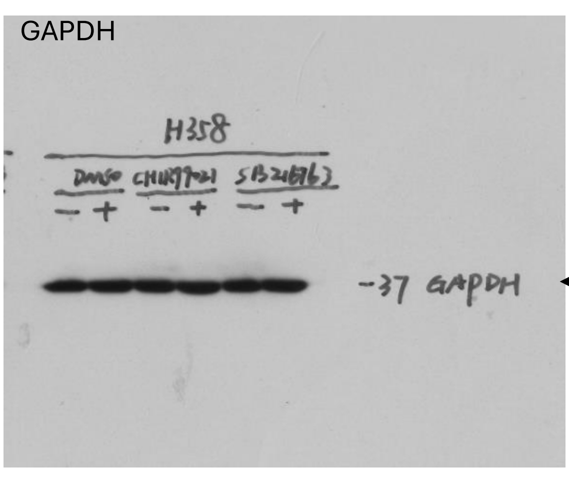

Full unedited gel for Figure 2C

Fig 2D

Topo IIa

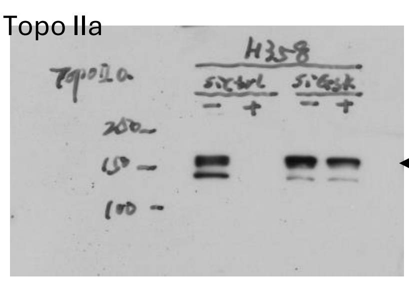

Full unedited gel for Figure 2D

GSK3α/β

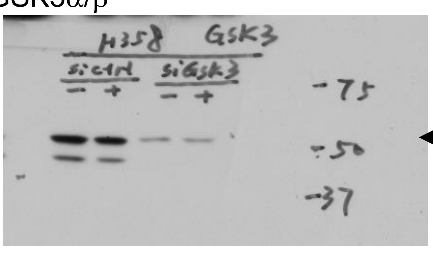

Full unedited gel for Figure 2D

GAPDH

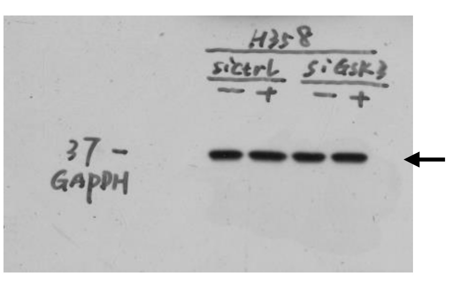

Full unedited gel for Figure 2D

Topo IIa

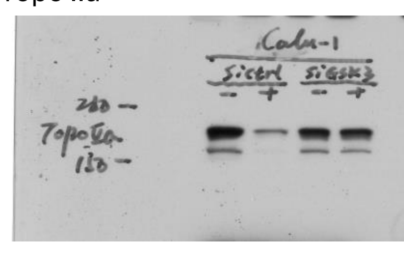

Full unedited gel for Figure 2D

GSK3α/β

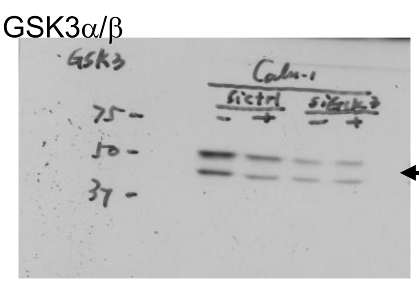

Full unedited gel for Figure 2D

GAPDH

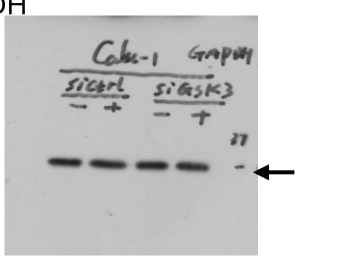

Full unedited gel for Figure 2D

Fig 2E

Topo IIa

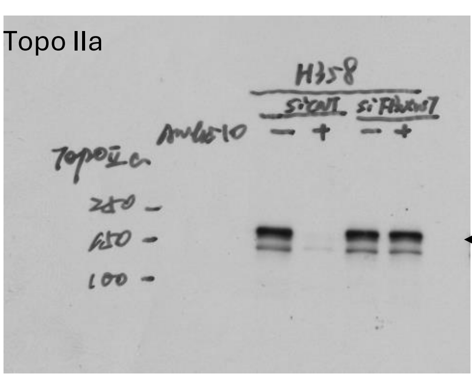

Full unedited gel for Figure 2E

FBXW7

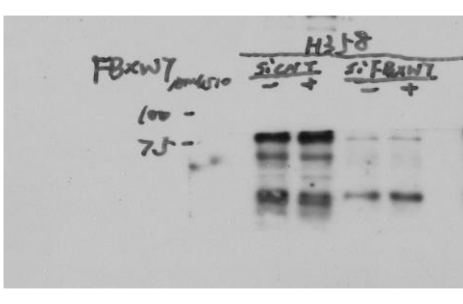

Full unedited gel for Figure 2E

GAPDH

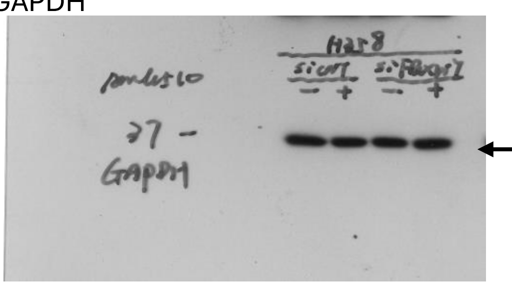

Full unedited gel for Figure 2E

Topo IIa

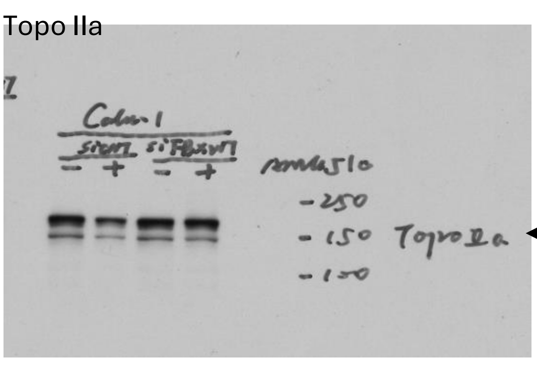

Full unedited gel for Figure 2E

FBXW7

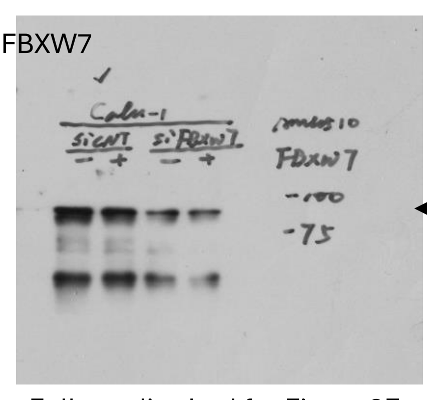

Full unedited gel for Figure 2E

GAPDH

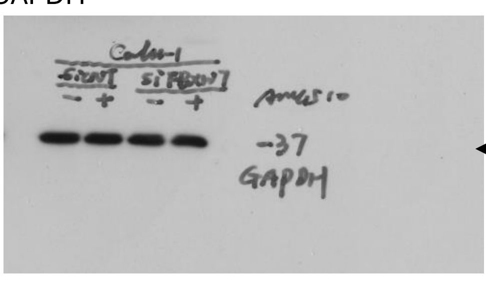

Full unedited gel for Figure 2E

Fig 2

Fig 3A

PARP

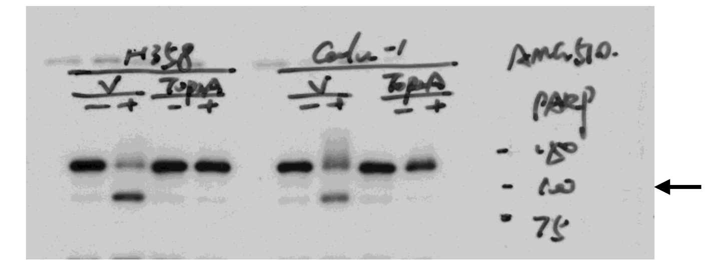

Full unedited gel for Figure 3A

Topo IIa

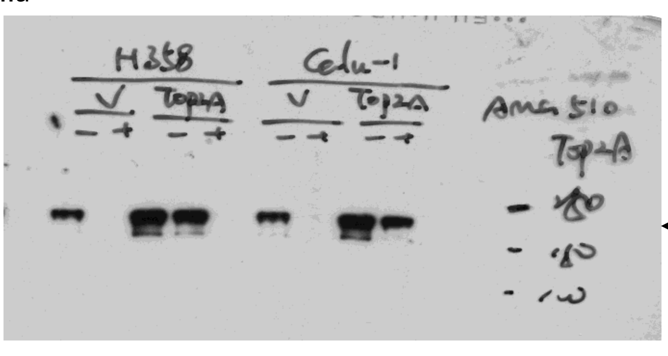

Full unedited gel for Figure 3A

GAPDH

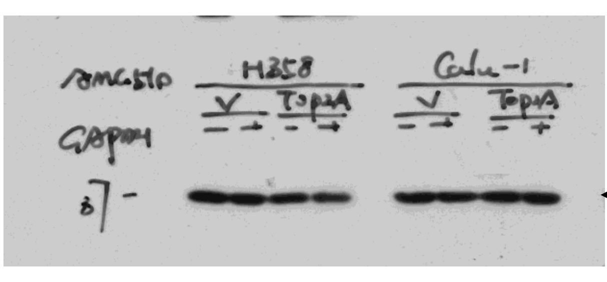

Full unedited gel for Figure 3A

Fig 3E

Topo IIa

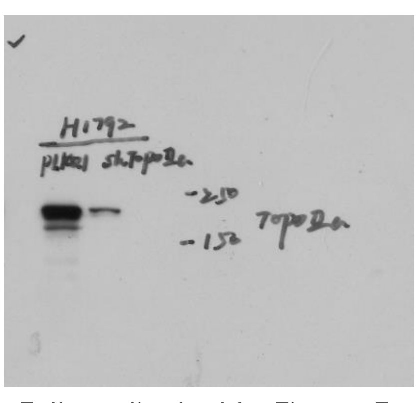

Full unedited gel for Figure 3E

GAPDH

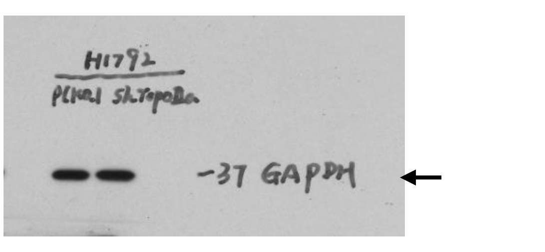

Full unedited gel for Figure 3E

Fig 3G

Topo IIa

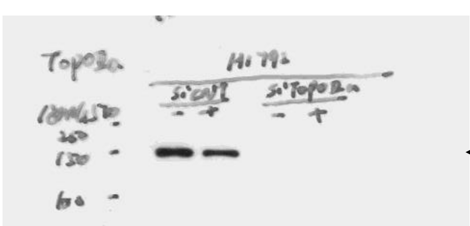

Full unedited gel for Figure 3G

PARP

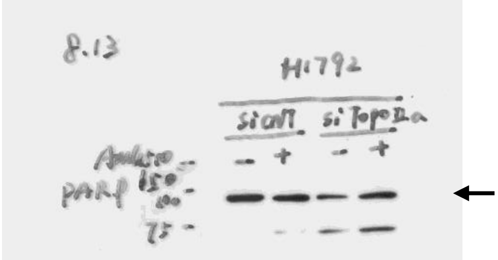

Full unedited gel for Figure 3G

Tubulin

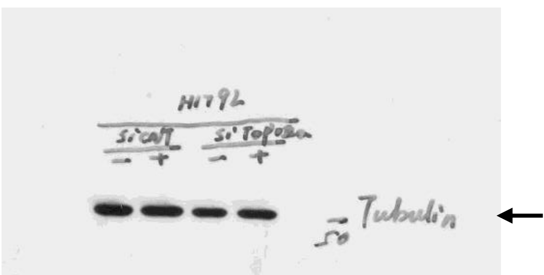

Full unedited gel for Figure 3G

Fig 3

Fig 4C

PARP

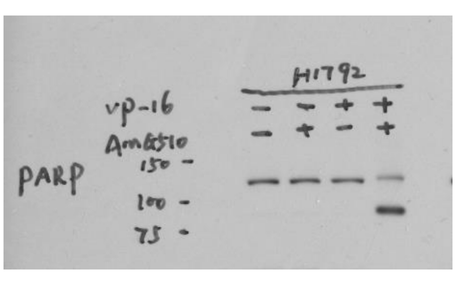

Full unedited gel for Figure 4C

Tubulin

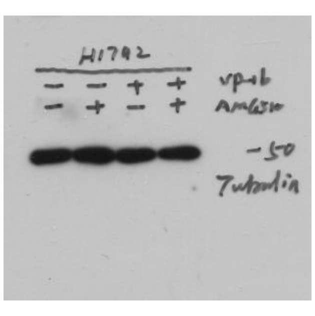

Full unedited gel for Figure 4C

Fig 4

Fig 4F

PARP

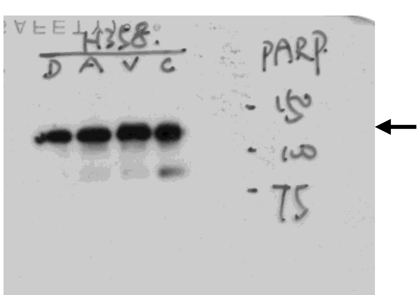

Full unedited gel for Figure 4F

rH2AX

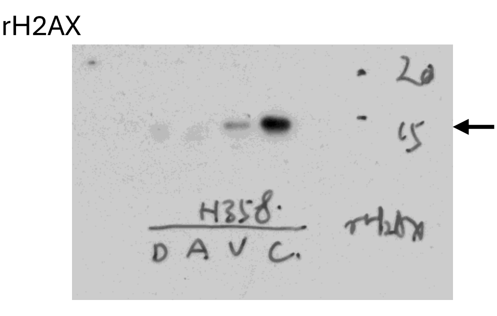

Full unedited gel for Figure 4F

Tubulin

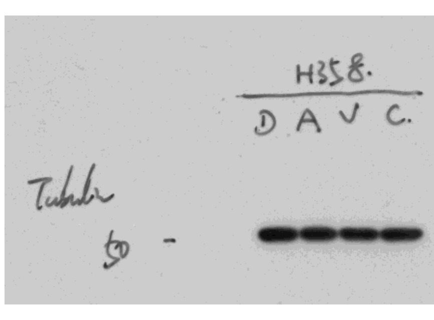

Full unedited gel for Figure 4F

Fig 4

Fig 5C

Topo IIa

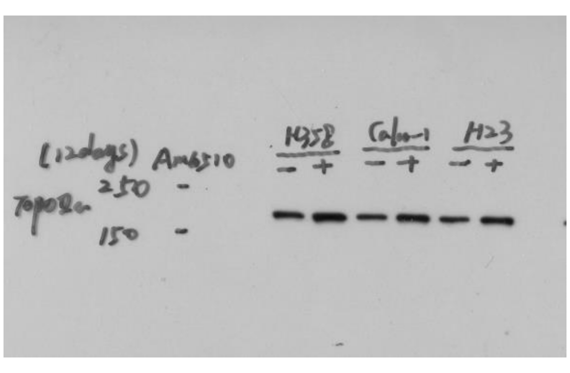

Full unedited gel for Figure 5B

Topo IIa

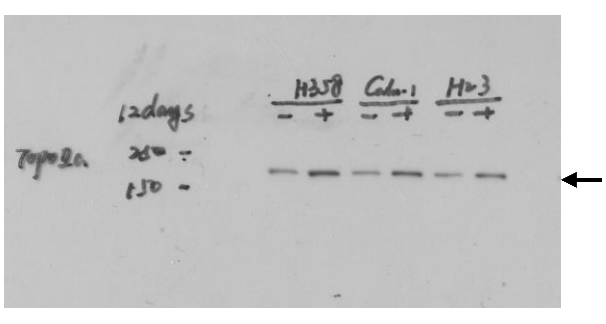

Full unedited gel for Figure 5B

Fig 5

GAPDH

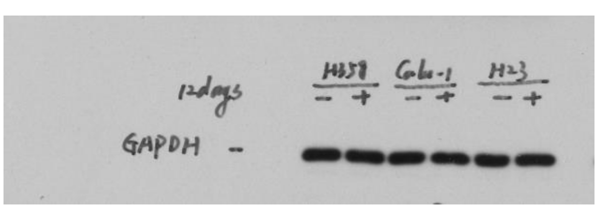

Full unedited gel for Figure 5B

GAPDH

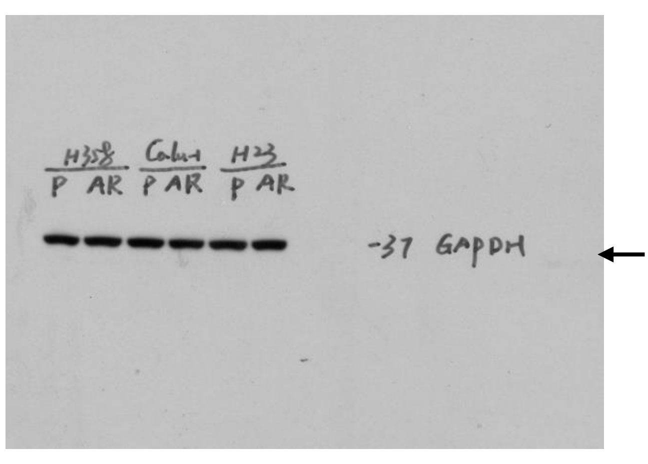

Full unedited gel for Figure 6B

Fig 6B

Topo IIa

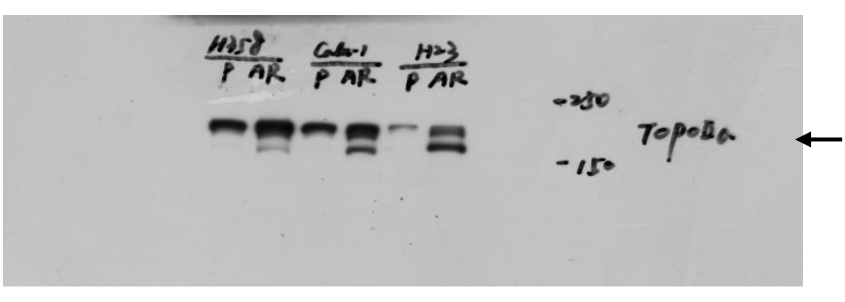

Full unedited gel for Figure 6B

FBXW7

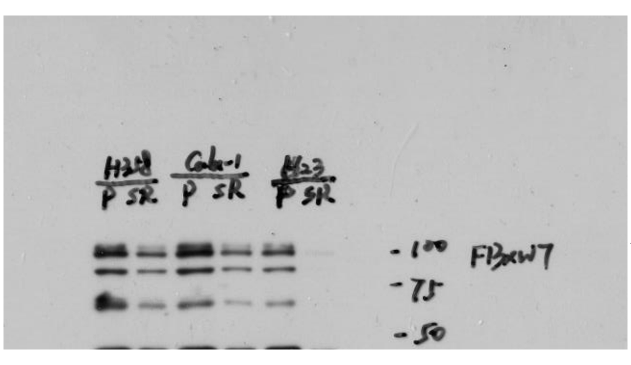

Full unedited gel for Figure 6B

GAPDH

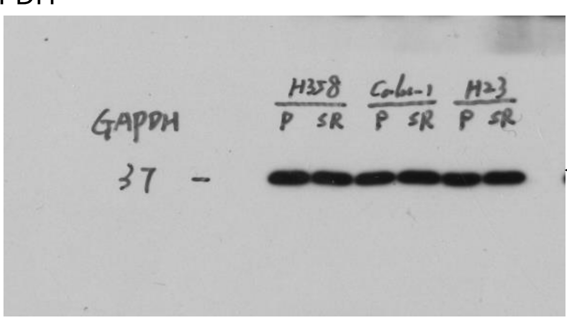

Full unedited gel for Figure 6B

GSK3

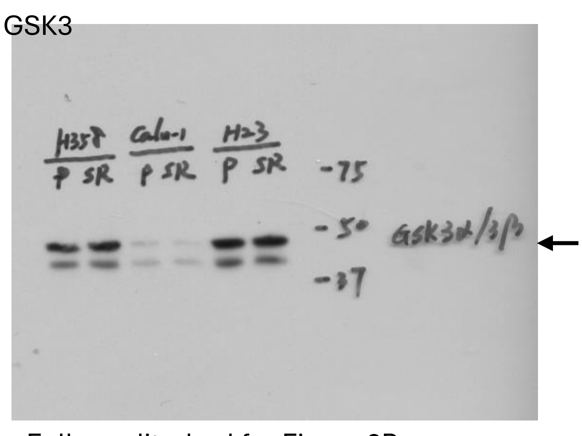

Full unedited gel for Figure 6B

p-GSK3

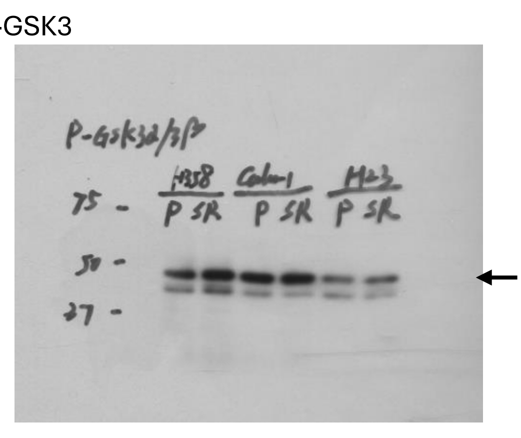

Full unedited gel for Figure 6B

GAPDH

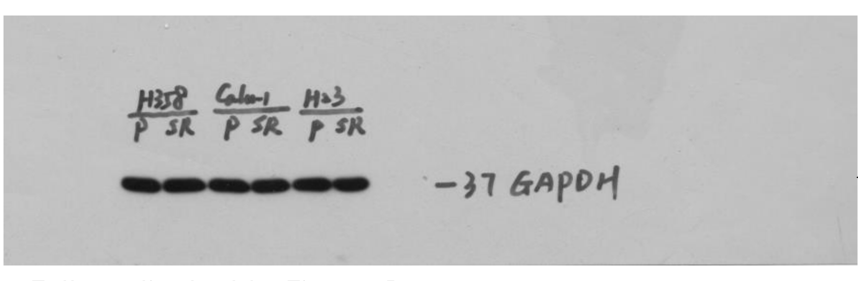

Full unedited gel for Figure 6B

Fig 6D

Topo IIa

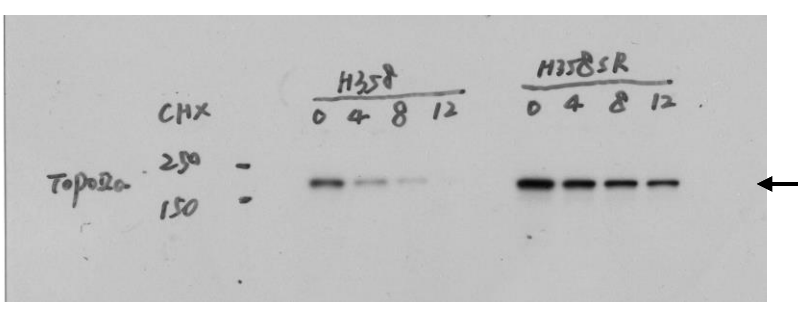

Full unedited gel for Figure 6D

GAPDH

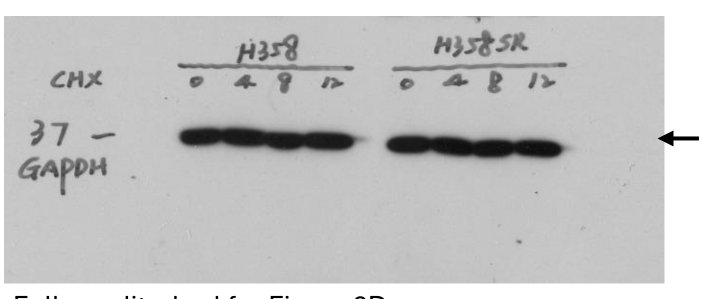

Full unedited gel for Figure 6D

Topo IIa

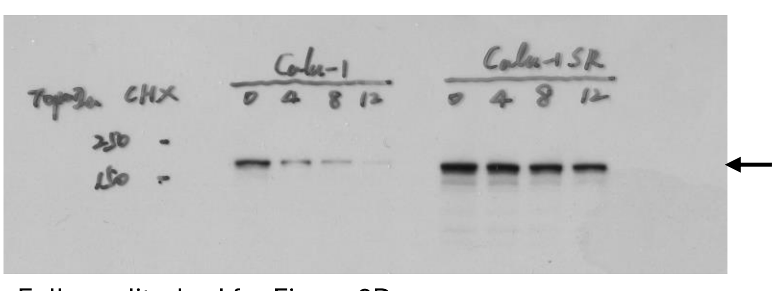

Full unedited gel for Figure 6D

GAPDH

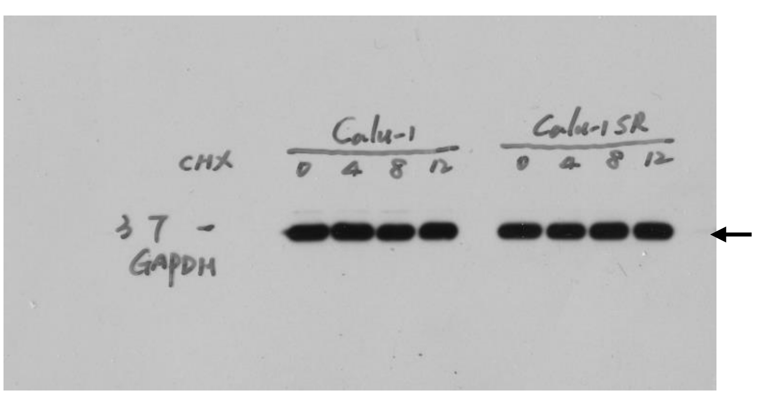

Full unedited gel for Figure 6D

Fig 6

Fig 7

PARP

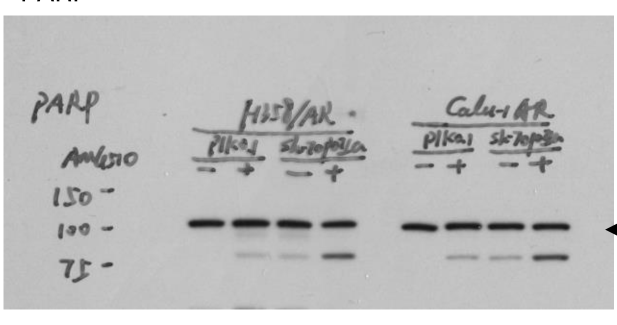

Full unedited gel for Figure 7A

Topo IIa

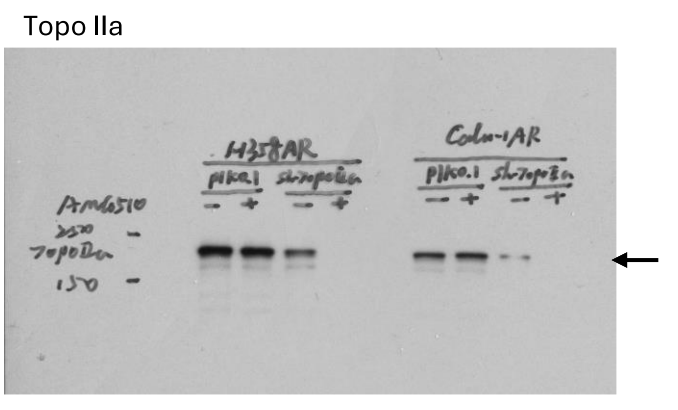

Full unedited gel for Figure 7A

Tubulin

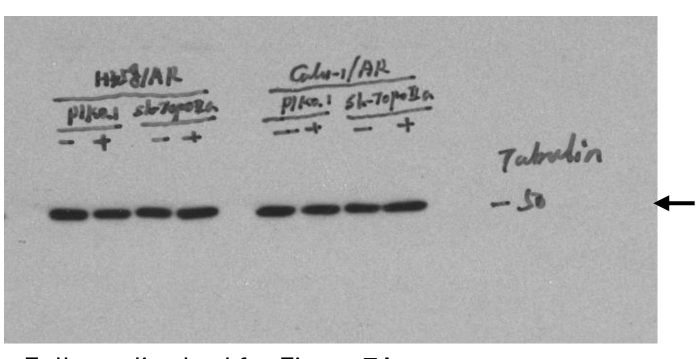

Full unedited gel for Figure 7A

Fig 7C

PARP

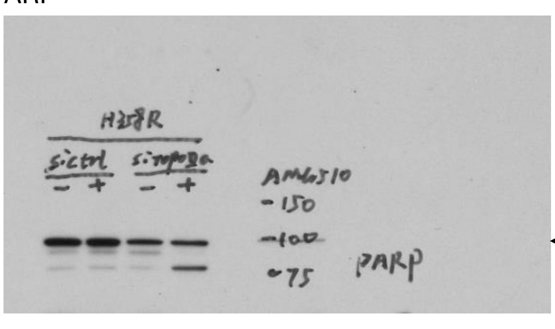

Full unedited gel for Figure 7C

PARP

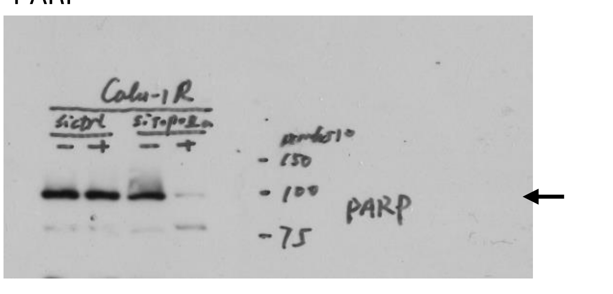

Full unedited gel for Figure 7C

Topo IIa

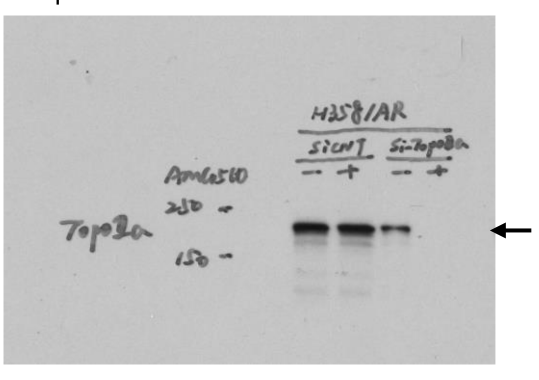

Full unedited gel for Figure 7C

Topo IIa

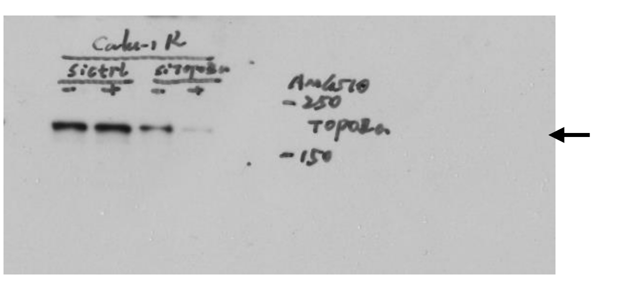

Full unedited gel for Figure 7C

Tubulin

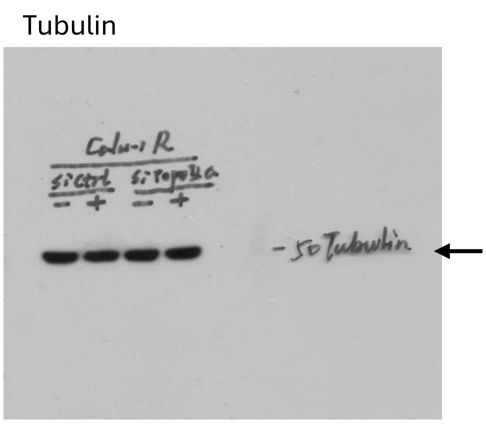

Full unedited gel for Figure 7C

Tubulin

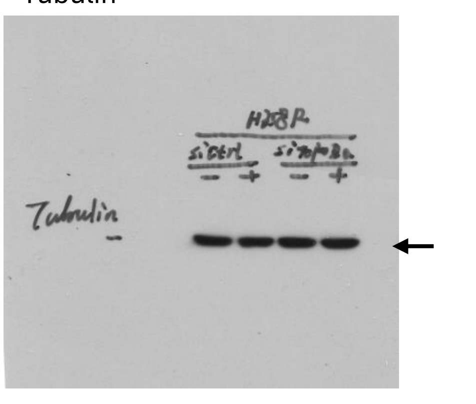

Full unedited gel for Figure 7C

Fig 7

Fig 8C

PARP

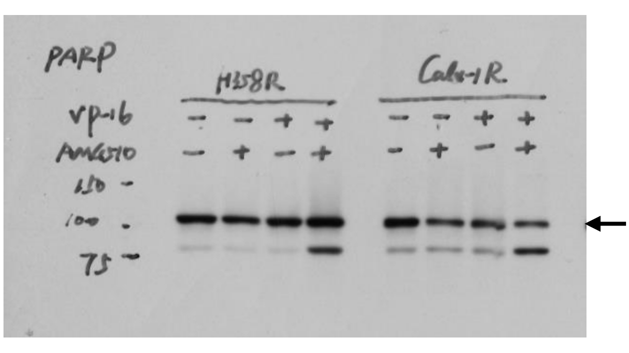

Full unedited gel for Figure 8C

Casp-3

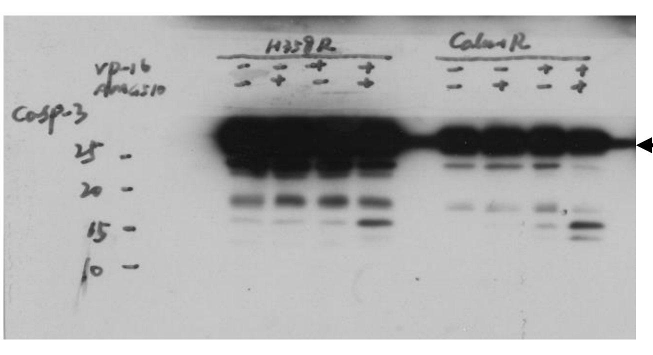

Full unedited gel for Figure 8C

Tubulin

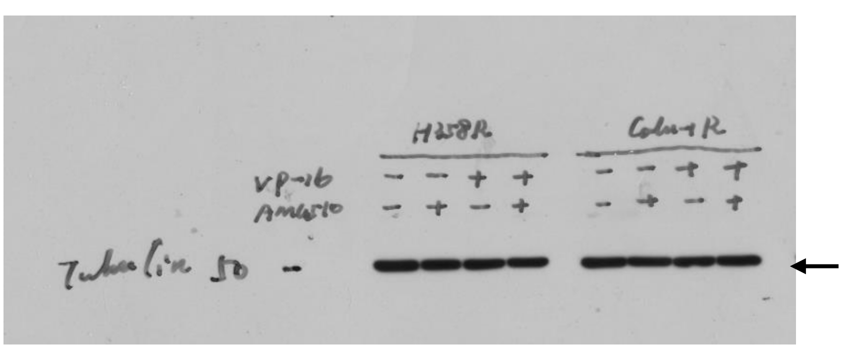

Full unedited gel for Figure 8C

Fig 8

Fig S1 A

Topo IIa

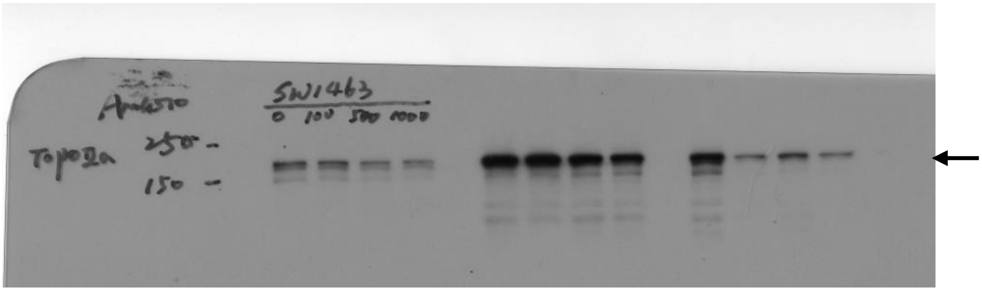

Full unedited gel for Figure S1A

Topo IIa

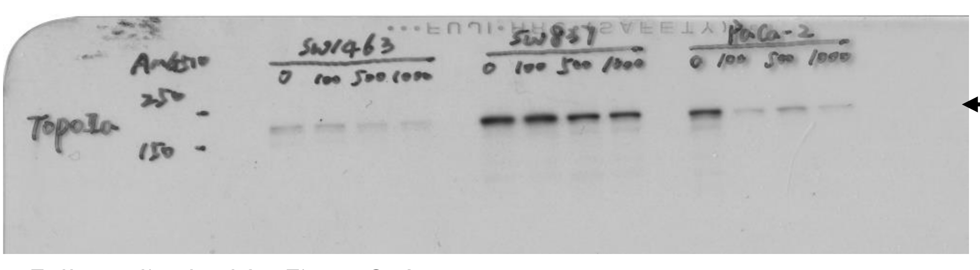

Full unedited gel for Figure S1A

GAPDH

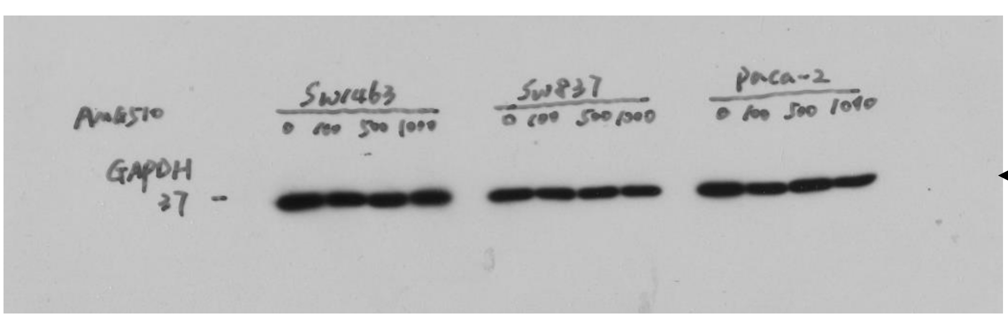

Full unedited gel for Figure S1A

Fig S1 B

Topo IIa

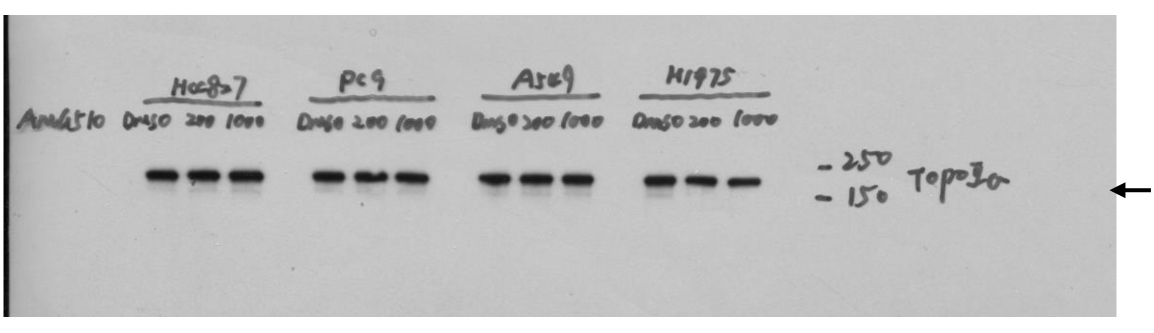

Full unedited gel for Figure S1B

Tubulin

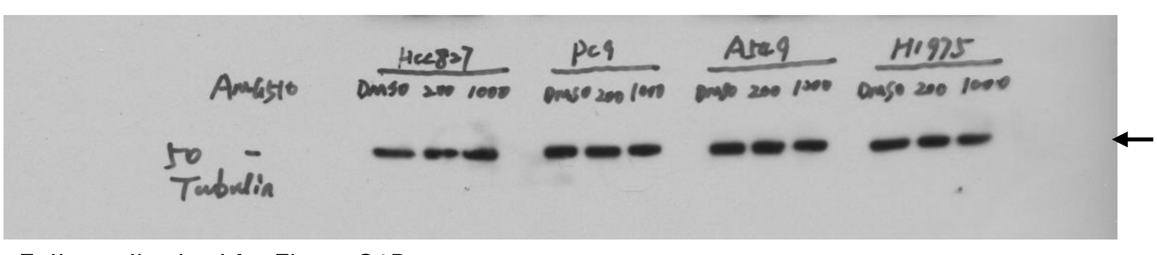

Full unedited gel for Figure S1B

Fig S1

Fig S5 A

Bim

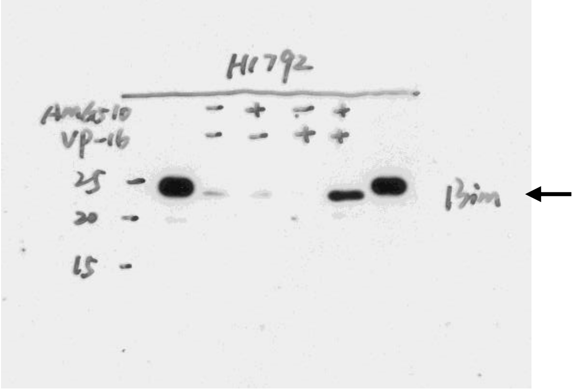

Full unedited gel for Figure S5A

Mcl-1

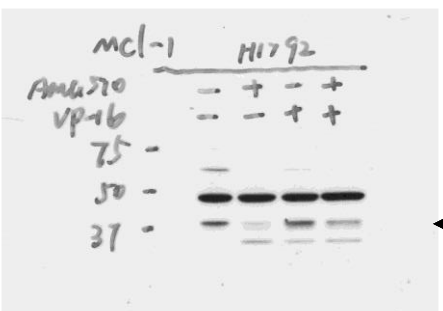

Full unedited gel for Figure S5A

Tubulin

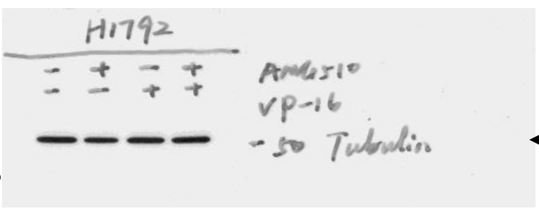

Full unedited gel for Figure S5A

PARP

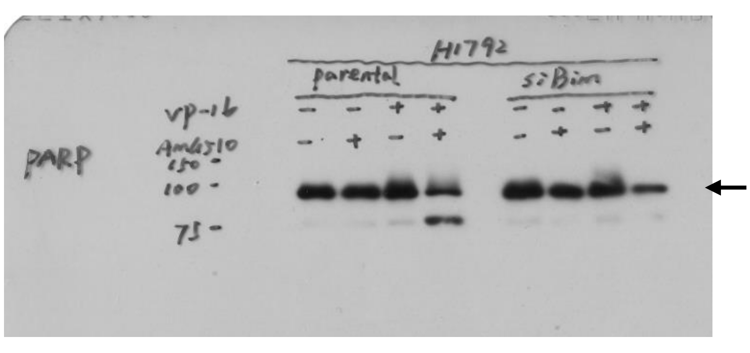

Full unedited gel for Figure S5B

Bim

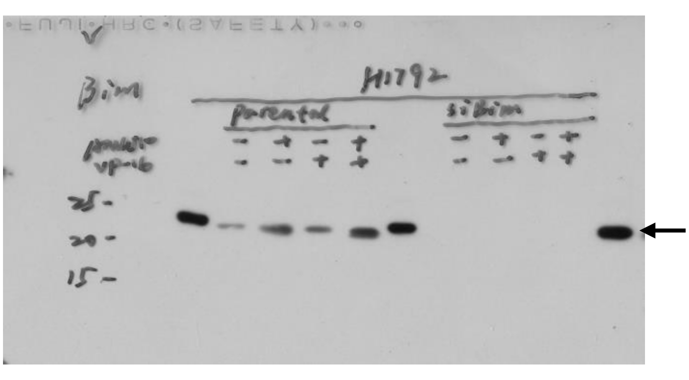

Full unedited gel for Figure S5B

GAPDH

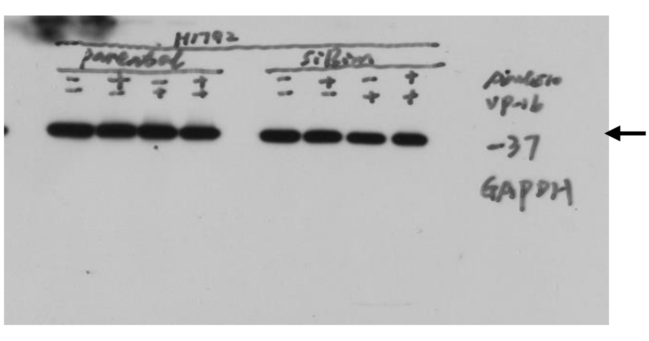

Full unedited gel for Figure S5B

Fig S5

Fig S6 C

Topo IIa

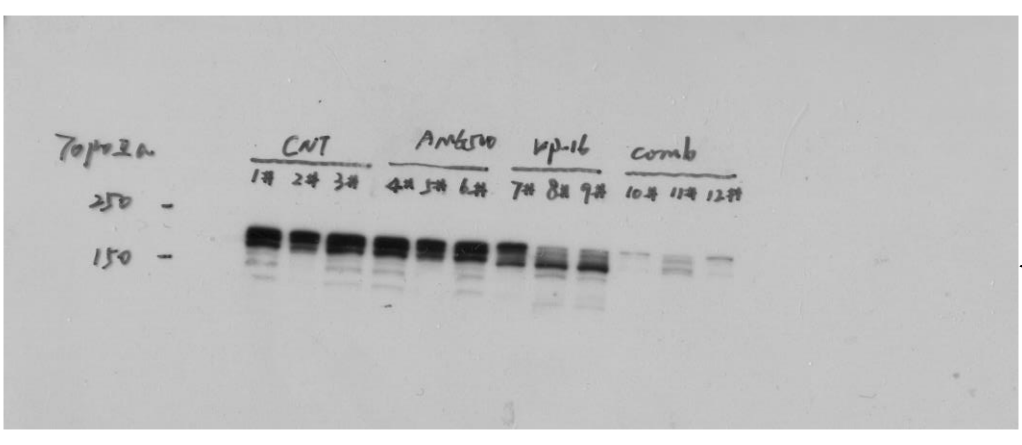

Full unedited gel for Figure S6C

GAPDH

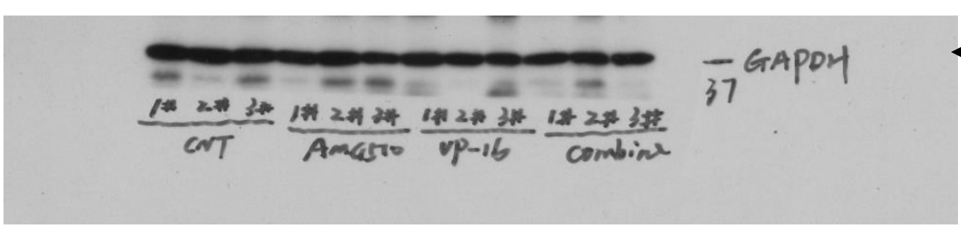

Full unedited gel for Figure S6C

Fig S6

Fig S10 B

PARP

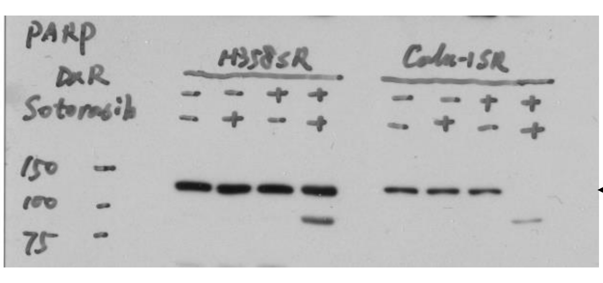

Full unedited gel for Figure S10B

Casp-3

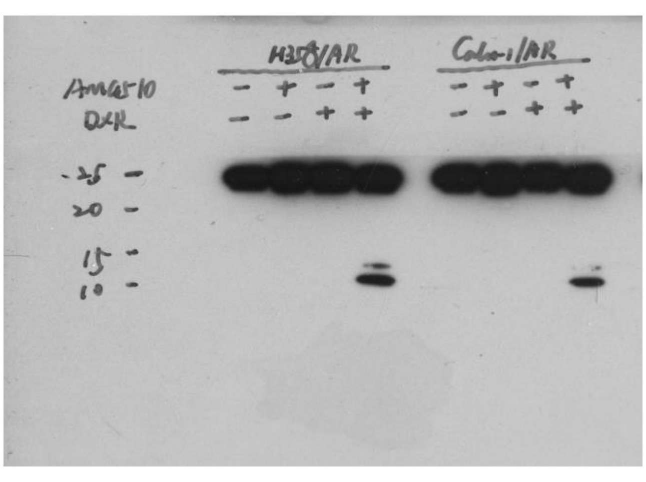

Full unedited gel for Figure S10B

Tubulin

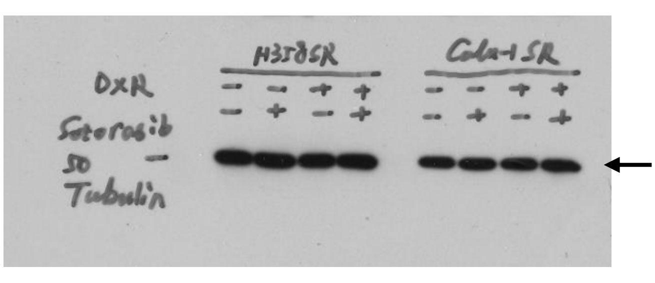

Full unedited gel for Figure S10B

Fig S10
